# Functionalization of N_2_ via Formal 1,3‐Haloboration of a Tungsten(0) σ‐Dinitrogen Complex

**DOI:** 10.1002/chem.202002678

**Published:** 2020-10-22

**Authors:** Anna Rempel, Soren K. Mellerup, Felipe Fantuzzi, Anselm Herzog, Andrea Deißenberger, Rüdiger Bertermann, Bernd Engels, Holger Braunschweig

**Affiliations:** ^1^ Institute for Sustainable Chemistry & Catalysis with Boron Julius-Maximilians-Universität Würzburg Am Hubland 97074 Würzburg Germany; ^2^ Institute for Inorganic Chemistry Julius-Maximilians-Universität Würzburg Am Hubland 97074 Würzburg Germany; ^3^ Institute for Physical and Theoretical Chemistry Julius-Maximilians-Universität Würzburg Emil-Fischer-Str. 42 97074 Würzburg Germany

**Keywords:** borylation, diazenido tungsten complexes, dinitrogen functionalization

## Abstract

Boron tribromide and aryldihaloboranes were found to undergo 1,3‐haloboration across one W−N≡N moiety of a group 6 end‐on dinitrogen complex (i.e. *trans*‐[W(N_2_)_2_(dppe)_2_]). The N‐borylated products consist of a reduced diazenido unit sandwiched between a W^II^ center and a trivalent boron substituent (W−N=N−BXAr), and have all been fully characterized by NMR and IR spectroscopy, elemental analysis, and single‐crystal X‐ray diffraction. Both the terminal N atom and boron center in the W−N=N−BXAr unit can be further derivatized using electrophiles and nucleophiles/Lewis bases, respectively. This mild reduction and functionalization of a weakly activated N_2_ ligand with boron halides is unprecedented, and hints at the possibility of generating value‐added nitrogen compounds directly from molecular dinitrogen.

## Graphical abstract

**Boron tribromide** and aryldihaloboranes were found to undergo 1,3‐haloboration across one W−N≡N moiety of a group 6 end‐on dinitrogen complex. Both the terminal N atom and boron center in the resulting diazenido W−N=N−BXAr unit can be further derivatized using electrophiles and nucleophiles/Lewis bases, respectively.

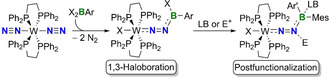

## Introduction

**Scheme 1:**
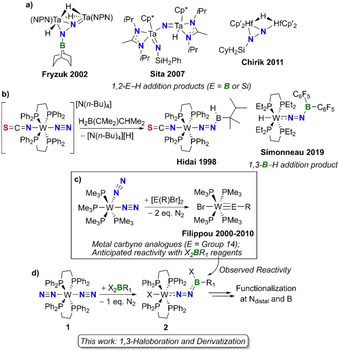
a) Examples of products obtained from 1,2‐B/Si−H addition across M−N bonds (NPN=(PhNSiMe_2_CH_2_)_2_PPh), Cp’=1,2,4‐trimethylcyclopentadienyl, Cp*=pentamethylcyclopentadienyl). b) 1,3‐B−H addition of Pier's borane across a Chatt‐Hidai tungsten complex. c) Synthesis of heavy metal carbyne analogues (E=Ge, Sn, Pb). d) This work: 1,3‐haloboration of *trans*‐[W(N_2_)_2_(dppe)_2_] and subsequent functionalization of both N_term_ and B (dppe=1,2‐bis(diphenylphosphino)ethane).

The reduction and functionalization of molecular dinitrogen (N_2_) at discrete transition metal centers continue to represent some of the most challenging chemical transformations, despite nearly 60 years of research in this area.^[1]^ Following the work of Allen and Senoff, who reported the first end‐on coordinated N_2_ transition metal complexes in 1965 (e.g. [Ru(NH_3_)_5_N_2_]^2+^),^[2]^ the groups of Chatt and Hidai established the synthesis of N_2_‐bound molybdenum and tungsten complexes,^[3]^ as well as the conversion of their N_2_ ligands to ammonia in the presence of Brønsted acids.^[4]^ Aside from reduction, the bound N_2_ ligands in such compounds can also react with various main‐group substrates, generating N‐functionalized moieties containing N−Al,^[5]^ N−B^[6]^ and N−Si[^6h^, ^7^] bonds. For example, reacting end‐on coordinated N_2_ compounds of Mo, W or Fe with B(C_6_F_5_)_3_ leads to Lewis acid‐base adduct formation between the highly electrophilic borane and terminal N atom of the dinitrogen complex.[^6e^, ^6h^] Conversely, reactions with hydroboranes and ‐silanes either lead to 1,2‐/1,3‐addition products[^6^, ^7^] or boryl/silyl amines (Scheme 1 a/b),[^6d^, ^6i^] driven by the hydridic character of the B/Si−H bonds and formation of strong N−B and N−Si bonds in the products. In fact, N−Si bond formation has become a popular strategy for converting dinitrogen into ammonia equivalents.[^7h^, ^7i^] With respect to metal‐free activation of N_2_, our group has shown that carbene‐stabilized borylenes are capable of capturing and reducing N_2_,^[8]^ while Stephan and co‐workers recently established that sterically encumbered diazomethanes can undergo 1,1‐hydroboration at the terminal N atom with Pier's borane (HB(C_6_F_5_)_2_) and form a stable diazomethane‐borane adduct with B(C_6_F_5_)_3_.^[9]^

Given our group's continued interest in transition metal borylene complexes,^[10]^ we were drawn to Chatt‐Hidai‐type tungsten N_2_ complexes (e.g. *trans*‐[W(N_2_)_2_(dppe)_2_]; **1** in Scheme 1) as possible neutral precursors for low valent metal‐boron species. Filippou and co‐workers have shown that certain *p*‐block element halides react with neutral group 6 N_2_ complexes (e.g. [W(PMe_3_)_4_(N_2_)_2_]; Scheme 1 c), forming transition metal carbyne analogues with M≡E triple bonds ([L_x_M≡E−R], M=Mo, W; E=Ge, Sn, and Pb).^[11]^ In these reactions, one E−X bond is oxidatively added across the metal center with concomitant liberation of N_2_, leading to the generation of main‐group element carbyne fragments. Although we anticipated a similar reactivity between boron halides and **1**, we instead observed 1,3‐haloboration across the W−N≡N unit of the metal complex, yielding the N‐borylated compounds **2** (Scheme 1 d). These transformations proceed with the use of either boron trihalides or aryldihaloboranes and represent the first examples of 1,3‐B−X functionalization (where X=Cl and Br) of a transition metal dinitrogen complex. Compounds **2** can be further derivatized, undergoing electrophilic substitution at nitrogen and either adduct formation with a Lewis base or nucleophilic substitution with aryl lithium reagents at boron. The synthetic, spectroscopic, and crystallographic details are presented.

## Results and Discussion

**Scheme 2:**
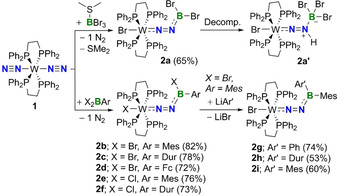
Synthesis of N‐borylated diazenido complexes of tungsten (**2 a**–**2 f**), as well as the decomposition product **2 a’** and nucleophilic substitution products **2 g**–**2 i**. Mes=2,4,6‐trimethylphenyl; Dur=2,3,5,6‐tetramethylphenyl; Fc=1‐ferrocenyl. Isolated yields are listed in brackets.

The N‐borylated diazenido‐tungsten complexes **2 a**–**2 f** were obtained in good yields (65–80 %; >95 % purity) by reacting the known tungsten dinitrogen complex **1** ^[12]^ with either Me_2_S⋅BBr_3_ or X_2_BAr (X=Br or Cl, Ar=Mes or Dur; Mes=2,4,6‐trimethylphenyl, Dur=2,3,5,6‐tetramethylphenyl) in benzene as shown in Scheme 2. The reaction time and temperature were dependent on the halogen bound to boron, with boron bromides achieving full conversion to **2 b**–**2 d** after five minutes of stirring at room temperature. Conversely, boron chlorides required heating at 60 °C for four hours in order to obtain the related products **2 e** and **2 f**. All six compounds tend to decompose in solution, with **2 b**–**2 f** showing signs of decomposition after a day in C_6_D_6_. Monitoring the ^1^H and ^31^P NMR spectra of **2 a** in C_6_D_6_ over the course of 1 hour revealed the formation of a second species **2 a’** as ≈5 % of the total mixture.^[6e]^ Compound **2 a’** was never isolated quantitatively due to the incomplete decomposition of **2 a**. The identity of **2 a’** was confirmed via single‐crystal X‐ray diffraction (sc‐XRD) experiments, where **2 a** and **2 a’** co‐crystallize in a ratio of 92:8 respectively (see Figure S101 in the Supporting Information). Formally, **2 a’** is the hydrobromination product of **2 a**, where the terminal N atom and boron center have been protonated and brominated, respectively. Although the source of the proton used to generate **2 a’** is unclear, it is worth mentioning that these reactions must be carried out in silanized or Teflon/PE reaction vessels to avoid decomposition. This indicates that the N‐borylated diazenido complexes of tungsten are highly susceptible to protonation. The analogous protonation products of **2 b**–**2 f** could also be observed spectroscopically, albeit in significantly lower quantities due to their slower decomposition. Complex **2 b** can be further functionalized at boron via nucleophilic substitution with organolithium reagents such as phenyl‐, duryl‐, and mesityllithium, yielding compounds **2 g**–**2 i** in moderate yield (50–75 %; >95 % purity). The isolated yields of these three reactions varied depending on which aryllithium reagent was used, with precursors with less bulky groups (i.e. Ph) leading to higher yields than those with bulkier groups (i.e. Dur and Mes). Attempts to prepare the same compounds using **1** and BrBAr_2_ were unsuccessful, as heating these reaction mixtures for 24 hours at 60 °C in C_6_D_6_ only gave partial conversion to the desired products. Except for **2 a’**, all of the new compounds were fully characterized by multinuclear NMR spectroscopy (^1^H, ^11^B, ^13^C, and ^31^P) and elemental analysis (EA). With the exception of **2 a**, the boron nucleus of which resonates at 6.72 ppm, no ^11^B NMR signals were observed for any of the other eight diazenido‐tungsten complexes in solution. The solid‐state ^11^B RSHE/MAS NMR spectrum of **2 b** revealed an isotrope chemical shift at δ_iso_=19.0 ppm, with a quadrupolar coupling constant of C_Q_=2.74 MHz and a quadrupolar asymmetry parameter *η*_Quad_=0.59. The upfield ^11^B chemical shift of **2 b** indicates significant electron density at boron, likely due to B−N double bond character (vide infra).^[13]^ The IR stretching frequency of the N_2_ unit in **2 a**–**2 i** was found to be 1500–1700 cm^−1^, which is consistent with a lower N_2_ bond order than in the parent dinitrogen complex (IR stretch ≈2000 cm^−1^).^[3a]^ Cyclic voltammetry (CV) measurements reveal irreversible reduction peaks at *E*_pc_=−2.26 and −2.11 V for **2 b** and **2 d**, respectively (Figure S17 and S34 in the SI), which are attributed to reduction of the tricoordinate boron center.^[14]^

**Figure 1:**
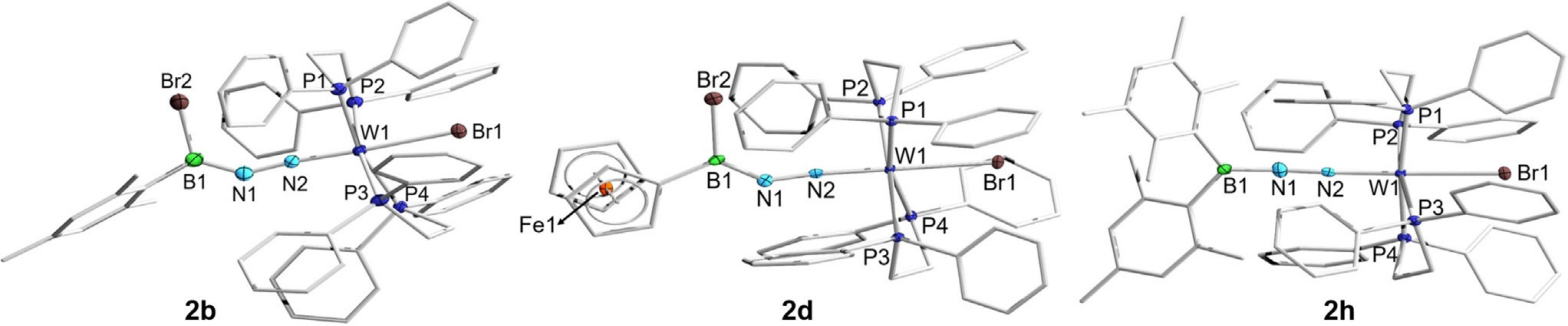
Single‐crystal X‐ray crystallographic structures of **2 b**, **2 d**, and **2 h**. Atomic displacement ellipsoids are depicted at 50 % probability and omitted at the ligand periphery. Hydrogen atoms are omitted for clarity. Selected bond lengths [Å] and angles [°]: **2 b** W1−N2 1.780(2), N1−N2 1.279(4), B1−N1 1.356(5), B1‐N1‐N2 141.5(3); **2 d**: W1−N2 1.793(4), N1−N2 1.256(6), B1−N1 1.381(7), B1‐N1‐N2 138.6(4); **2 h**: W1−N2 1.8353(19), N1−N2 1.226(3), B1−N1 1.392(3), B1‐N1‐N2 177.40(18).

Orange (**2 a**–**2 f**) and yellow‐green (**2 g**–**2 i**) single crystals suitable for X‐ray diffraction were obtained from concentrated benzene solutions at room temperature, with the X‐ray structures of **2 b**, **2 d**, and **2 h** shown in Figure 1. The structure of these 1,3‐haloboration products are comparable to the 1,3‐B−H addition product of Simonneau (Scheme 1 b),^[6c]^ with bent B1−N1−N2 angles of ≈140°. Despite the trigonal planar geometry at boron in **2 a**–**2 f**, all six compounds possess short B1−N1 bond lengths (e.g. 1.356(5) and 1.381(7) Å for **2 b** and **2 d**, respectively), which is characteristic of a boron‐nitrogen double bond.^[13]^ Compared to the parent complex **1**,^[15]^ compounds **2 a**–**2 f** have lengthened N1−N2 bonds (≈1.13→≈1.25 Å) and shortened W1−N2 bonds (≈1.99→≈1.80 Å), which is consistent with reduction of a dinitrogen ligand in **1** and previously reported N‐functionalized diazenido‐tungsten complexes.^[16]^ Replacing the halogen bound to boron in **2 b** with an aryl substituent causes an increase in the B1−N1−N2 angles of the resulting **2 g**–**2 i**, going from ≈142° in **2 b** to 146° and ≈175° in **2 g** and **2 h**/**2 i**, respectively. While the geometry of **2 g** is similar to **2 a**–**2 f**, both **2 h** and **2 i** exhibit a linear arrangement of their B1−N1−N2−W1 units, likely due to the presence of two bulky aryl groups on boron which clash with the phosphine ligands on tungsten. One plausible mechanism leading to the formation of the 1,3‐haloboration products **2 a**–**2 f** has been proposed by Simonneau et al. for the related 1,3‐B−H addition reaction.^[6c]^ Initially, a Lewis acid‐base adduct is formed between the borane and one terminal N atom of the tungsten dinitrogen complex. Next, a second equivalent of borane acts as a catalytic halide shuttle, transferring the halide bound to boron in the Lewis adduct to the bottom side of the W atom via an ionic intermediate.

To gain a better understanding of the bonding situation in the N‐borylated tungsten complexes **2 a**–**2 f**, geometry optimizations and frequency calculations were performed at the B3LYP‐D3(BJ)/def2‐SVP/SDD(W) level of theory^[17]^ for compounds **1**, **2 b** and **2 f**. These were followed by calculations using the energy decomposition analysis in conjunction with natural orbitals for chemical valence (EDA‐NOCV)^[18]^ at the B3LYP‐D3(BJ)/ZORA/TZ2P level.^[19]^ A summary of the EDA‐NOCV results is found in Table 1 (for more details, see SI). In all cases, Δ*E*_orb(1)_ and Δ*E*_orb(2)_ are orbital interaction contributions of π backdonation from W to the [N_2_BXAr] fragment, whereas Δ*E*_orb(3)_ is related to σ donation from N_2_ to W. The EDA‐NOCV analysis reveals that the W−N bonding in **2 b** and **2 f** are dominated by orbital interactions, which accounts for ca. 65 % of the total attractive contribution. The Δ*E*_orb_ terms indicate that the π backdonation contribution is significantly larger in the N‐borylated complexes **2 b** (82.3 % of Δ*E*_orb_) and **2 f** (82.5 % of Δ*E*_orb_) than in the parent compound **1** (63.7 % of Δ*E*_orb_). This larger (W→N_2_)π backdonation enhances the donor‐acceptor W−N interactions, but weakens the N−N bond. This is in agreement with X‐ray crystallographic data, where the calculated bond lengths and Mayer bond orders (MBOs)^[20]^ of **2 b** and **2 f** are consistent with a stronger W−N bond (**2 b**, **1**: MBOs=1.592, 0.688; W−N=1.804 Å, 2.025 Å, respectively) and a reduced diazenido fragment (**2 b**, **1**: MBOs=1.267, 2.344; N−N=1.245 Å, 1.133 Å, respectively).

**Table 1:** EDA‐NOCV results for **2 b** and **2 f**. Energy terms are given in kcal mol^−1^.

Energy Terms	**1** ^[c]^	**2 b** ^[c]^	**2 f** ^[c]^
Δ*E*_int_	−48.4	−323.7	−317.9
Δ*E*_Pauli_	111.3	246.0	245.3
Δ*E*_disp_^[a]^	−13.0 (8.1 %)	−33.5 (5.9 %)	−34.4 (6.1 %)
Δ*E*_elstat_^[a]^	−67.0 (42.0 %)	−159.5 (28.0 %)	−161.9 (28.7 %)
Δ*E*_orb_^[a]^	−79.6 (49.9 %)	−376.8 (66.1 %)	−366.9 (65.2 %)
Δ*E*_orb(1)_^[b]^ π_I_	−27.0 (33.8 %)	−171.9 (45.6 %)	−175.3 (47.8 %)
Δ*E*_orb(2)_^[b]^ π_⊥_	−23.8 (29.9 %)	−138.1 (36.7 %)	−127.2 (34.7 %)
Δ*E*_orb(3)_^[b]^ σ	−23.5 (29.5 %)	−36.0 (9.6 %)	−35.5 (9.7 %)
Δ*E*_orb(rest)_	−5.4 (6.8 %)	−30.8 (8.2 %)	−29.0 (7.9 %)

[a] The values in parentheses show the weight of each contribution with respect to the total attractive interaction. [b] The values in parentheses show the weight of each contribution with respect to the total orbital interaction, Δ*E*_orb_. [c] Fragments: [N_2_W]+N_2_ for **1**; [XW]^−^+[N_2_BXAr]^+^ for **2 b** and **2 f**.

**Figure 2:**
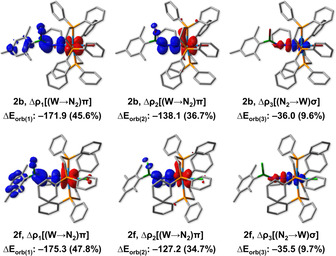
Deformation density plots of the three main bonding configurations that contribute to the total orbital interactions in the EDA‐NOCV description of **2 b** (top) and **2 f** (bottom) from [WX]^−^ and [N_2_BXAr]^+^ fragments. Isovalues: 0.0035 au. Charge flows from red to blue.

**Scheme 3:**
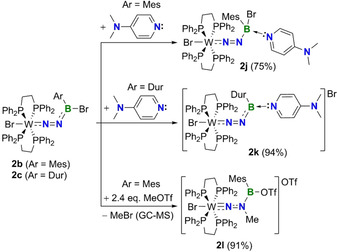
Reactivity of N‐borylated diazenido complexes of tungsten with a Lewis base (**2 b** and **2 c**) and electrophile (**2 b**). Isolated yields are listed in brackets.

In addition, inspection of the deformation densities (Figure 2) associated with Δ*E*_orb(1)_ reveals that this term is also related with donation of electron density into the π space of the adjacent B−N bond. Only Δ*E*_orb(1)_ has the correct symmetry to allow this form of extended backdonation, and might explain why its contribution is significantly larger than that of Δ*E*_orb(2)_. Accordingly, the calculated bond lengths and MBOs are also consistent with N(π)→B(*p*‐π) bonding (**2 b**: MBO=1.514; B−N=1.373 Å). To further test the reactivity of our newly synthesized N‐borylated diazenido‐tungsten complexes, we reacted compounds **2 b** and **2 c** with a neutral Lewis base (4‐dimethylaminopyridine; DMAP) as shown in Scheme 3. Depending on the substituent attached to boron, two different products are obtained. When the mesityl‐substituted **2 b** was reacted with DMAP, compound **2 j** featuring a Lewis acid‐base adduct between boron and DMAP was isolated in good yield (75 %). While single crystals suitable for X‐ray diffraction could not be obtained, the identity of **2 j** was confirmed via NMR spectroscopy and elemental analysis. Conversely, when DMAP was reacted with the duryl‐substituted **2 c**, the Lewis base replaced the halogen bound to boron, generating the borenium compound **2 k** in excellent yield (94 %).

**Figure 3:**
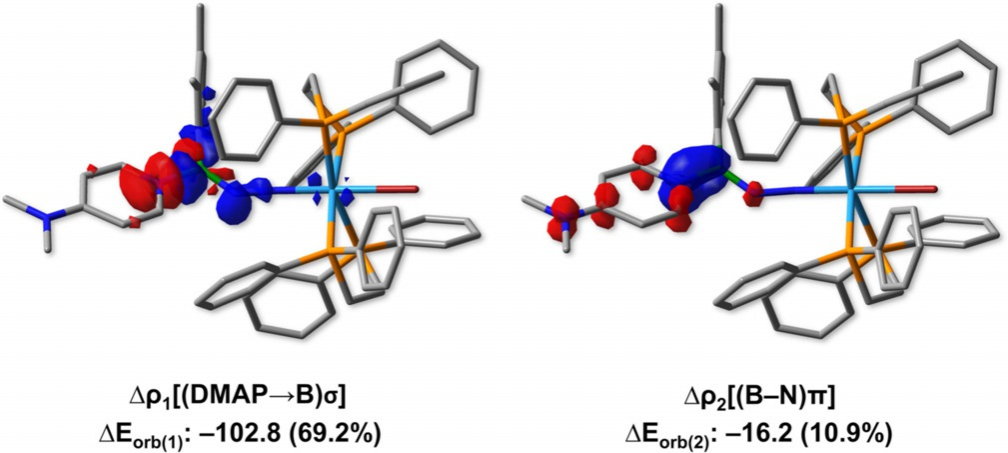
Deformation density plots of the two main bonding configurations that contribute to the total orbital interactions in the EDA‐NOCV description of **2 k** from [BrWN_2_BDur]^+^ and DMAP fragments. Isovalues: 0.0030 au. Charge flows from red to blue.

**Figure 4:**
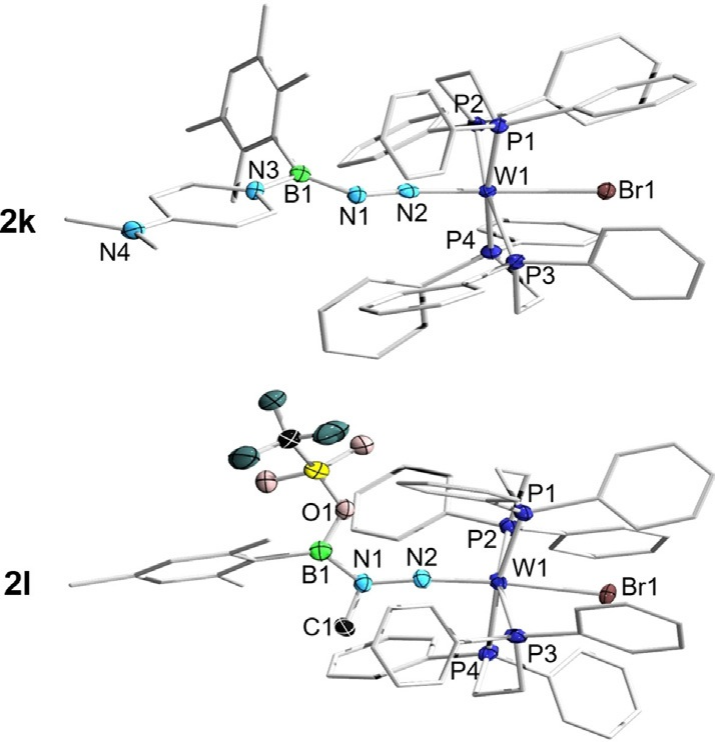
Single‐crystal X‐ray crystallographic structures of **2 k** and **2 l**. Atomic displacement ellipsoids are depicted at 50 % probability and omitted at the ligand periphery. Hydrogen atoms and counterions are omitted for clarity. Selected bond lengths [Å] and angles [°]: **2 k** W1−N2 1.797(3), N1−N2 1.283(5), B1−N1 1.377(6), B1−N3 1.543(6), B1−N1‐N2 135.0(4); **2 l**: W1−N2 1.766(3), N1−N2 1.380(4), B1−N1 1.402(6), N1−C1 1.491(5), B1‐N1‐N2 126.1(3).

As shown in Figure 3 and Figure 4, **2 k** is structurally similar to **2 b**, but with an elongated B1−N1 bond (1.377(6) Å) and bent B1‐N1‐N2 unit (135.0(4)°). The length of the B1−N3 bond in **2 k** is roughly halfway between those of typical B−N single and double bonds, indicating partial B=N character.^[13]^ Inspection of the deformation densities from EDA‐NOCV calculations of **2 k** (Figure 3) reveals that, while Δ*E*_orb(1)_ is related to dative bonding from the pyridyl nitrogen atom of DMAP to boron (69.2 %), Δ*E*_orb(2)_ suggests a π(B−N) interaction with small but non‐negligible character (10.9 %), which might contribute to the observed partial B=N character. Reacting **2 b** with two equivalents of methyl triflate (MeOTf) results in the formation of the cationic species **2 l**, where the terminal N has been methylated and the bromide attached to boron replaced by triflate. Monitoring the formation of **2 l** by gas chromatography—mass spectrometry (GC‐MS) revealed the presence of bromomethane in the reaction mixture. Purple crystals of **2 l** suitable for X‐ray diffraction analysis were obtained from the reaction mixture upon solvent removal, and its solid‐state structure is shown in Figure 4. Compound **2 l** has longer N1−N2 and B1−N1 bonds (1.380(4) and 1.402(6) Å, respectively) than **2 b**, with the former approaching the length of an N−N single bond (also supported by IR data; see Figure S15 and S98) and side‐on bound dinitrogen in early transition metal compounds.^[21]^ Additionally, the tungsten‐nitrogen distance in **2 l** is similar to that of compounds with a W−N triple bond.^[22]^ Despite the increase in B−N bond length in **2 l**, it is still in the range of a B=N double bond. The three reactivity modes highlighted in Scheme 3 illustrate the potential of this type of N‐borylated diazenido‐tungsten complex as a platform for the mild functionalization and derivatization of weakly‐activated dinitrogen.

## Conclusions

In summary, we have shown that the tungsten‐dinitrogen complex **1** reacts with boron trihalides and aryldihaloboranes under mild conditions, forming new N‐borylated diazenido‐tungsten species via formal 1,3‐haloboration across the W−N≡N moiety. The products of these reactions were identified by sc‐XRD, and fully characterized by NMR spectroscopy, elemental analysis, and computational energy decomposition analysis. The reactivity of these new N‐borylated diazenido‐tungsten complexes was also studied, revealing that such systems readily undergo derivatization at both the boron (nucleophilic substitution) and terminal nitrogen (electrophilic addition) atoms. Given the prevalence of metal‐boron complexes and their utility in a variety of useful chemical transformations (e.g. metal‐catalyzed C−H borylations), we believe that this mild functionalization of a weakly activated N_2_ ligand could pave the way for new types of catalytic processes or as an entry point for the preparation of value‐added nitrogen compounds. Attempts to expand this reactivity to different metal complexes and boron reagents is currently underway in our laboratory.

## Experimental Section

All reactions were performed under an atmosphere of dry argon using glovebox techniques in either PE vials, Teflon vials or silanized glass vessels (to prevent hydrolysis of the products). C_6_D_6_ and CD_2_Cl_2_ were degassed by three freeze‐pump‐thaw cycles and dried/stored over 4 Å molecular sieves. All other solvents were distilled/degassed from appropriate drying agents and stored over 4 Å molecular sieves. Compound **1**,^[12]^ BBr_2_Dur,^[23]^ BCl_2_Dur,^[23]^ BBr_2_Mes,^[24]^ BCl_2_Mes^[24]^ and BBr_2_Fc^[25]^ were synthesized according to literature procedures. All other chemicals were purchased from either Sigma–Aldrich, Acros or TCI Chemical Co. and used as received unless otherwise specified. NMR spectra were obtained from a Bruker Avance I 500 spectrometer (^1^H: 500.1 MHz, ^13^C{^1^H}: 125.8 MHz; ^31^P{^1^H}: 202.5 MHz; ^11^B: 160.5 MHz, ^19^F: 470.6 MHz) or a Bruker Avance I 400 spectrometer (^1^H: 400.1 MHz, ^13^C{^1^H}: 100.6 MHz; ^31^P{^1^H}: 162.0 MHz ^11^B: 128.4 MHz, ^19^F: 376.5 MHz) at 298 K unless stated otherwise. Chemical shifts (*δ*) are given in ppm and were internally referenced to the carbon nuclei (^13^C{^1^H}) or residual protons (^1^H) of the solvent. ^31^P, ^11^B and ^19^F NMR spectra were referenced to external standard 85 % H_3_PO_4_, [BF_3_⋅OEt_2_] or CFCl_3_, respectively. The solid‐state ^31^P{^1^H} CP/MAS and ^11^B{^1^H} RSHE/MAS (CP=cross polarization, RSHE=rotor synchronized Hahn‐Echo, MAS=magic‐angle spinning) NMR spectra of compound **2 b** were recorded using a Bruker Avance Neo 400 spectrometer operating at 162.0 MHz for ^31^P, 128.4 MHz for ^11^B using a 4 mm (o. d.) ZrO_2_ rotor at a spinning frequency of 14.5 kHz. Chemical shifts were calibrated externally using adamantane, and adjusting the field such that the ^13^C low‐field peak appears at 38.48 ppm. UV/Vis spectra were measured on a JASCO V‐660 or Mettler‐Toledo UV5 spectrometer. IR spectra were recorded with a Bruker Alpha spectrometer with an apodized resolution of 1 cm^−1^ in the attenuated total reflection (ATR) mode in the region of 4000–400 cm^−1^ using a setup with a diamond crystal. Microanalyses (C, H, N, S) were performed on an Elementar vario MICRO cube elemental analyzer. Cyclic voltammetry experiments were performed using a Gamry Instruments Reference 600 potentiostat. A standard three‐electrode cell configuration was employed using a platinum disk working electrode, a platinum wire counter‐electrode, and a silver wire, separated by a *Vycor* tip, serving as the reference electrode. Tetra‐*n*‐butylammonium hexafluorophosphate ([*n*Bu_4_N][PF_6_]) was employed as the supporting electrolyte. Compensation for resistive losses (*iR* drop) was employed for all measurements. Formal redox potentials are referenced to the ferrocene/ferrocenium ([Cp_2_Fe]^+/0^) redox couple.

### Synthetic procedures

*Synthesis of **2 a***: Compound **1** (30 mg, 29 μmol) was suspended in benzene (0.6 mL) at ambient temperature and Me_2_S⋅BBr_3_ (20 mg, 63 μmol) was added to give a red solution. The reaction mixture was stirred at room temperature for 5 min. The solution was subsequently treated with pentane (2 mL), at which point an orange solid precipitated. After removing the liquid phase, the solids were washed with pentane (3×4 mL), and all volatiles were evaporated at ambient temperature to afford **2 a** in 65 % yield (19 mg, 15 μmol). Single crystals suitable for X‐ray diffraction were grown from a saturated benzene solution. *NOTE*: **2 a** slowly decomposes in solution to **2 a’**. The presence of the decomposition product can be observed by NMR spectroscopy and X‐ray diffraction (see Figures S101). ^1^H NMR (C_6_D_6_, 500.1 MHz): *δ*=7.56–7.52 (m, 8 H, *o*‐C*H* Ph), 7.21–7.17 (m, 16 H, *o*‐C*H* Ph *+ m*‐C*H* Ph), 7.10 (t, ^3^ *J*_HH_=7.3 Hz, 4 H, *p*‐C*H* Ph), 6.94 (t, ^3^ *J*_HH_=7.3 Hz, 4 H, *p*‐C*H* Ph), 6.85 (t, ^3^ *J*_HH_=7.6 Hz, 8 H, *m*‐C*H* Ph), 2.33–2.61 (m, 4 H, C*H*_2_), 2.54–2.43 (m, 4 H, C*H*_2)_  ppm. ^13^C{^1^H} NMR (C_6_D_6_, 125.8 MHz): 136.7 (m, P*C*_q_), 136.1 (m, P*C*_q_), 134.4 (m, *o‐C*H Ph), 134.1 (m, *o‐C*H Ph), 130.3 (s, *p‐C*H Ph), 129.2 (s, *p‐C*H Ph), 128.8 (m, *m‐C*H Ph), 127.7 (*m‐C*H Ph, overlapping by solvent, identified by HSQC), 32.2–32.1 (m, P*C*H_2_)  ppm. ^11^B{^1^H} NMR (CD_2_Cl_2_, 160.5 MHz): 6.72 ppm. ^31^P{^1^H} NMR (C_6_D_6_, 202.5 MHz): *δ*=32.2 (s, ^1^ *J*_WP_=287 Hz) ppm. Elemental analysis for [C_52_H_48_BBr_3_N_2_P_4_W] (M_W_=1259.23): calcd (%). C 49.60, H 3.84 N 2.22; found (%) C 49.60, H 4.00, N 2.34. IR (solid): *ṽ*(NN)=1567 cm^−1^. UV/Vis (benzene): *λ*_max_=310, 360 nm.

*Synthesis of **2 b***: Compound **1** (40 mg, 39 μmol) was suspended in benzene (1 mL) at ambient temperature and BBr_2_Mes (15 mg, 52 μmol) was added to give a red solution. The reaction mixture was stirred at room temperature for 5 min. The solution was subsequently treated with pentane (2 mL), at which point an orange solid precipitated. After removing the liquid phase, the solids were washed with pentane (3×4 mL), and all volatiles were evaporated at ambient temperature to afford **2 b** in 82 % yield (41 mg, 32 μmol). Single crystals suitable for X‐ray diffraction were grown from a saturated benzene solution. ^1^H NMR (C_6_D_6_, 500.1 MHz): *δ*=7.91–7.85 (m, 8 H, *o*‐C*H* Ph), 7.10 (t, ^3^ *J*_HH_=7.5 Hz, 8 H, *m*‐C*H* Ph), 7.04 (t, ^3^ *J*_HH_=7.2 Hz, 4 H, *p*‐C*H* Ph), 6.92–6.98 (m, 14 H, *o*‐C*H* Ph + *p*‐C*H* Ph *+* C*H* Mes), 6.87 (t, ^3^ *J*_HH_=7.5 Hz, 8 H, *m*‐C*H* Ph), 2.86–2.71 (m, 4 H, CH_2_), 2.71–2.58 (m, 4 H, CH_2_), 2.57 (s, 6 H, C*H*_3_), 2.31 (s, 3 H, C*H*_3)_  ppm. ^13^C{^1^H} NMR (C_6_D_6_, 125.8 MHz): *δ*=141.2 (B*C*_q_, identified by HMBC), 139.2–138.9 (m, P*C*_q_), 138.7 (s, *C*_q_ Mes), 136.8–136.5 (m, P*C*_q_), 136.2 (s, *C*_q_ Mes), 135.0 (m, *o‐C*H Ph), 134.3 (m, *o‐C*H Ph), 130.0 (s, *p‐C*H Ph), 128.9 (s, *p‐C*H Ph), 128.5 (m, *m‐C*H Ph), 127.4 (m, *m‐C*H Ph), 127.4 (s, *C*H Mes), 34.3–34.1 (m, P*C*H_2_), 23.9 (s, *C*H_3_ Mes), 21.5 (s, *C*H_3_ Mes) ppm. ^11^B NMR (C_6_D_6_, 160.5 MHz): not detected. Solid‐state ^11^B{^1^H} RSHE/MAS NMR (128 MHz): isotrope chemical shift *δ*_iso_=19.0 ppm, quadrupole coupling constant C_Q_=2.74 MHz, quadrupolar asymmetry parameter *η*_Quad_=0.59. ^31^P{^1^H} NMR (C_6_D_6_, 202.5 MHz): *δ*=37.9 (s, ^1^ *J*_WP_=288 Hz) ppm. Solid‐state ^31^P{^1^H} CP/MAS NMR (162.0 MHz): *δ*=45.2, 36.4, 34.0, 21.0 ppm. Elemental analysis for [C_61_H_59_BBr_2_N_2_P_4_W] (M_W_=1298.51): calcd (%). C 56.42, H 4.58, N 2.16; found (%) C 56.20, H 4.77, N 2.04. IR (solid): *ṽ*(NN)=1541 cm^−1^. UV/Vis (benzene): *λ*_max_=354 nm. CV (*o*‐C_6_H_4_F_2_, 293 K): first oxidation: *E*_pa_=+0.18 V, second oxidation: *E*_1/2_=+0.35 V, third oxidation: *E*_pa_=+0.52 V, first reduction: *E*_pc_=−2.26 V.

*Synthesis of **2 c***: Compound **1** (40 mg, 39 μmol) was suspended in benzene (1 mL) at ambient temperature and BBr_2_Dur (15 mg, 49 μmol) was added to give a red solution. The reaction mixture was stirred at room temperature for 5 min. The solution was subsequently treated with hexane (2 mL), at which point an orange solid precipitated. After removing the liquid phase, the solids were washed with hexane (3×4 mL), and all volatiles were evaporated at ambient temperature to afford **2 c** in 78 % yield (40 mg, 30 μmol). Single crystals suitable for X‐ray diffraction were grown from a saturated benzene solution. ^1^H NMR (C_6_D_6_, 500.1 MHz): *δ*=7.88 (br s, 8 H, *o*‐C*H* Ph), 7.10–7.03 (m, 12 H, *m*‐C*H* Ph + *p*‐C*H* Ph), 7.00 (s, 1 H, C*H* Dur, overlapping by satellites of solvent), 6.98–6.93 (m, 12 H, *o*‐C*H* Ph + *p*‐C*H* Ph), 6.87 (t, ^3^ *J*_HH_=7.5 Hz, 8 H, *m*‐C*H* Ph), 2.85–2.75 (br, 4 H, C*H*_2_), 2.70–2.60 (m, 4 H, C*H*_2_), 2.44 (s, 6 H, C*H*_3_), 2.30 (s, 6 H, C*H*_3_)  ppm. ^13^C{^1^H} NMR (C_6_D_6_, 125.8 MHz): *δ*=141.5 (s, B*C*_q_), 139.4 (P*C*_q_, identified by HMBC), 137.1 (P*C*_q_, identified by HMBC), 135.0 (m, *o‐C*H Ph), 134.4 (s, *C*_q_ Dur), 134.2 (m, *o‐C*H Ph), 132.5 (s, *C*_q_ Dur), 130.8 (s, *C*H Dur), 130.0 (s, *p‐C*H Ph), 128.9 (s, *p‐C*H Ph), 128.5 (m, *m‐C*H Ph), 127.5 (m, *m‐C*H Ph), 34.1 (m, P*C*H_2_), 20.9 (s, *C*H_3_), 20.1 (s, *C*H_3_)  ppm. ^11^B NMR (C_6_D_6_, 160.5 MHz): not detected. ^31^P{^1^H} NMR (C_6_D_6_, 202.5 MHz): *δ*=37.9 (s, ^1^ *J*_WP_=289 Hz) ppm. Elemental analysis for [C_62_H_61_BCl_2_N_2_P_4_W] (M_W_=1223.61): calcd (%) C 56.74, H 4.68 N 2.13; found (%) C 56.65, H 4.70, N 2.01. IR (solid): *ṽ*(NN)=1546 cm^−1^. UV/Vis (benzene): *λ*_max_=350 nm.

*Synthesis of **2 d***: Compound **1** (30 mg, 29 μmol) was suspended in benzene (0.6 mL) at ambient temperature and BBr_2_Fc (13 mg, 37 μmol) was added to give a red solution. The reaction mixture was stirred at room temperature for 15 min. The solution was subsequently treated with hexane (2 mL), at which point an orange solid precipitated. After removing the liquid phase, the solids were washed with hexane (3×4 mL), and all volatiles were evaporated at ambient temperature to afford **2 d** in 72 % yield (28 mg, 21 μmol). Single crystals suitable for X‐ray diffraction were grown from a saturated benzene solution. ^1^H NMR (C_6_D_6_, 400.1 MHz): *δ*=7.83–7.78 (m, 8 H, *o*‐C*H* Ph), 7.20 (t, ^3^ *J*_HH_=7.4 Hz, 8 H, *m*‐C*H* Ph), 7.14–7.06 (m, 12 H, *o*‐C*H* Ph + *p*‐C*H* Ph), 6.94 (t, ^3^ *J*_HH_=7.1 Hz, 4 H, *p*‐C*H* Ph), 6.85 (t, ^3^ *J*_HH_=7.4 Hz, 8 H, *m*‐C*H* Ph), 4.70 (t, ^3^ *J*_HH_=1.7 Hz, 2 H, C*H* Cp), 4.38 (t, ^3^ *J*_HH_=1.7 Hz, 2 H, C*H* Cp), 4.27 (s, 5 H, C*H* Cp), 2.84–2.72 (m, 4 H, CH_2_), 2.70–2.57 (m, 4 H, CH_2)_  ppm. ^13^C{^1^H} NMR (C_6_D_6_, 100.6 MHz): *δ*=138.1 (P*C*_q_, identified by HMBC), 137.4 (P*C*_q_, identified by HMBC), 134.9 (m, *o‐C*H Ph), 134.1 (m, *o‐C*H Ph), 130.0 (s, *p‐C*H Ph), 128.8 (s, *p‐C*H Ph), 128.6 (m, *m‐C*H Ph), 127.6 (m, *m‐C*H Ph), 74.7 (s, *C*H Cp), 71.7 (s, *C*H Cp), 69.5 (s, *C*H Cp), 33.4–33.1 (m, P*C*H_2)_  ppm. ^11^B NMR (C_6_D_6_, 128.4 MHz): not detected. ^31^P{^1^H} NMR (C_6_D_6_, 162.0 MHz): *δ*=35.6 (s, ^1^ *J*_WP_=290 Hz) ppm. Elemental analysis for [C_62_H_57_BBr_2_N_2_P_4_WFe⋅(C_6_H_6_)] (M_W_=1442.46): calcd (%) C 56.62, H 4.40, N 1.94; found (%) C 57.25, H 4.45, N 1.92. IR (solid): *ṽ*(NN)=1524 cm^−1^. UV/Vis (benzene): *λ*_max_ = 300, 258, 440 nm. CV (*o*‐C_6_H_4_F_2_, 293 K): first oxidation: *E*_pa_ = −0.14 V, second oxidation: *E*_1/2_ = +0.22 V (with shoulder at *E*_pa_ ≈ +0.1 V), third oxidation: *E*_pa_ = +0.50 V, fourth oxidation *E*_pa_ = +0.68 V, first reduction: *E*_pc_ = −2.11 V.

*Synthesis of **2 e***: Compound **1** (30 mg, 29 μmol) was suspended in benzene (0.6 mL) at ambient temperature and BCl_2_Mes (7 mg, 40 μmol) was added. The reaction mixture was heated to 60 °C for 4 h, which afforded a red solution. The solution was subsequently treated with hexane (2 mL), at which point an orange solid precipitated. After removing the liquid phase, the solids were washed with hexane (3×4 mL), and all volatiles were evaporated at ambient temperature to afford **2 e** in 76 % yield (27 mg, 22 μmol). Single crystals suitable for X‐ray diffraction were grown from a saturated benzene solution. ^1^H NMR (C_6_D_6_, 500.1 MHz): *δ*=7.93 (br s, 8 H, *o*‐C*H* Ph), 7.10 (t, ^3^ *J*_HH_=7.5 Hz, 8 H, *m*‐C*H* Ph), 7.04 (t, ^3^ *J*_HH_=7.2 Hz, 4 H, *p*‐C*H* Ph), 6.96–6.93 (m, 6 H, C*H* Mes + *p*‐C*H* Ph), 6.89–8.83 (m, 16 H, *o*‐C*H* Ph), 2.76–2.63 (m, 4 H, C*H*_2_), 2.61 (s, 6 H, C*H*_3_), 2.59–2.49 (m, 4 H, C*H*_2_), 2.31 (s, 3 H, C*H*_3)_  ppm. ^13^C{^1^H} NMR (C_6_D_6_, 125.8 MHz): *δ*=140.1 (B*C*_q_, identified by HMBC), 139.2 (s, *C*_q_ Mes), 139.1–138.9 (m, P*C*_q_), 136.3–136.0 (m, P*C*_q_), 136.1 (*C*_q_ Mes, identified by HMBC), 135.0 (m, *o‐C*H Ph), 134.1 (m, *o‐C*H Ph), 130.0 (s, *p‐C*H Ph), 128.8 (s, *p‐C*H Ph), 128.5 (m, *m‐C*H Ph), 127.5 (m, *m‐C*H Ph), 127.3 (s, *C*H Mes), 33.5–33.3 (m, *C*H_2_), 23.9 (s, *C*H_3_), 21.5 (s, *C*H_3_)  ppm. ^11^B NMR (C_6_D_6_, 160.5 MHz): not detected. ^31^P{^1^H} NMR (C_6_D_6_, 202.5 MHz): *δ*=41.3 (s, ^1^ *J*_WP_=290 Hz) ppm. Elemental analysis for [C_61_H_59_BCl_2_N_2_P_4_W] (M_W_=1209.60): calcd (%) C 60.57, H 4.92, N 2.32; found (%) C 60.35, H 5.04, N 2.32. IR (solid state): *ṽ*(NN)=1519 cm^−1^. UV/Vis (benzene): *λ*_max_=334 nm.

*Synthesis of **2 f***: Compound **1** (30 mg, 29 μmol) was suspended in benzene (0.6 mL) at ambient temperature and BCl_2_Dur (7 mg, 30 μmol) was added. The reaction mixture was heated to 60 °C for 4 h, which afforded a red solution. The solution was subsequently treated with hexane (2 mL), at which point an orange solid precipitated. After removing the liquid phase, the solids were washed with hexane (3×4 mL), and all volatiles were evaporated at ambient temperature to afford **2 f** in 73 % yield (26 mg, 21 μmol). Single crystals suitable for X‐ray diffraction were grown from a saturated benzene solution. ^1^H NMR (C_6_D_6_, 500.1 MHz): *δ*=7.94 (br s, 8 H, *o*‐C*H* Ph), 7.10 (t, ^3^ *J*_HH_=7.5 Hz, 8 H, *m*‐C*H* Ph), 7.05 (t, ^3^ *J*_HH_=7.2 Hz, 4 H, *p*‐C*H* Ph), 7.01 (s, 1 H, C*H* Dur), 6.89 (t, ^3^ *J*_HH_=7.1 Hz, 4 H, *p*‐C*H* Ph), 6.91–6.84 (m, 16 H, *o*‐C*H* Ph + *m*‐C*H* Ph), 2.85–2.75 (m, 4 H, C*H*_2_), 2.70–2.60 (m, 4 H, C*H*_2_), 2.50 (s, 6 H, C*H*_3_), 2.31 (s, 6 H, C*H*_3)_  ppm. ^13^C{^1^H} NMR (C_6_D_6_, 125.8 MHz): *δ*=143.9 (B*C*_q_, identified by HMBC), 138.3 (P*C*_q_, identified by HMBC), 136.3 (P*C*_q_, identified by HMBC), 135.1 (m, *o‐C*H Ph), 134.8 (s, *C*_q_ Dur), 134.1 (m, *o‐C*H Ph), 132. 4 (s, *C*_q_ Dur), 130.7 (s, *C*H Dur), 130.0 (s, *p‐C*H Ph), 128.7(s, *p‐C*H Ph), 128.5 (m, *m‐C*H Ph), 127.5 (*m‐C*H Ph), 33.3 (m, P*C*H_2_), 20.9 (s, *C*H_3_), 20.2 (s, *C*H_3)_  ppm. ^11^B NMR (C_6_D_6_, 160.5 MHz): not detected. ^31^P{^1^H} NMR (C_6_D_6_, 202.5 MHz): *δ*=41.4 (s, ^1^ *J*_WP_=291 Hz) ppm. Elemental analysis for [C_62_H_61_BCl_2_N_2_P_4_W] (M_W_=1223.61): calcd (%) C 60.86, H 5.02, N 2.29; found (%) C 60.22, H 5.06, N 2.26. IR (solid): *ṽ*(NN)=1527 cm^−1^. UV/Vis (benzene): *λ*_max_=348 nm.

*Synthesis of **2 g***: Compound **2 b** (30 mg, 23 μmol) was suspended in benzene (1 mL) at ambient temperature and PhLi (2 mg, 20 μmol) was added to give a greenish‐brown solution. The reaction mixture was stirred at room temperature for 5 min. The LiBr was removed by filtration, and the solvent evaporated to give a yellow‐green solid. The solid was washed with hexane (3×4 mL) and all volatiles were evaporated at ambient temperature to afford **2 g** in 74 % yield (22 mg, 17 μmol). Single crystals suitable for X‐ray diffraction were grown from a saturated benzene solution. ^1^H NMR (C_6_D_6_, 500.1 MHz): *δ*=8.00–7.96 (m, 8 H, *o*‐C*H* Ph), 7.81–7.79 (m, 2 H, C*H* BPh), 7.31–7.28 (m, 1 H, C*H* BPh), 7.23–7.20 (m, 2 H, C*H* BPh), 7.09 (t, ^3^ *J*_HH_=7.5 Hz, 8 H, *m*‐C*H* Ph), 7.02 (t, ^3^ *J*_HH_=7.2 Hz, 4 H, *p*‐C*H* Ph), 6.97–6.93 (m, 8 H, *o*‐C*H* Ph), 6.92 (t, ^3^ *J*_HH_=7.2 Hz, 4 H, *p*‐C*H* Ph), 6.85 (t, ^3^ *J*_HH_=7.5 Hz, 8 H, *m*‐C*H* Ph), 6.57 (s, 2 H, C*H* Mes), 2.81–2.71 (m, 4 H, C*H*_2_), 2.23 (s, 3 H, C*H*_3_), 2.17–2.06 (m, 4 H, C*H*_2_), 1.83 (s, 6 H, C*H*_3_)  ppm. ^13^C{^1^H} NMR (C_6_D_6_, 125.8 MHz): *δ*=145.8 (B*C*_q_ Ph, identified by HMBC), 144.7 (B*C*_q_ Mes, identified by HMBC),140.0–139.8 (m, P*C*_q_), 139.2 (s, *C*_q_ Mes), 137.8–137.6 (m, P*C*_q_), 135.4 (m, *o‐C*H PPh), 135.1 (s, *C*_q_ Mes), 134.7 (s, *C*H BPh), 134.2 (m, *o‐C*H PPh), 129.9 (s, *p‐C*H PPh), 128.8 (s, *C*H BPh), 128.6 (s, *p‐C*H PPh), 128.5 (m, *m‐C*H PPh), 127.7 (s, *C*H BPh), 127.7 (m, *m‐C*H PPh), 127.0 (s, *C*H Mes), 34.1–33.8 (m, P*C*H_2_), 22.7 (s, *C*H_3_ Mes), 21.3 (s, *C*H_3_ Mes) ppm. ^11^B NMR (C_6_D_6_, 160.5 MHz): not detected. ^31^P{^1^H} NMR (C_6_D_6_, 202.5 MHz): *δ*=39.9 (s, ^1^ *J*_WP_=292 Hz) ppm. Elemental analysis for [C_67_H_64_BBrN_2_P_4_W] (M_W_=1295.71): calcd (%) C 62.11, H 4.98, N 2.16; found (%) C 61.72, H 5.12, N 2.31. IR (solid): *ṽ*(NN)=1565 cm^−1^. UV/Vis (benzene): *λ*_max_=311, 396 nm.

*Synthesis of **2 h***: Compound **2 b** (20 mg, 15 μmol) was suspended in benzene (0.6 mL) at ambient temperature and DurLi (4 mg, 30 μmol) was added. The reaction mixture was heated at 60 °C for 3 h to afford a greenish‐brown solution. The LiBr was removed by filtration, and the solvent evaporated to give a yellow‐green solid. The solid was washed with hexane (3×4 mL), and all volatiles were evaporated at ambient temperature to afford **2 h** in 53 % yield (11 mg, 8 μmol). Single crystals suitable for X‐ray diffraction were grown from a saturated benzene solution. ^1^H NMR (C_6_D_6_, 500.1 MHz): *δ*=7.52–7.49 (m, 8 H, *o*‐C*H* Ph), 7.08–7.01 (m, 12 H, *o*‐C*H* Ph + *p*‐C*H* Ph), 6.98 (s, 1 H, C*H* Dur), 6.92 (t, ^3^ *J*_HH_=7.3 Hz, 4 H, *p*‐C*H* Ph), 6.85 (t, ^3^ *J*_HH_=7.4 Hz, 8 H, *m*‐C*H* Ph), 6.80 (t, ^3^ *J*_HH_=7.4 Hz, 8 H, *m*‐C*H* Ph), 6.76 (s, 2 H, C*H* Mes), 2.81–2.66 (m, 4 H, CH_2_), 2.66–2.51 (m, 4 H, C*H*_2_), 2.24 (s, 3 H, C*H*_3_ Mes), 2.28 (s, 6 H, C*H*_3_ Dur), 1.93 (s, 6 H, C*H*_3_ Mes), 1.91 (s, 6 H, C*H*_3_ Dur) ppm. ^13^C{^1^H} NMR (C_6_D_6_, 125.8 MHz): 150.2 (B*C*_q_ Dur, identified by HMBC), 147.1 (B*C*_q_ Mes, identified by HMBC), 141.0 (s, *C*_q_ Mes), 138.6–138.3 (m, P*C*_q_), 137.3–136.9 (m, P*C*_q_), 136.5 (s, *C*_q_ Dur), 135.7 (s, *C*_q_ Mes), 134.4 (m, *o‐C*H Ph), 134.2 (m, *o‐C*H Ph), 133.0 (s, *C*_q_ Dur), 130.7 (s, *C*H Dur), 129.5 (s, *p‐C*H Ph), 128.7 (s, *C*H Mes), 128.7 (s, *p‐C*H Ph), 128.6 (m, *m‐C*H Ph), 127.3 (m, *m‐C*H Ph),) 30.3–30.2 (m, P*C*H_2_), 23.3 (s, *C*H_3_ Mes), 21.3 (s, *C*H_3_ Mes), 20.6 (s, *C*H_3_ Dur), 19.8 (s, *C*H_3_ Dur) ppm. ^11^B NMR (C_6_D_6_, 160.5 MHz): not detected. ^31^P{^1^H} NMR (C_6_D_6_, 202.5 MHz): *δ*=32.40 (s, ^1^ *J*_WP_=292 Hz) ppm. Elemental analysis for [C_71_H_72_BBrN_2_P_4_W] (M_W_=1351.82): calcd (%) C 63.08, H 5.37, N 2.07; found (%) C 63.15, H 5.39, N 1.86. IR (solid): *ṽ*(NN)=1655 cm^−1^. UV/Vis (benzene): *λ*_max_=313, 400 (shoulder) nm.

*Synthesis of **2 i***: Compound **2 b** (30 mg, 23 μmol) was suspended in benzene (0.6 mL) at ambient temperature and MesLi (6 mg, 47 μmol) was added. The reaction mixture was heated at 60 °C for 3 h to afford a green‐brown solution. The LiBr was removed by filtration, and the solvent evaporated to give a yellow‐green solid. The solid was washed with hexane (3×4 mL), and all volatiles were evaporated at ambient temperature to afford **2 i** in 60 % yield (18 mg, 14 μmol). Single crystals suitable for X‐ray diffraction were grown from a saturated benzene solution. An alternative synthesis was attempted by reacting **1** (30 mg, 29 μmol) with BBrMes_2_ (12 mg, 36 μmol) in C_6_D_6_ (0.6 mL). The solution was heated for 24 h at 60 °C, after which ^1^H NMR spectroscopy revealed an inseparable mixture of **1** and **2 i**. ^1^H NMR (C_6_D_6_, 500.1 MHz): *δ*=7.51–7.48 (m, 8 H, *o*‐C*H* Ph), 7.08–7.04 (m, 8 H, *o*‐C*H* Ph), 7.02 (t, ^3^ *J*_HH_=7.4 Hz, 4 H, *p*‐C*H* Ph), 6.92 (t, ^3^ *J*_HH_=7.3 Hz, 4 H, *p*‐C*H* Ph), 6.84–6.80 (m, 16 H, *m*‐C*H* Ph), 6.78 (s, 4 H, C*H* Mes), 2.80–2.65 (m, 4 H, C*H*_2_), 2.65–2.52 (m, 4 H, C*H*_2_), 2.24 (s, 6 H, C*H*_3_), 1.93 (s, 12 H, C*H*_3_)  ppm. ^13^C{^1^H} NMR (C_6_D_6_, 125.8 MHz): *δ*=146.9 (B*C*_q_, identified by HMBC) 140.8 (s, *C*_q_ Mes), 138.6–138.2 (m, P*C*_q_), 137.3–136.9 (m, P*C*_q_), 135.8 (s, *C*_q_ Mes), 134.3 (m, *o‐C*H Ph), 134.2 (m, *o‐C*H Ph), 129.5 (m, *p‐C*H Ph), 128.7 (s, *p‐C*H Ph), 128.7 (m, *m‐C*H Ph), 128.5 (s, *C*H Mes), 127.3 (m, *m‐C*H), 30.4–30.1 (m, P*C*H_2_), 23.1 (s, *C*H_3_ Mes), 21.3 (s, *C*H_3_ Mes) ppm. ^11^B NMR (C_6_D_6_, 160.5 MHz): not detected. ^31^P{^1^H} NMR (C_6_D_6_, 202.5 MHz): *δ*=32.4 (s, ^1^ *J*_WP_=288 Hz) ppm. Elemental analysis for [C_70_H_70_BBrN_2_P_4_W] (M_W_=1338.79): calcd (%) C 62.85, H 5.27, N 2.09; found (%) C 63.17, H 5.27, N 1.92. IR (solid): *ṽ*(NN)=1601 cm^−1^. UV/Vis (benzene): *λ*_max_=315, 401 (shoulder) nm.

*Synthesis of **2 j***: Compound **2 b** (27 mg, 20 μmol) was suspended in benzene (1 mL) at ambient temperature and DMAP (2.5 mg, 20 μmol) was added to give a green‐brown solution. The reaction mixture was stirred at room temperature for 20 min. The solution was subsequently treated with pentane (2 mL) and a yellow‐green solid precipitated. After removing the liquid phase, the solid was washed with pentane (3×4 mL), and all volatiles were evaporated at ambient temperature to afford **2 j** in 75 % yield (22 mg, 15 μmol). ^1^H NMR (CD_2_Cl_2_, 400.1 MHz): 7.83 (br s, 8 H, *o*‐C*H* Ph), 7.36–7.32 (m, 6 H, *p*‐C*H* Ph + C*H* DMAP), 7.26 (t, ^3^ *J*_HH_=7.4 Hz, 8 H, *m*‐C*H* Ph), 7.06 (br s, 8 H, *o*‐C*H* Ph), 6.93 (t, ^3^ *J*_HH_=7.0 Hz, 4 H, *p*‐C*H* Ph), 6.87–6.81 (m, 10 H, *m*‐C*H* Ph + C*H* DMAP), 6.55 (s, 2 H, C*H* Mes), 3.29 (s, 6 H, N(C*H*_3_)_2_), 2.89–2.76 (m, 4 H, CH_2_), 2.49–2.34 (m, 4 H, CH_2_), 2.15 (s, 3 H, C*H*_3_), 1.76 (s, 6 H, C*H*_3)_  ppm. ^13^C{^1^H} NMR (CD_2_Cl_2_, 100.6 MHz): *δ*=157.3 (s, *C*_q_ DMAP), 140.5 (s, *C*H DMAP), 140.0 (s, *C*_q_ Mes),137.6 (s, *C*_q_ Mes), 137.7–137.3 (m, P*C*_q_), 137.2–136.8 (m, P*C*_q_), 135.1–135.0 (m, *o‐C*H Ph), 134.3–134.2 (m, *o‐C*H Ph), 136.4 (B*C*_q_, identified by HMBC), 131.0 (s, *p‐C*H Ph), 129.0 (br s, *p‐C*H Ph + *m‐C*H Ph), 127.6 (s, *C*H Mes), 127.5–127.4 (m, *m‐C*H Ph), 108.7 (s, *C*H DMAP), 41.9 (s, N*C*H_3_), 34.2–33.8 (m, P*C*H_2_), 22.8 (s, *C*H_3_ Mes), 21.3 (s, *C*H_3_ Mes) ppm. ^11^B NMR (C_6_D_6_, 128.4 MHz): not detected. ^31^P{^1^H} NMR (C_6_D_6_, 162.0 MHz): *δ*=36.6 (s, ^1^ *J*_WP_=287) ppm. Elemental analysis for [C_68_H_69_BBr_2_N_4_P_4_W] (M_W_=1420.68): calcd (%) C 57.49, H 4.90, N 3.94; found (%) C 57.95, H 5.08, N 3.96. IR (solid): *ṽ*(NN)=1511 cm^−1^. UV/Vis (benzene): *λ*_max_=326, 380 nm.

*Synthesis of **2 k***: Compound **2 c** (20 mg, 16 μmol) was suspended in benzene (0.6 mL) at ambient temperature and DMAP (5 mg, 40 μmol) was added. The reaction mixture was heated to 60 °C for 6 h to afford green‐brown crystals. After removing the liquid phase, the crystals were washed with benzene (2×4 mL) and hexane (4 mL). All of the volatiles were evaporated at ambient temperature to afford **2 k** in 94 % yield (22 mg, 15 μmol). The crystals were suitable for X‐ray diffraction experiments. ^1^H NMR (CD_2_Cl_2_, 500.1 MHz): 7.73–7.67 (m, 8 H, *o*‐C*H* Ph), 7.65 (d, ^3^ *J*_HH_=7.8 Hz, 2 H, DMAP(C*H*CN)), 7.42 (t, ^3^ *J*_HH_=7.4 Hz, 4 H, *p*‐C*H* Ph), 7.23 (t, ^3^ *J*_HH_=7.6 Hz, 8 H, *m*‐C*H* Ph), 7.17 (t, ^3^ *J*_HH_=7.4 Hz, 4 H, *p*‐C*H* Ph), 6.97 (t, ^3^ *J*_HH_=7.6 Hz, 8 H, *m*‐C*H* Ph), 6.81–6.76 (m, 8 H, *o*‐C*H* Ph), 6.64 (s, 1 H, C*H* Dur), 6.52 (d, ^3^ *J*_HH_=7.9 Hz, 2 H, DMAP(NC*H*)), 3.28 (s, 6 H, N(C*H*_3_)_2_), 2.93–2.78 (m, 4 H, C*H*_2_), 2.24–2.11 (m, 4 H, C*H*_2_), 1.88 (s, 6 H, C*H*_3_ Dur), 1.41 (s, 6 H, C*H*_3_ Dur) ppm. ^13^C{^1^H} NMR (CD_2_Cl_2_, 125.8 MHz): *δ*=157.5 (s, *C*_q_ DMAP), 142.2 (s, *C*H DMAP), 137.3–137.0 (m, P*C*_q_), 136.0–135.6 (m, P*C*_q_), 135.3 (s, *C*_q_ Dur), 135.3(m, *o‐C*H Ph), 134.1 (m, *o‐C*H Ph), 133.7 (s, *C*_q_ Dur), 132.6 (B*C*_q_, identified by HMBC), 132.3 (s, *C*H Dur), 130.6 (s, *C*H *p‐C*H Ph), 129.5 (s, *C*H *p‐C*H Ph), 128.6 (m, *m‐C*H Ph), 127.5 (m, *m‐C*H Ph), 107.4 (s, *C*H DMAP), 40.9 (s, N*C*H_3_), 34.4–34.1 (m, *C*H_2_), 19.5 (s, *C*H_3_ Dur), 19.4 (s, *C*H_3_ Dur) ppm. ^11^B{^1^H} NMR (CD_2_Cl_2_, 160.5 MHz): *δ*=24.4 ppm. ^31^P{^1^H} NMR (CD_2_Cl_2_, 202.5 MHz): *δ*=39.71 (s, ^1^ *J*_WP_=286) ppm. Elemental analysis for [C_69_H_71_BBr_2_N_4_P_4_W] (M_W_=1434.71): calcd (%) C 57.76, H 4.99 N 3.91; found (%) C 57.63, H 5.00, N 4.03. IR (solid): *ṽ*(NN)=1478 cm^−1^. UV/Vis (CH_2_Cl_2_): *λ*_max_=314 (shoulder), 379 nm.

*Synthesis of **2 l***: Compound **2 b** (30 mg, 23 μmol) was suspended in toluene (1.6 mL) at ambient temperature and MeOTf (9 mg, 60 μmol) was added. Purple crystals were obtained after stirring for 2 d at room temperature. After removing the liquid phase, the crystals were washed with benzene (2×4 mL) and hexane (4 mL). All of the volatiles were evaporated at ambient temperature to afford **2 l** in 91 % yield (32 mg, 21 μmol). The crystals were suitable for X‐ray diffraction experiments. ^1^H NMR (CD_2_Cl_2_, 500.1 MHz): *δ*=7.63–7.57 (m, 8 H, *o*‐C*H* Ph), 7.56 (t, ^3^ *J*_HH_=7.3 Hz, 4 H, *p*‐C*H* Ph), 7.43 (t, ^3^ *J*_HH_=7.5 Hz, 8 H, *m*‐C*H* Ph), 7.29 (t, ^3^ *J*_HH_=7.4 Hz, 4 H, *p*‐C*H* Ph), 7.07 (t, ^3^ *J*_HH_=7.5 Hz, 8 H, *m*‐C*H* Ph), 6.92 (br s, 8 H, *o*‐C*H* Ph), 6.77 (s, 2 H, C*H* Mes), 3.16–2.99 (m, 4 H, C*H*_2_), 2.99–2.84 (m, 4 H, C*H*_2_), 2.22 (s, 3 H, C*H*_3_ Mes), 2.17 (s, 6 H, C*H*_3_ Mes), 1.56 (s, 3 H, NC*H*_3_)  ppm. ^13^C{^1^H} NMR (CD_2_Cl_2_, 125.8 MHz): *δ*=141.2 (s, *C*_q_ Mes), 139.5 (s, *C*_q_ Mes), 135.0–134.7 (m, P*C*_q_), 133.9–133.6 (m, P*C*_q_), 133.6 (m, *o‐C*H Ph), 133.5 (m, *o‐C*H Ph), 132.0 (m, *p‐C*H Ph), 130.7 (m, *p‐C*H Ph), 129.9 (m, *m‐C*H Ph), 128.4 (m, *m‐C*H Ph), 127.9 (s, *C*H Mes), 126.9 (B*C*_q_, identified by HMBC), 42.9 (s, N*C*H_3_), 31.5–31.2 (m, P*C*H_2_), 23.7 (s, *C*H_3_ Mes), 21.3 (s, *C*H_3_ Mes) ppm. ^11^B NMR (CD_2_Cl_2_, 160.5 MHz): not detected. ^31^P{^1^H} NMR (CD_2_Cl_2_, 202.5 MHz, 233 K): *δ*=25.2 (*J*_PP_=141 Hz, ^1^ *J*_WP_=282 Hz), 15.3 (*J*_PP_=141 Hz, ^1^ *J*_WP_=274 Hz) ppm. ^19^F NMR (CD_2_Cl_2_, 470.6 MHz): *δ*=−73.9 (s), −78.9 (s) ppm. Elemental analysis for [C_64_H_62_BBrF_6_N_2_O_6_P_4_S_2_W] (M_W_=1531.77): calcd (%) C 50.18, H 4.08, N 1.83, S 4.19; found (%) C 49.87, H 4.29, N 1.88, S 3.99. UV/Vis (CH_2_Cl_2_): *λ*_max_=480 (shoulder), 535 nm.

## Conflict of interest

The authors declare no conflict of interest.

## Supporting information

As a service to our authors and readers, this journal provides supporting information supplied by the authors. Such materials are peer reviewed and may be re‐organized for online delivery, but are not copy‐edited or typeset. Technical support issues arising from supporting information (other than missing files) should be addressed to the authors.

Supplementary
