# Supplementary material for: Functionalization of N2 via Formal 1,3‐Haloboration of a Tungsten(0) σ‐Dinitrogen Complex
Source: Chemistry. 2020 Oct 22;26(68):16019–27. doi: 10.1002/chem.202002678 (PMC7756771; doi:10.1002/chem.202002678)
Supplement: Supplementary file 1 — Supplementary [file CHEM-26-16019-s001.pdf]

# Chemistry–A European Journal

Supporting Information


## Functionalization of $N_2$ via Formal 1,3-Haloboration of a Tungsten(0) σ-Dinitrogen Complex


Anna Rempel,[a, b] Soren K. Mellerup,[a, b] Felipe Fantuzzi,[a, b, c] Anselm Herzog,[a, b] Andrea Deißenberger,[a, b] Rüdiger Bertermann,[a, b] Bernd Engels,[c] and Holger Braunschweig*[a, b]

# Characterization Data of all Isolated Compounds (NMR, IR, UV/Vis, CV)

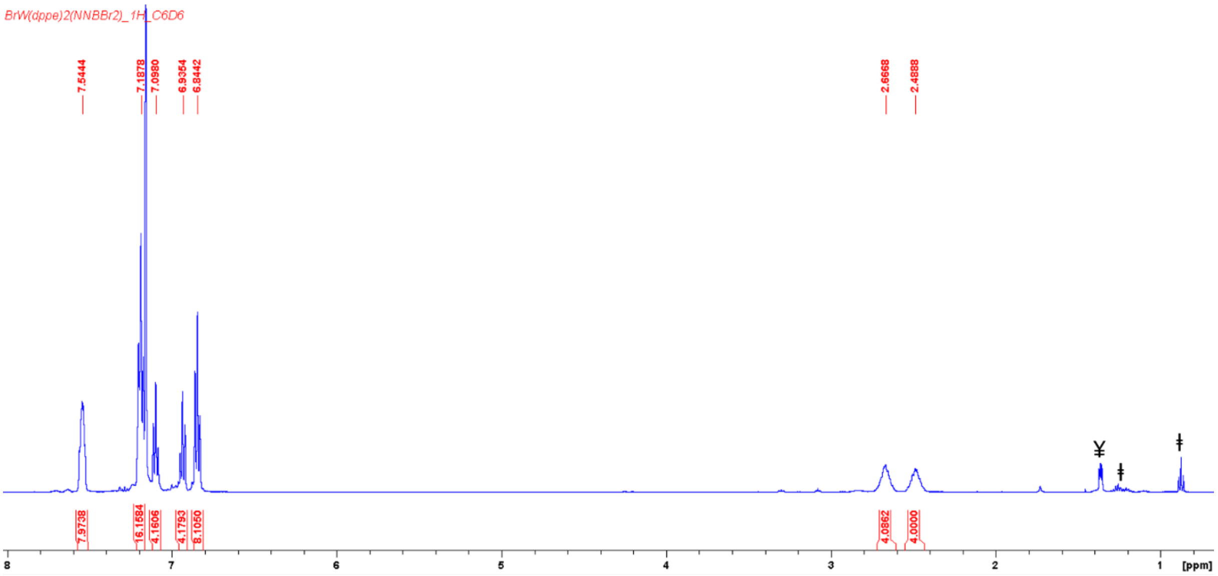


**Figure S1.** $^{1}$H NMR spectrum of **2a** in $C_6D_6$. Marked resonances correspond to residual pentane (ǂ) and $BBr_3SMe_2$ (¥).

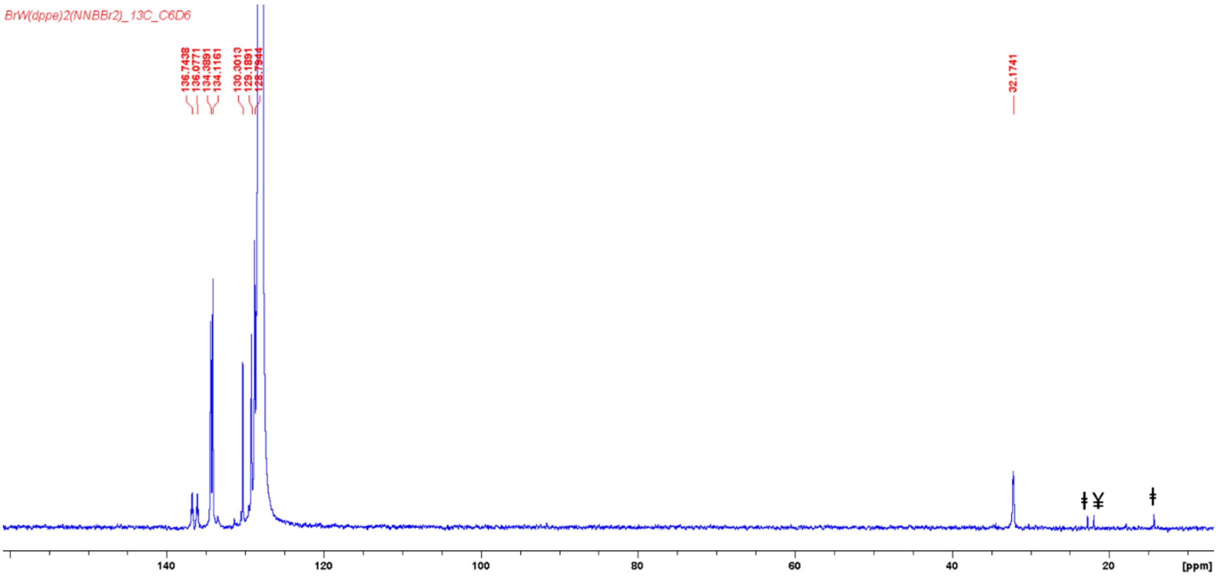


**Figure S2.** $^{13}$C{$^{1}$H} NMR spectrum of **2a** in $C_6D_6$. Marked resonances correspond to residual pentane (ǂ) and $BBr_3SMe_2$ (¥).

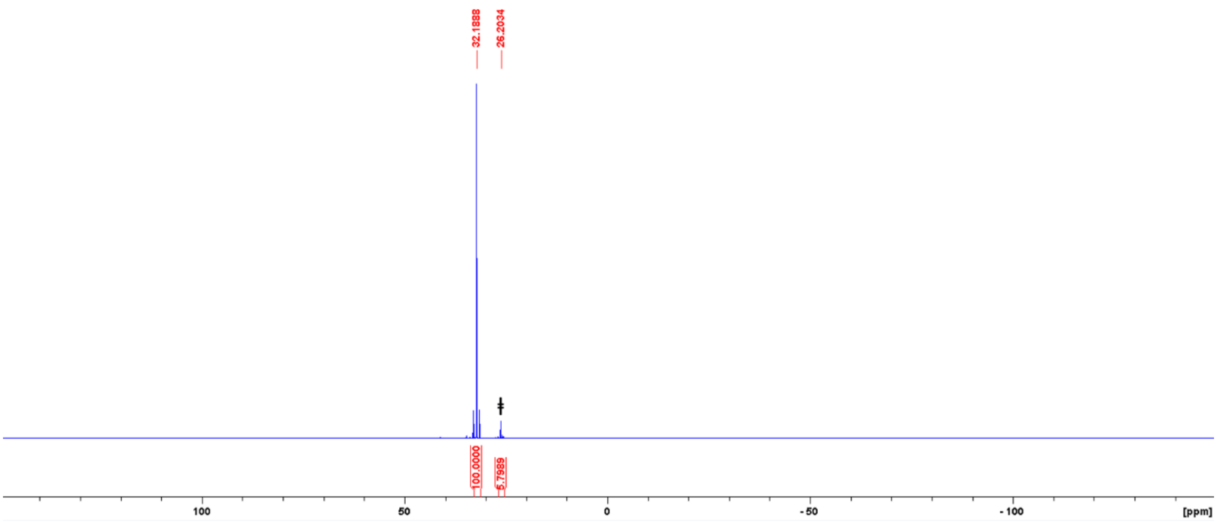


**Figure S3.** $^{31}P\{^1H\}$ NMR spectrum of **2a** in $C_6D_6$. Resonances marked with ǂ correspond to decomposition product **2a'**.

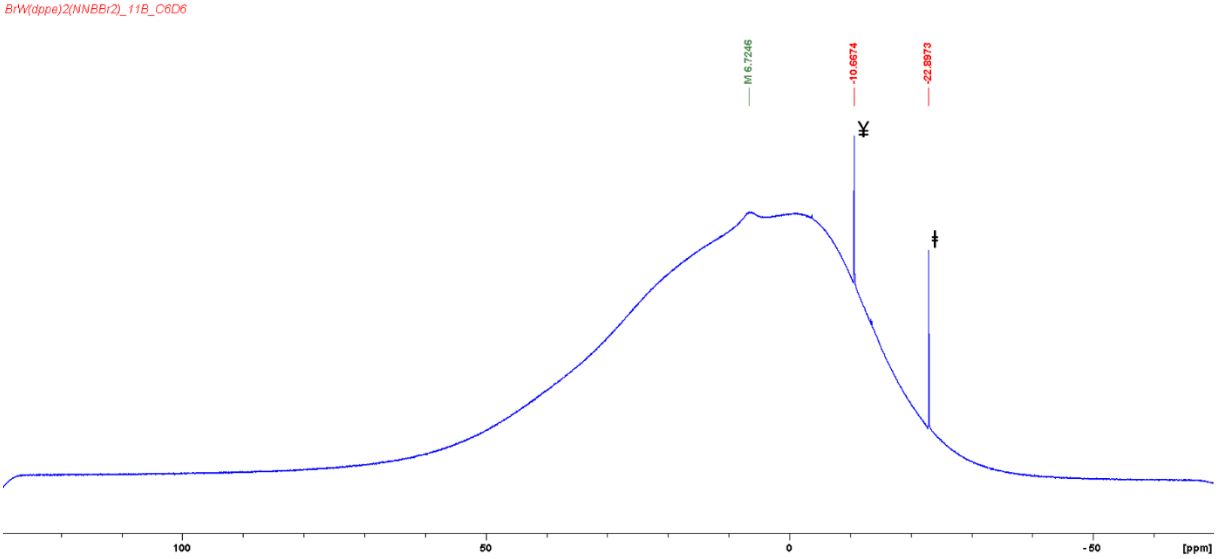


**Figure S4.** $^{11}B$ NMR spectrum of **2a** in $C_6D_6$. Marked resonances correspond to residual $BBr_3{\cdot}SMe2$ (¥) and decomposition product **2a'** (ǂ).

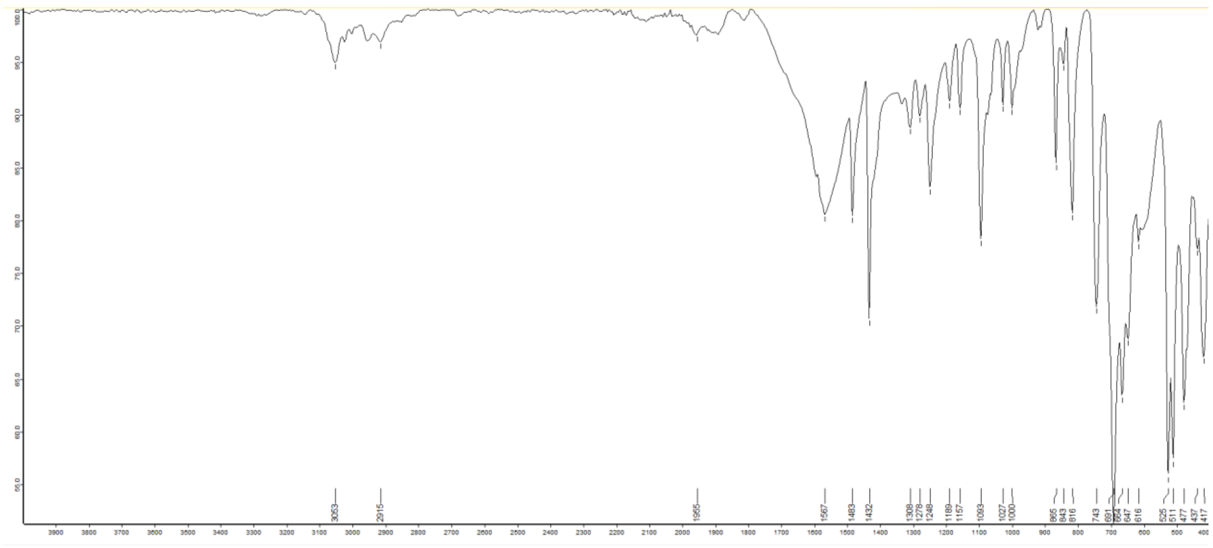

**Figure S5.** Solid-state IR spectrum of **2a**.

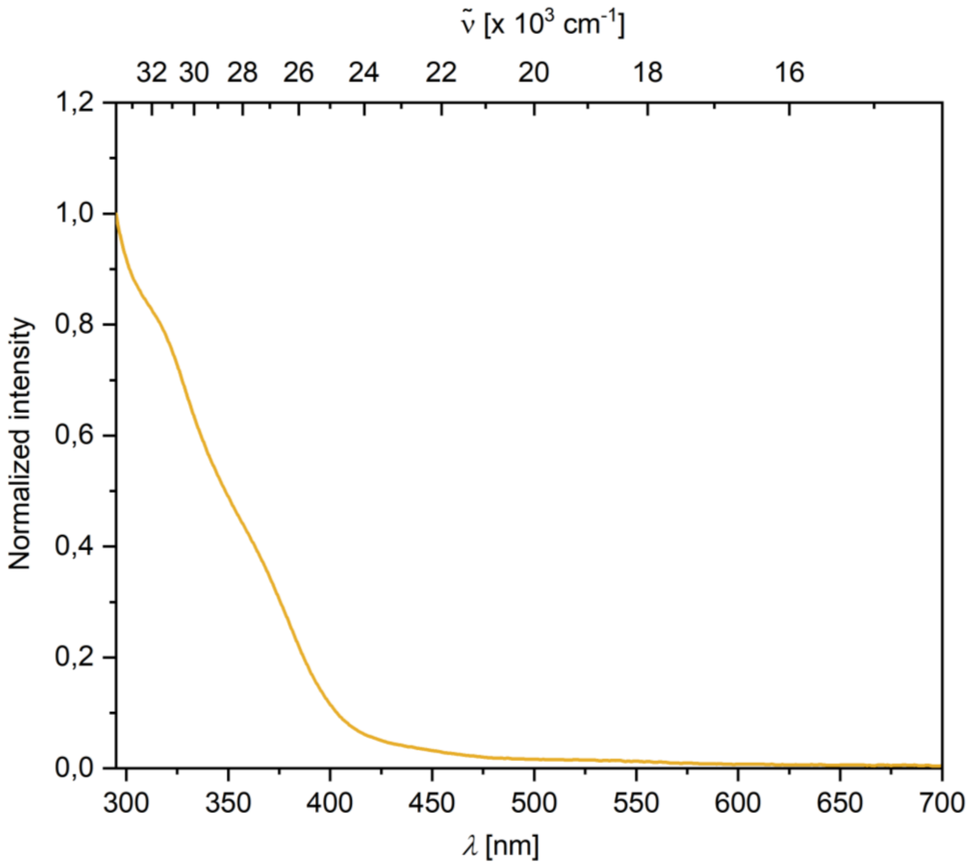


**Figure S6.** UV/Vis spectrum of **2a** in benzene.

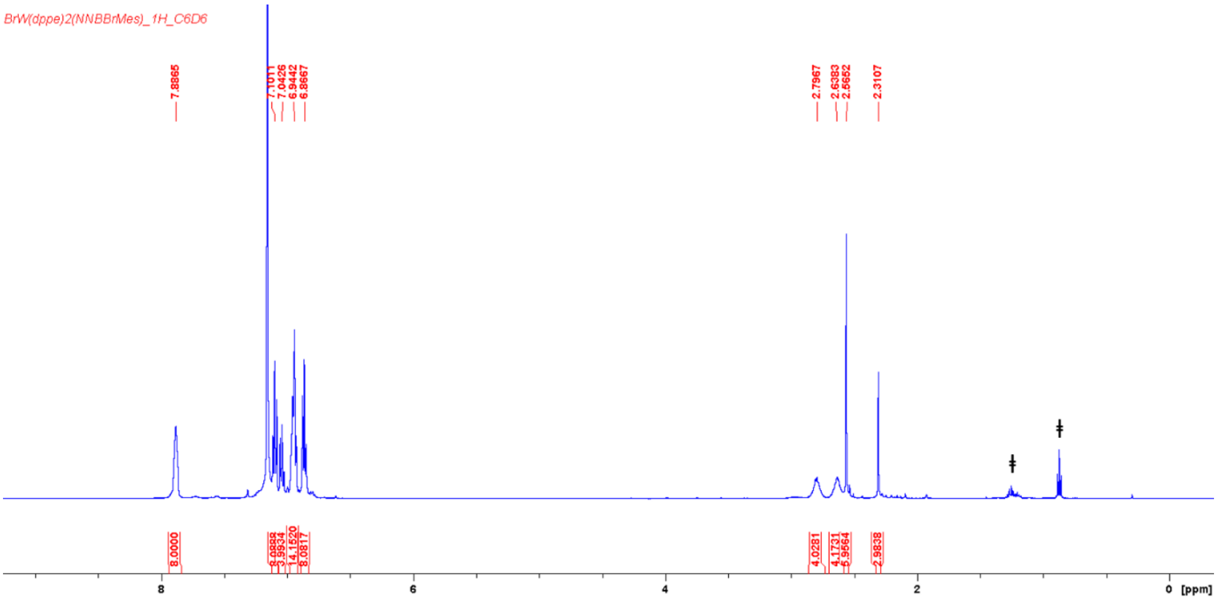


**Figure S7.** $^{1}$H NMR spectrum of **2b** in $C_6D_6$. Resonances marked with ǂ correspond to residual pentane.

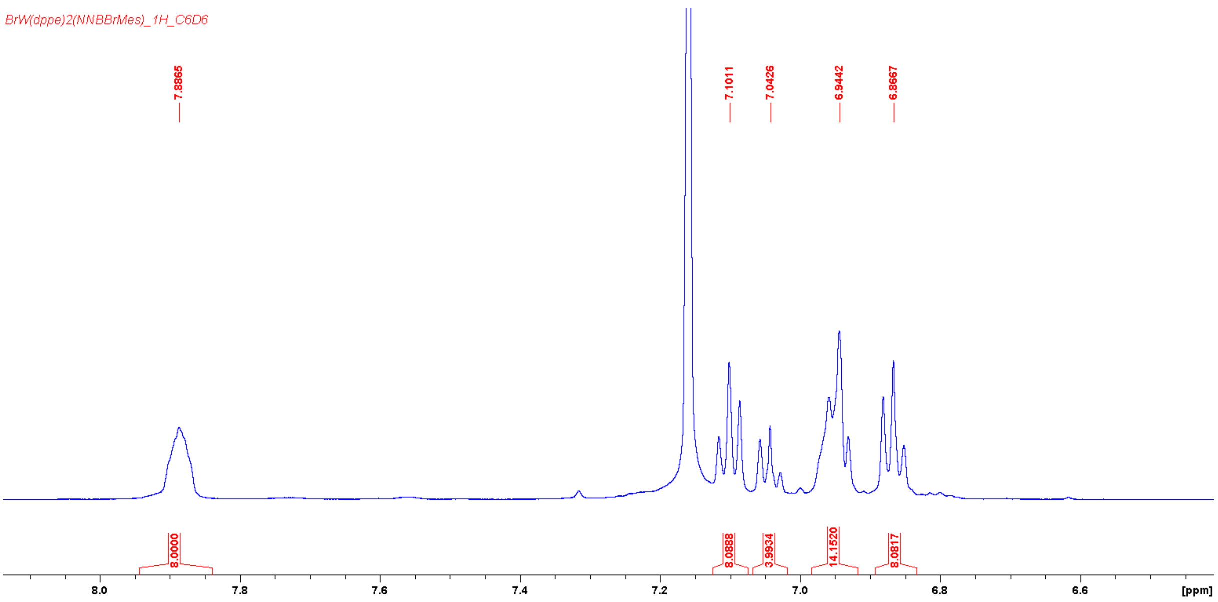


**Figure S8.** Expanded aromatic region of $^1H$ NMR spectrum of **2b** in $C_6D_6$.

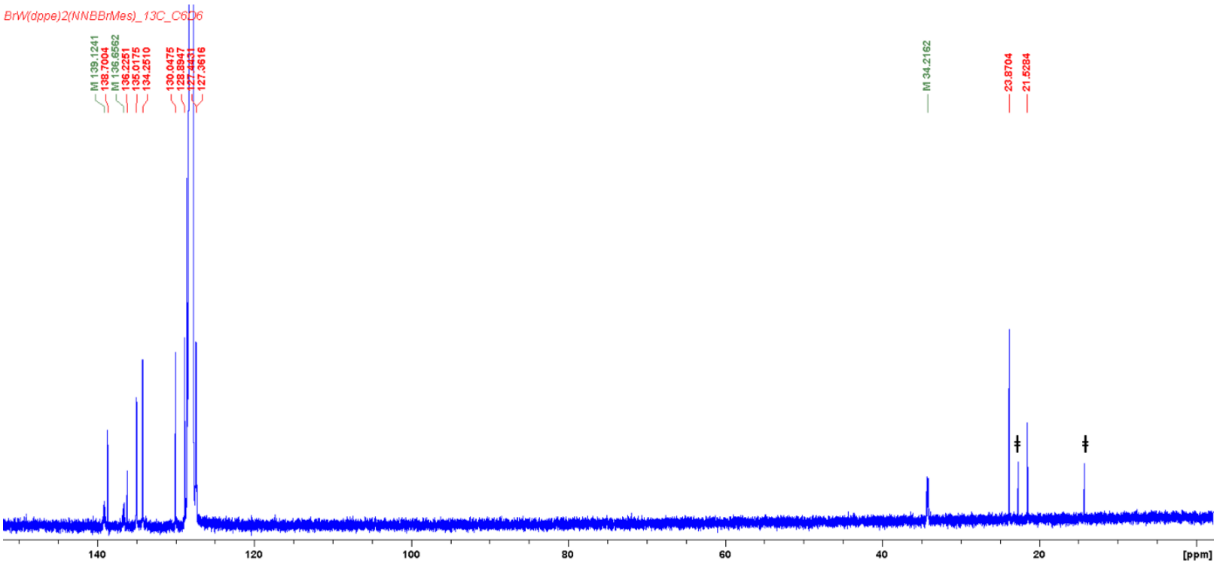


**Figure S9.** $^{13}C\{^1H\}$ NMR spectrum of **2b** in $C_6D_6$. Resonances marked with ǂ correspond to residual pentane.

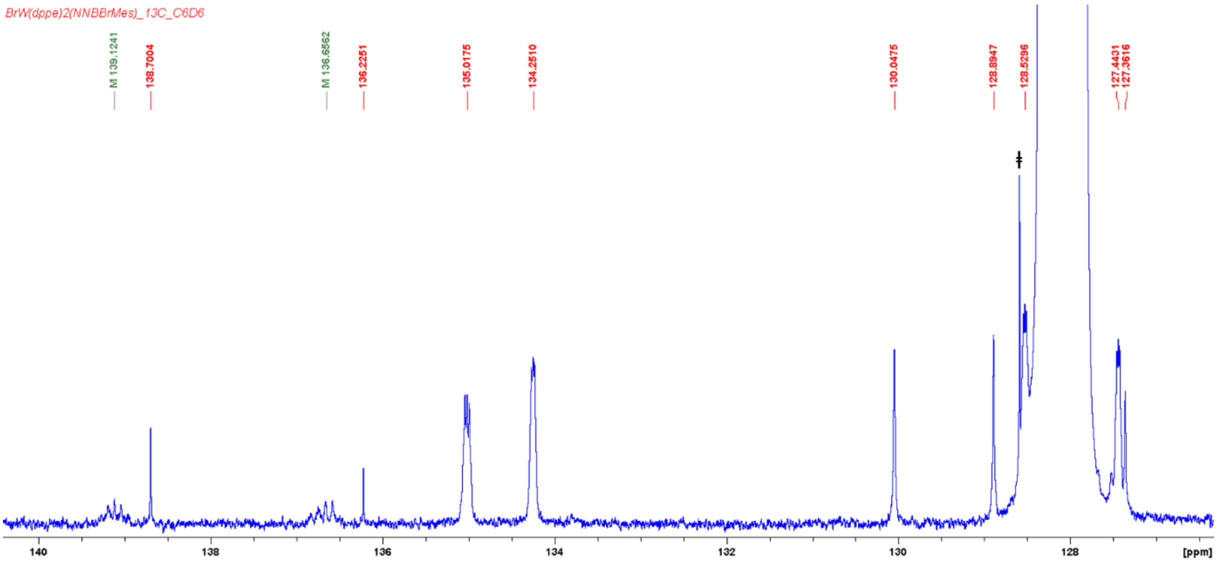


**Figure S10.** Expanded aromatic region of $^{13}C\{^{1}H\}$ NMR spectrum of **2b** in $C_6D_6$. Resonances marked with ǂ correspond to residual benzene.

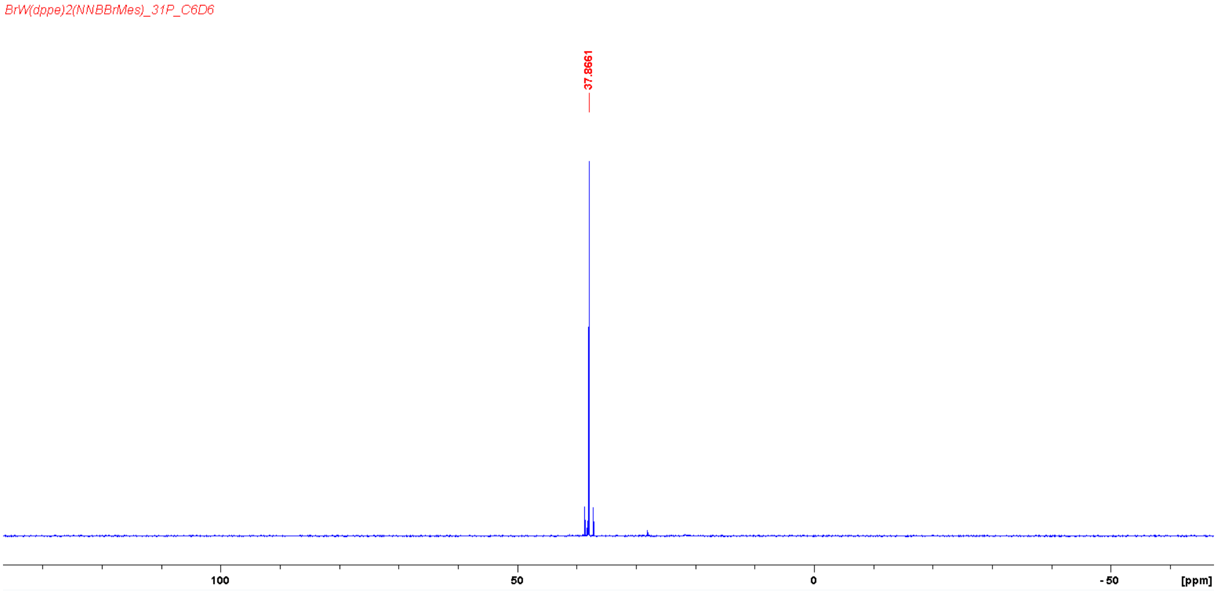


**Figure S11.** $^{13}P\{^{1}H\}$ NMR spectrum of **2b** in $C_6D_6$.

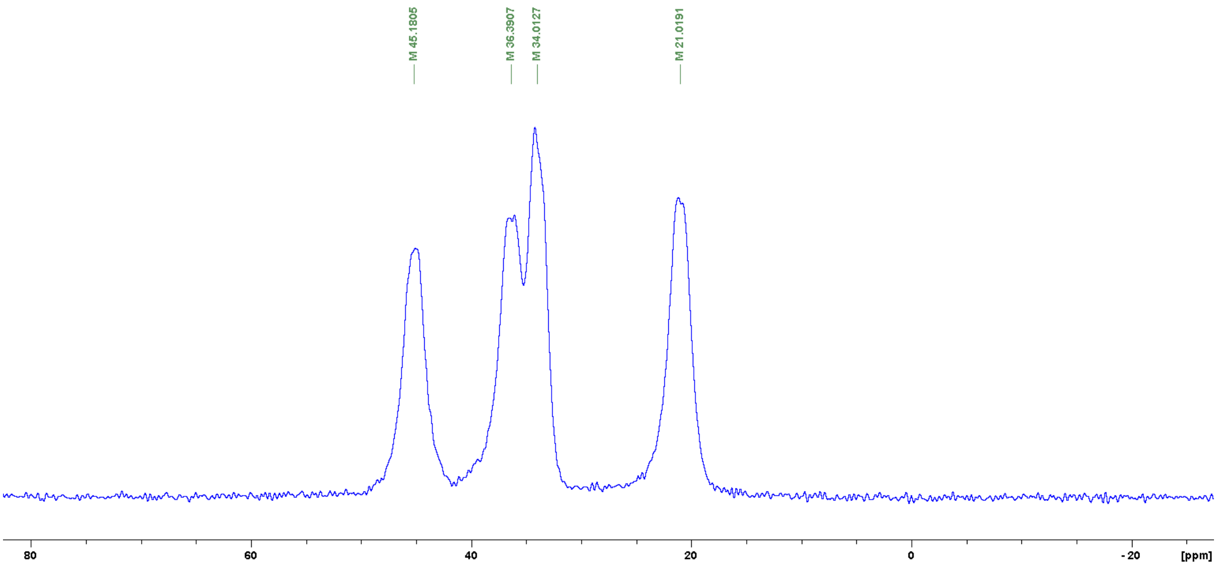


**Figure S12.** Solid-state $^{31}P\{^{1}H\}$ CP/MAS NMR of **2b** at 162.0 MHz.

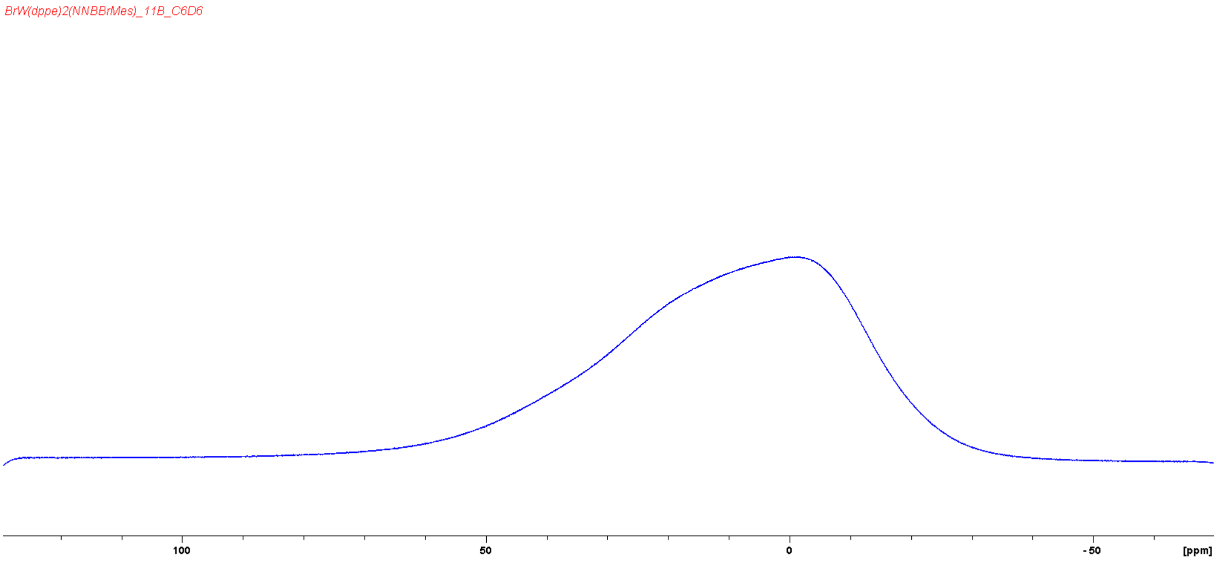


**Figure S13.** $^{11}B$ NMR spectrum of **2b** in $C_6D_6$.

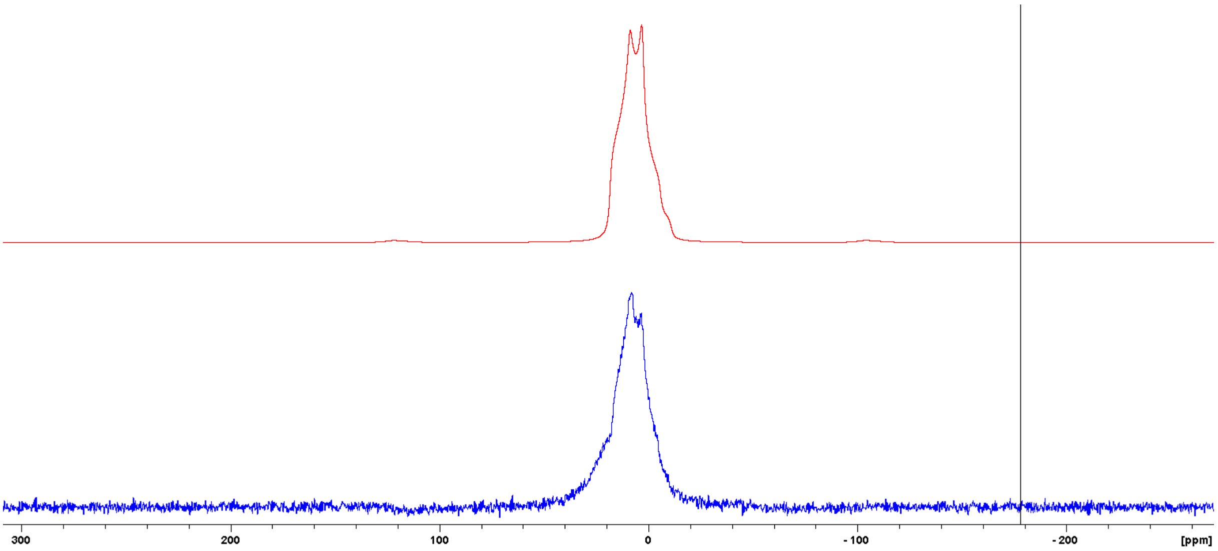


**Figure S14.** Solid-state $^{11}B\{^{1}H\}$ RSHE NMR spectrum of **2b** at 128 MHz. (Top: Simulation) isotropic chemical shift $\delta_{iso}$ = 19.0 ppm, quadrupolar coupling constant $C_Q$ = 2.74 MHz, quadrupolar asymmetry parameter $\eta_{Quad}$ = 0.59.

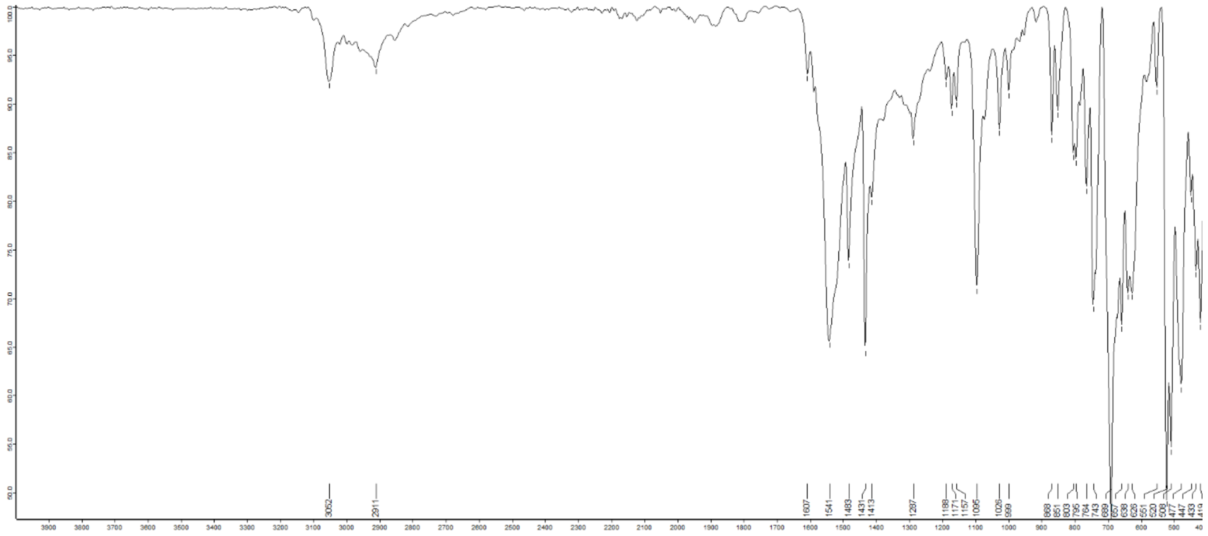

**Figure S15.** Solid-state IR spectrum of **2b**.

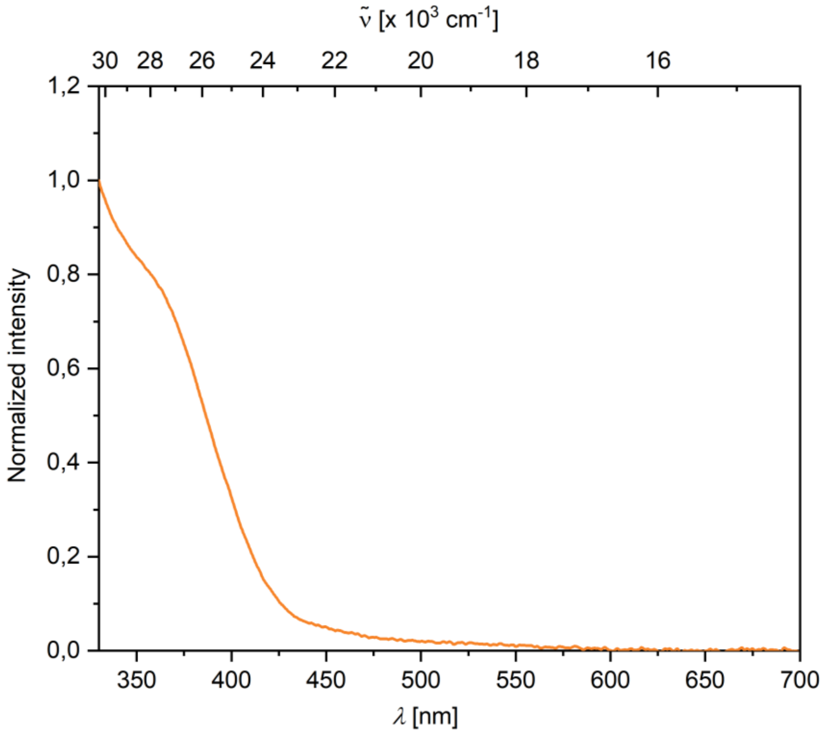


**Figure S16.** UV/Vis spectrum of **2b** in benzene.

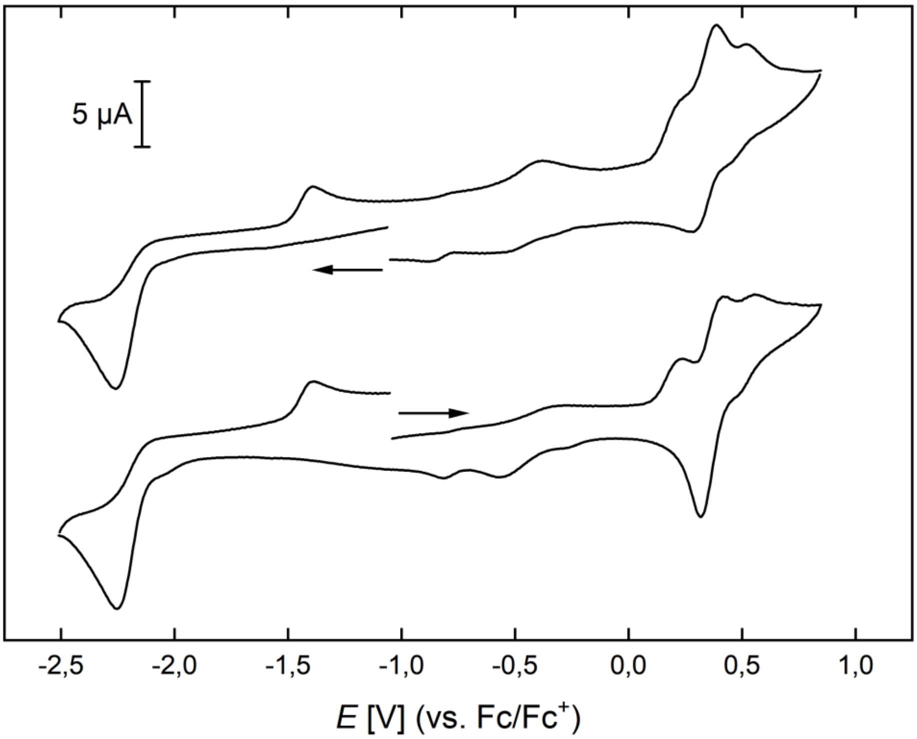


**Figure S17.** Cyclic voltammogram of **2b** in 1,2-difluorobenzene/0.1 M [*n*$Bu_4$N][$PF_6$] measured at 250 mV $s^{-1}$. Formal potentials: $E_{pc}$ = −2.26 V, $E_{pa}$ = +0.18 V, $E_{1/2}$ = +0.35 V, and $E_{pa}$= + 0.52 V (relative to the Fc/$Fc^+$ couple). The arrows indicate the initial scan directions for the cathodic (top) and anodic (bottom) sweeps.

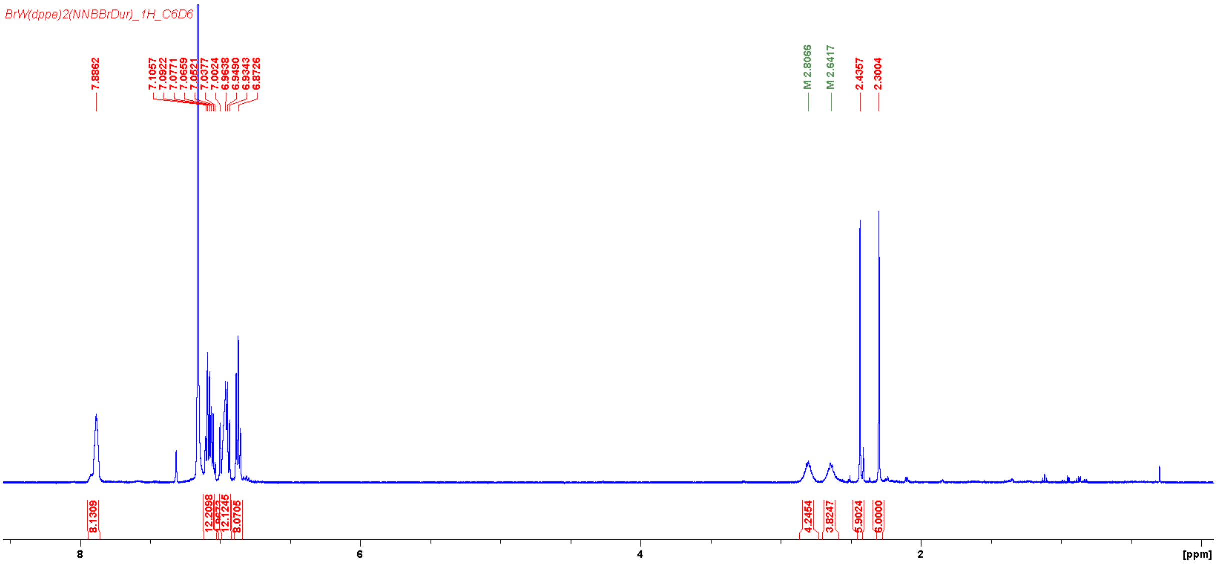


**Figure S18.** $^{1}H$ NMR spectrum of **2c** in $C_6D_6$.

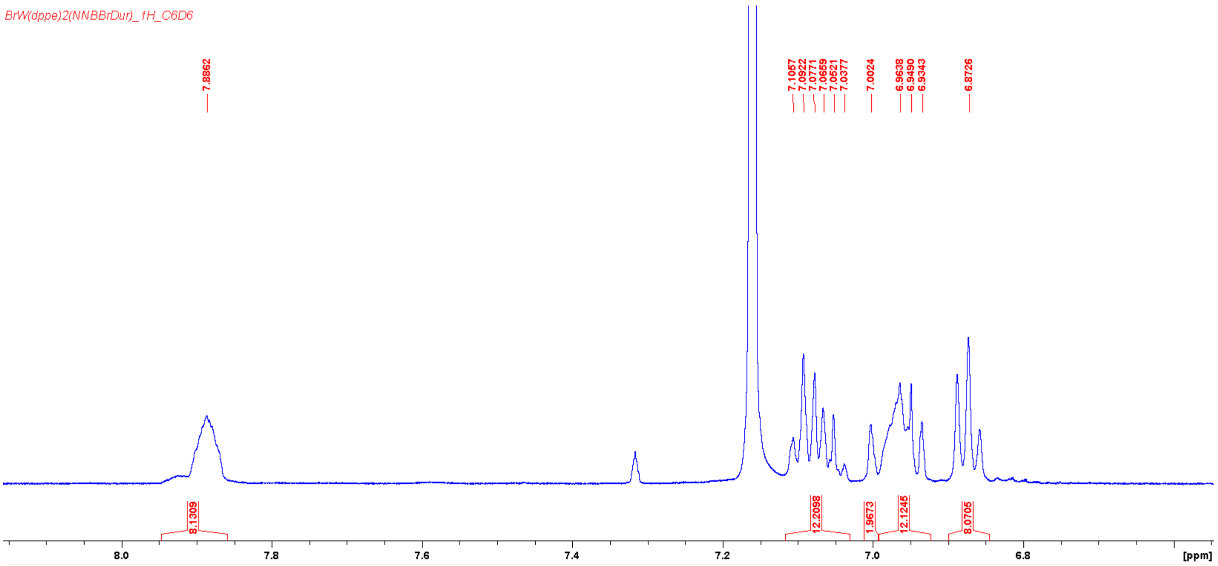


**Figure S19.** Expanded aromatic region of $^{1}H$ NMR spectrum of **2c** in $C_6D_6$.

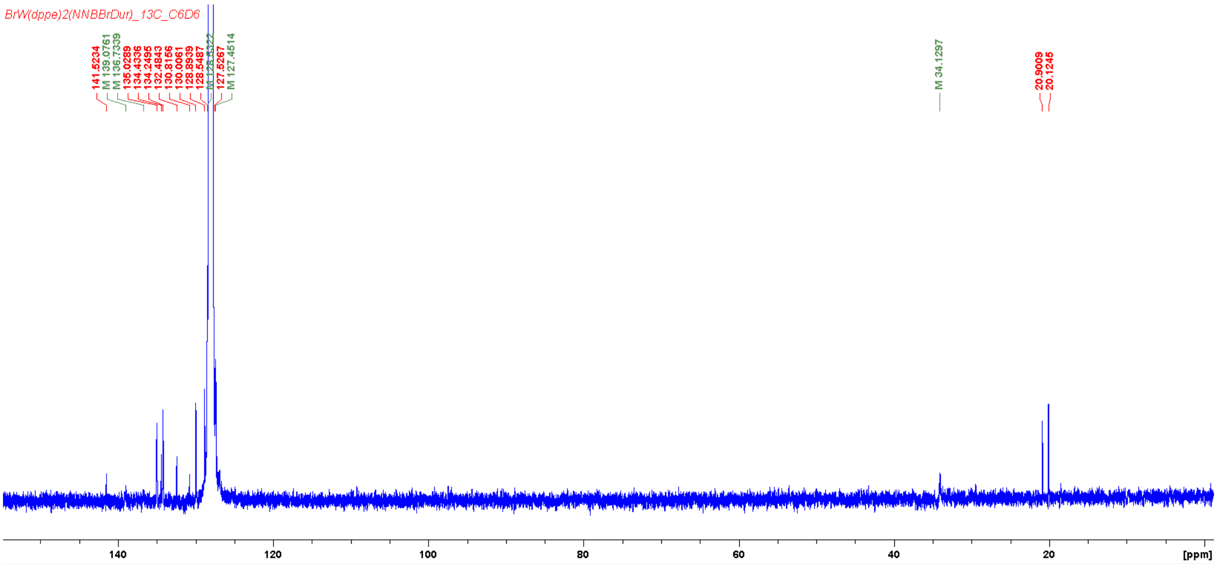


**Figure S20.** $^{13}C\{^{1}H\}$ NMR spectrum of **2c** in $C_6D_6$.

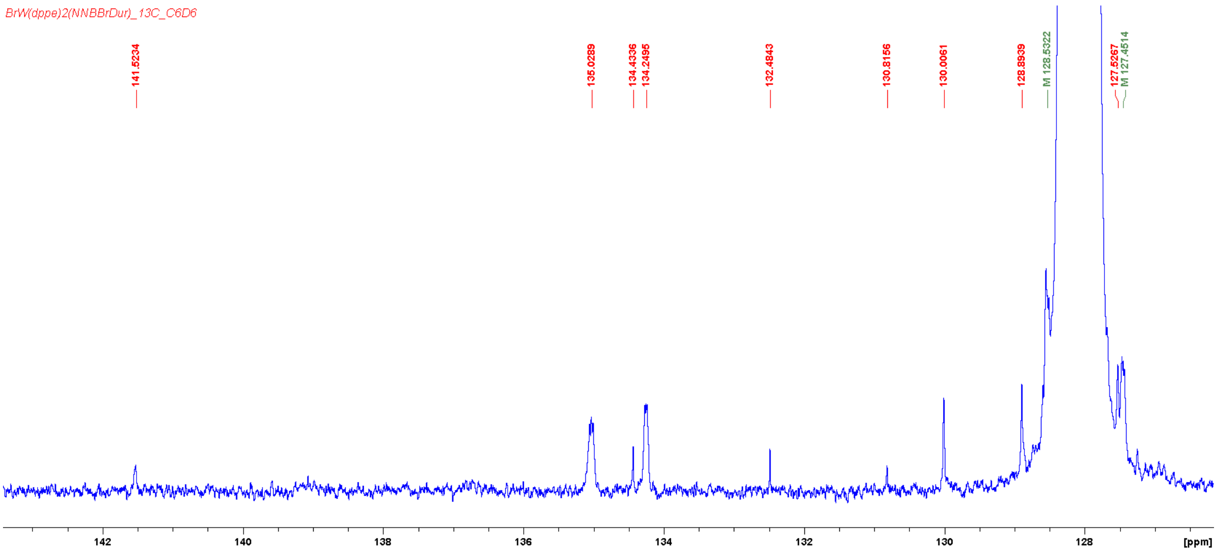


**Figure S21.** Expanded aromatic region of $^{13}C\{^{1}H\}$ NMR spectrum of **2c** in $C_6D_6$.

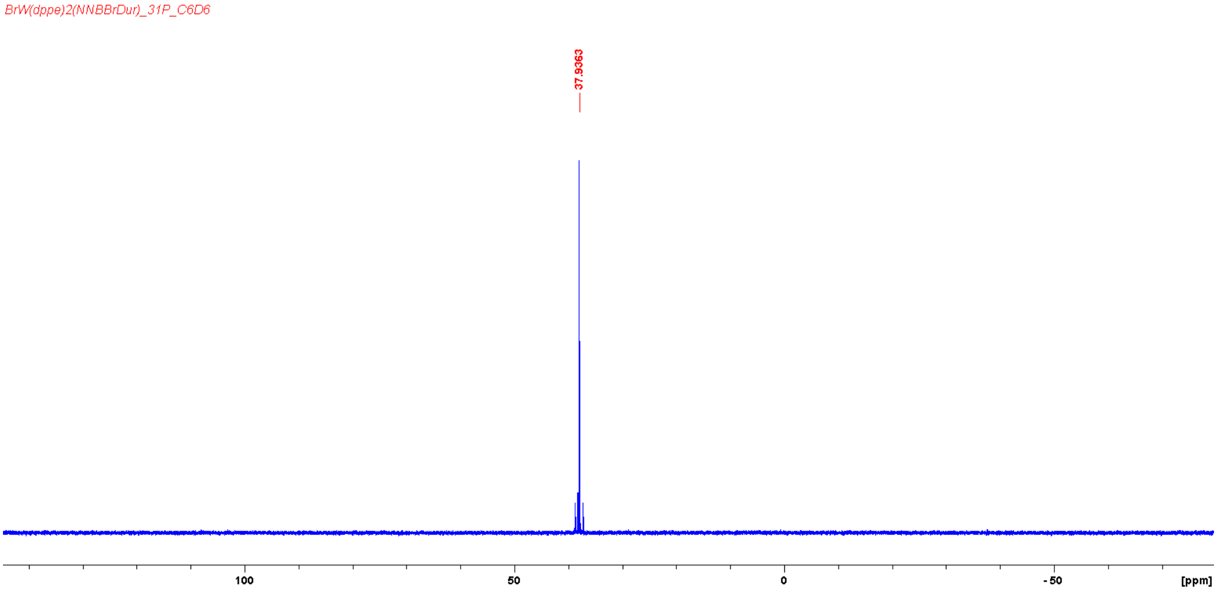


**Figure S22.** $^{31}P\{^{1}H\}$ NMR spectrum of **2c** in $C_6D_6$.

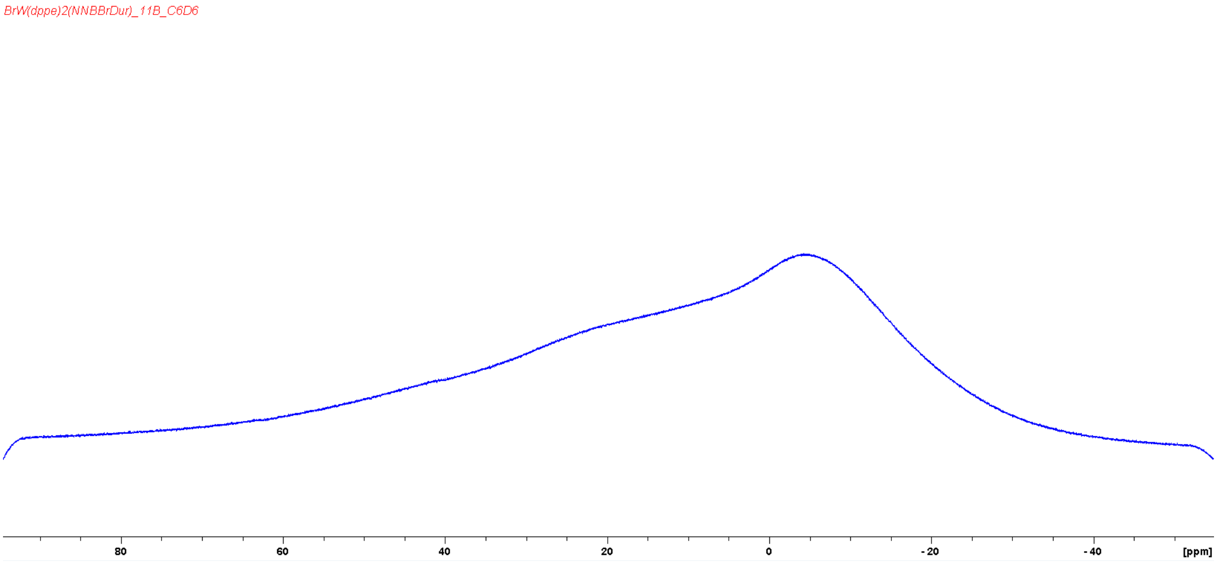


**Figure S23.** $^{11}B$ NMR spectrum of **2c** in $C_6D_6$.

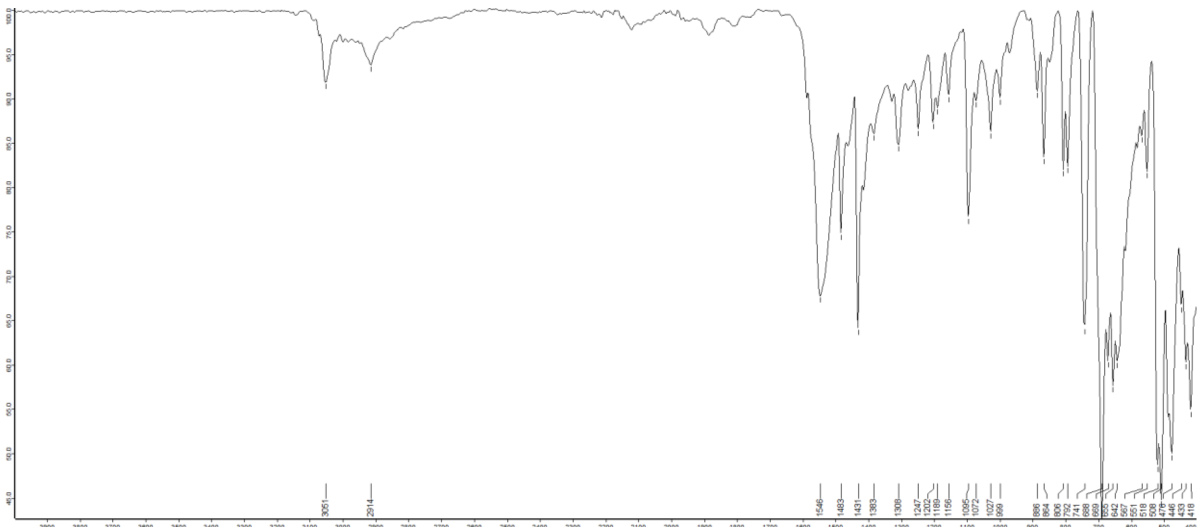

**Figure S24.** Solid-state IR spectrum of **2c**.

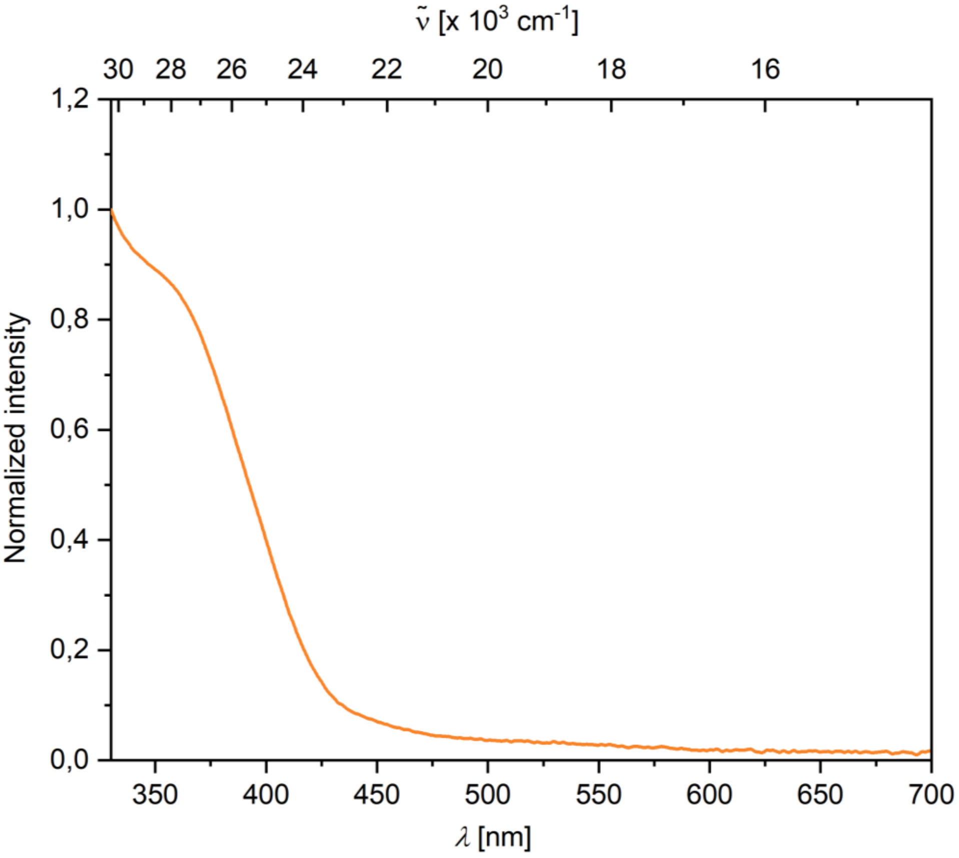


**Figure S25.** UV/Vis spectrum of **2c** in benzene.

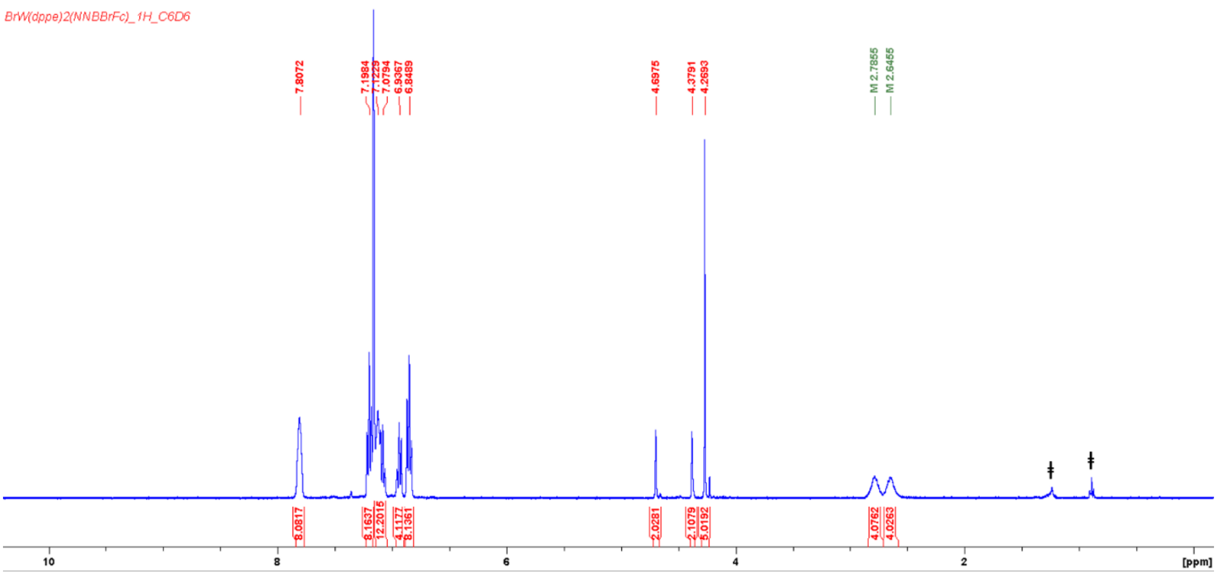


**Figure S26.** $^{1}$H NMR spectrum of **2d** in $C_6D_6$. Resonances marked with ǂ correspond to residual hexane.

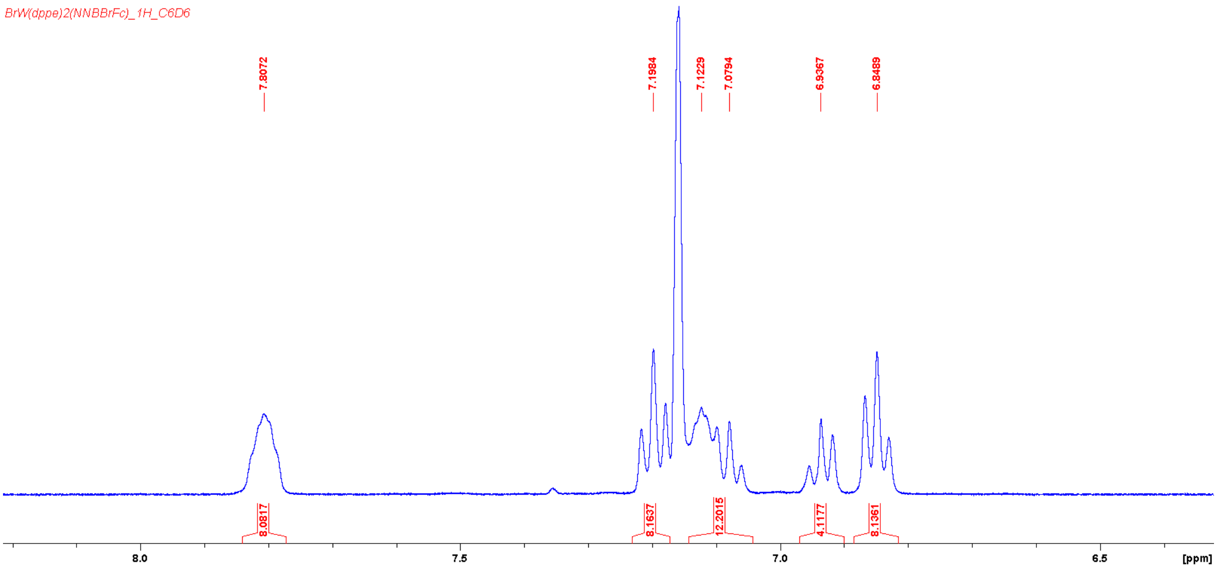


**Figure S27.** Expanded aromatic region of $^{1}$H NMR spectrum of **2d** in $C_6D_6$.

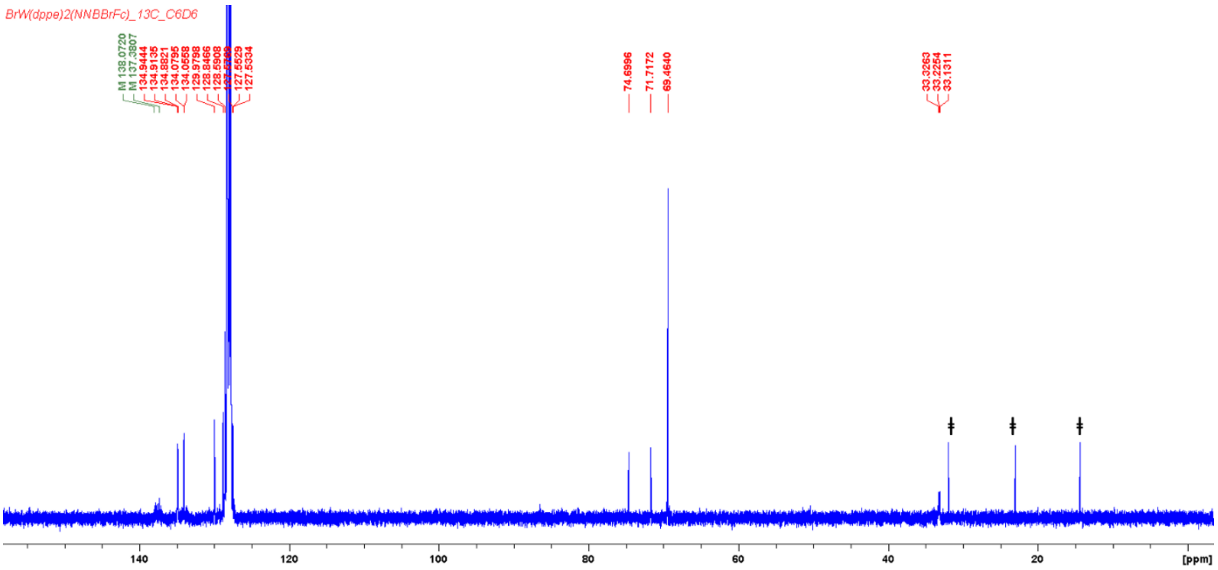


**Figure S28.** $^{13}C\{^{1}H\}$ NMR spectrum of **2d** in $C_6D_6$. Resonances marked with ǂ correspond to residual hexane.

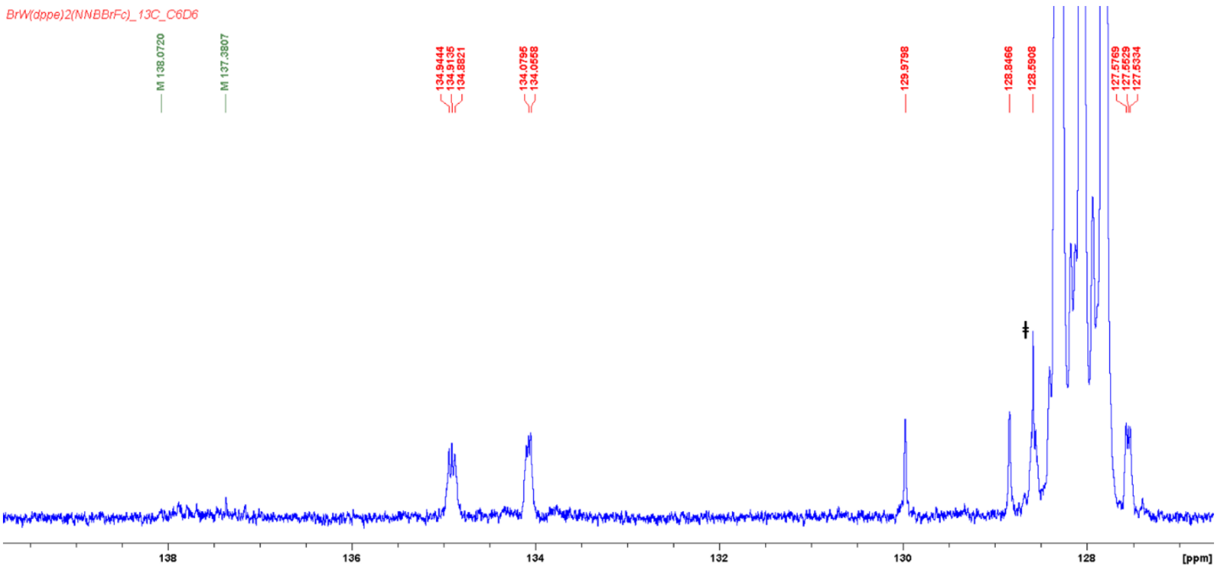


**Figure S29.** Expanded aromatic region of $^{13}C\{^{1}H\}$ NMR spectrum of **2d** in $C_6D_6$. Resonances marked with ǂ correspond to residual benzene.

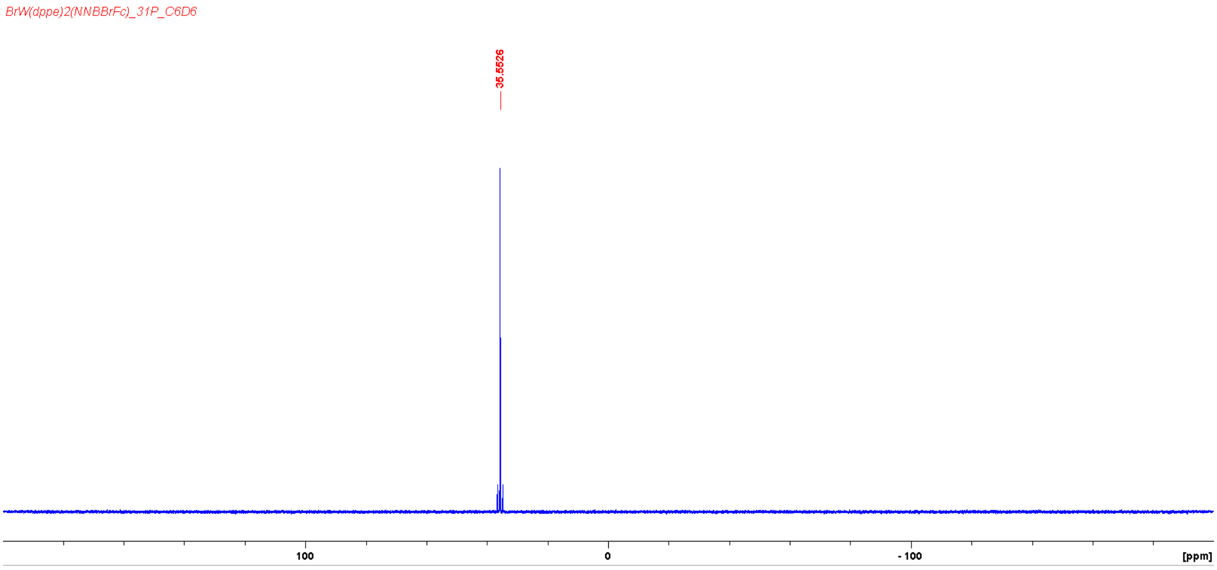


**Figure S30.** $^{31}P\{^{1}H\}$ NMR spectrum of **2d** in $C_6D_6$.

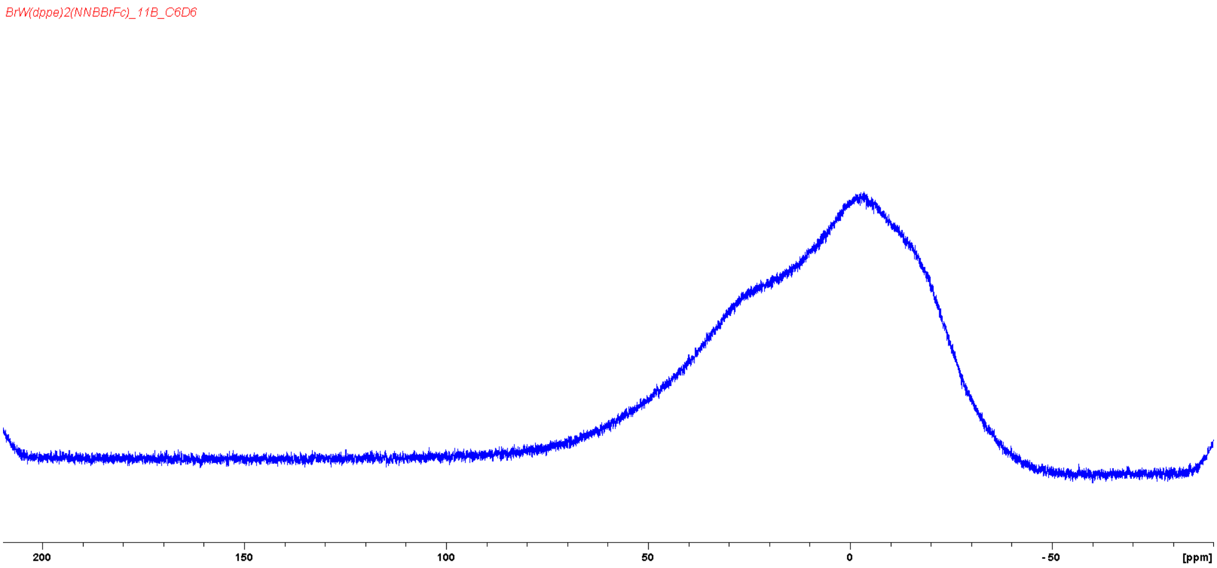


**Figure S31.** $^{11}B$ NMR spectrum of **2d** in $C_6D_6$.

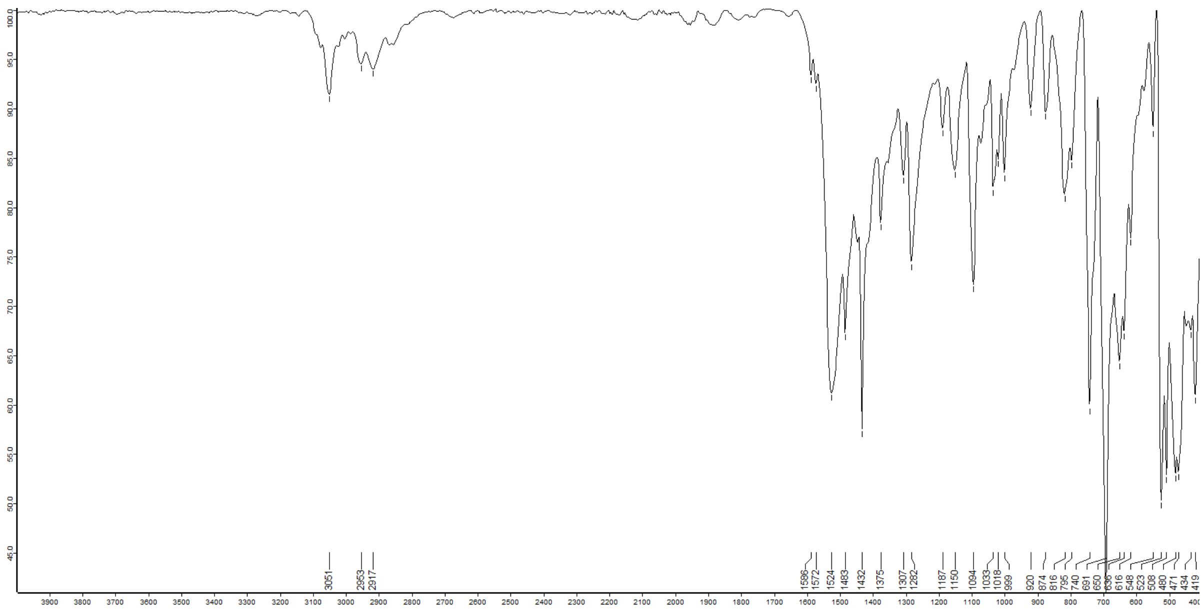

**Figure S32.** Solid-state IR spectrum of **2d**.

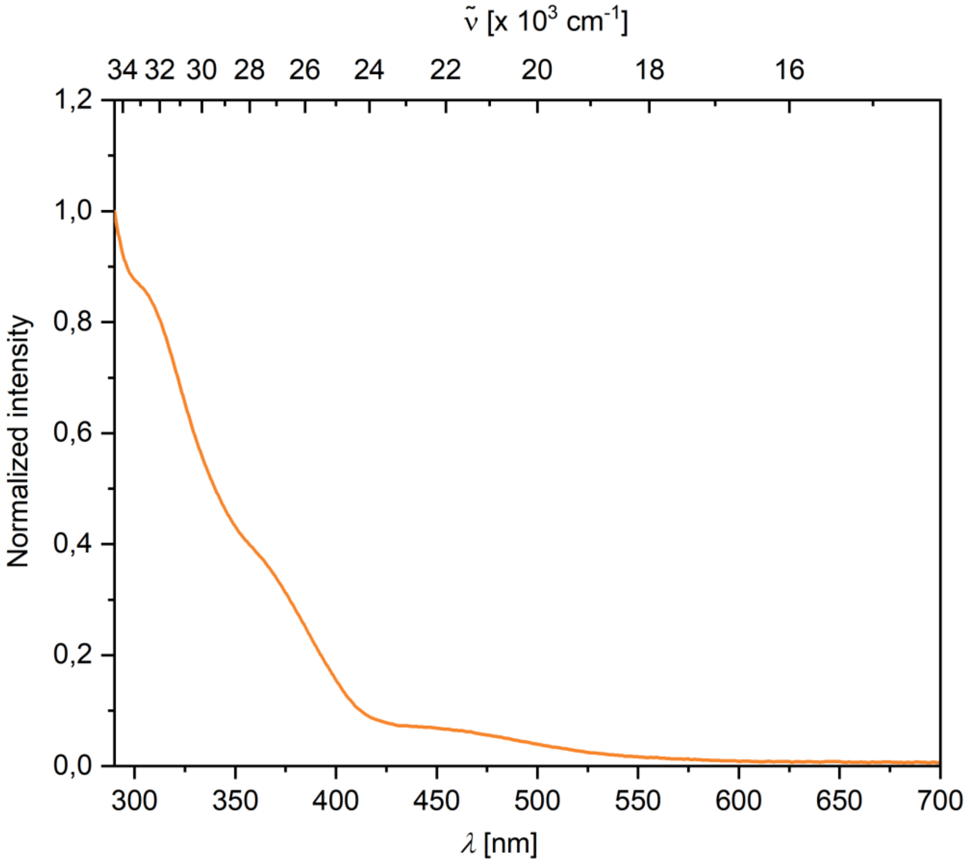


**Figure S33.** UV/Vis spectrum of **2d** in benzene.

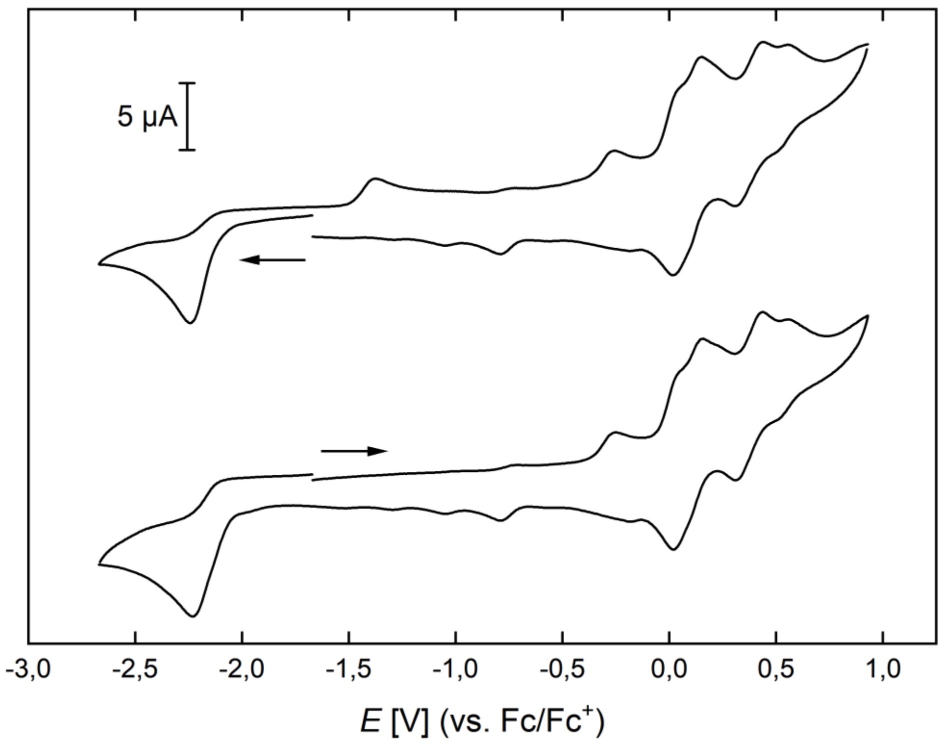


**Figure S34.** Cyclic voltammogram of **2d** in 1,2-difluorobenzene/0.1 M [$n$Bu$_4$N][PF$_6$] measured at 250 mV s$^{-1}$. Formal potentials: $E_{pc}$ = −2.11 V, $E_{pa}$ = −0.14 V, $E_{1/2}$ = +0.22 V (with shoulder at $E_{pa}$ ≈ +0.1 V), $E_{1/2}$ = +0.50 V, $E_{pa}$ = +0.68 V (relative to the Fc/Fc$^+$ couple). The arrows indicate the initial scan directions for the cathodic (top) and anodic (bottom) sweeps.

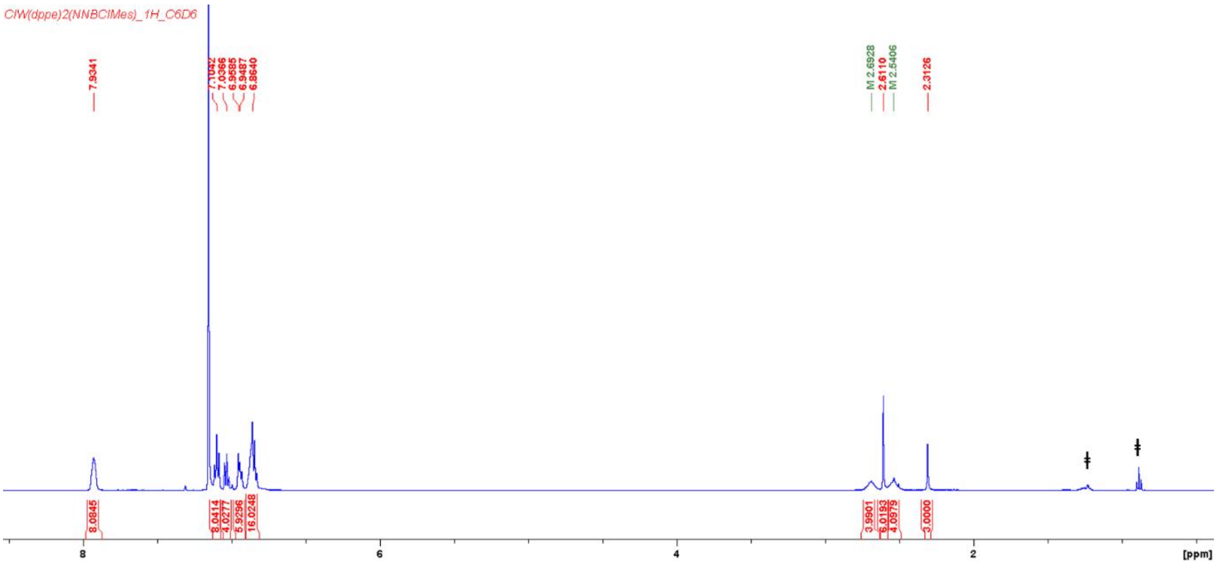


**Figure S35.** $^1$H NMR spectrum of **2e** in $C_6D_6$. Resonances marked with ƚ correspond to residual hexane.

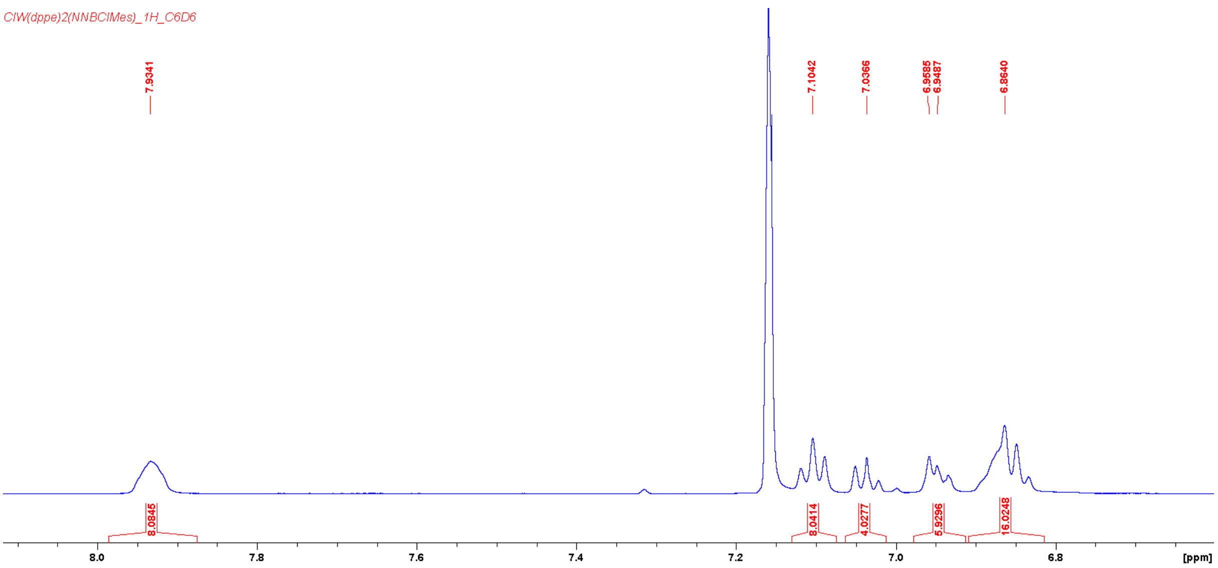


**Figure S36.** Expanded aromatic region of $^{1}$H NMR spectrum of **2e** in $C_6D_6$.

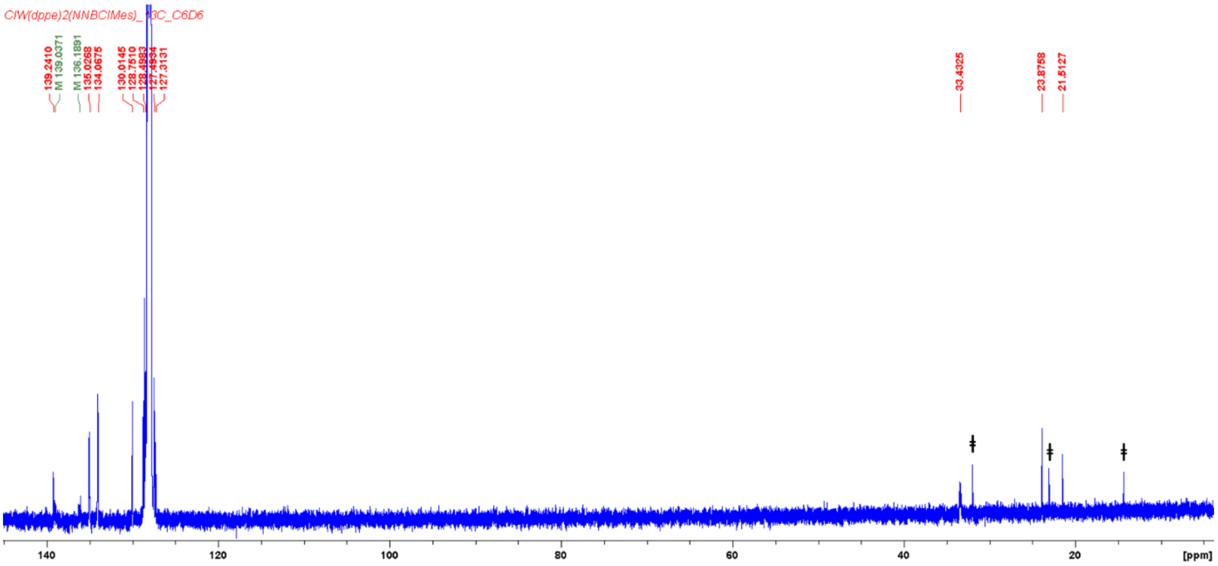


**Figure S37.** $^{13}C\{^1H\}$ NMR spectrum of **2e** in $C_6D_6$. Resonances marked with ǂ correspond to residual hexane.

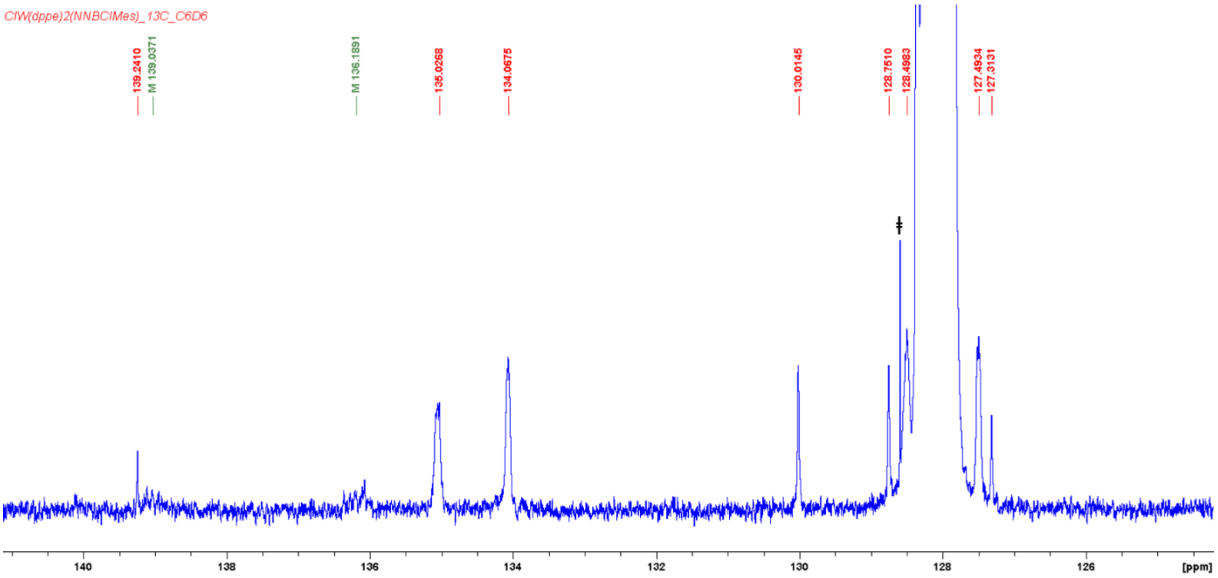


**Figure S38.** Expanded aromatic region of $^{13}C\{^{1}H\}$ NMR spectrum of **2e** in $C_6D_6$. Resonances marked with ǂ correspond to residual benzene.

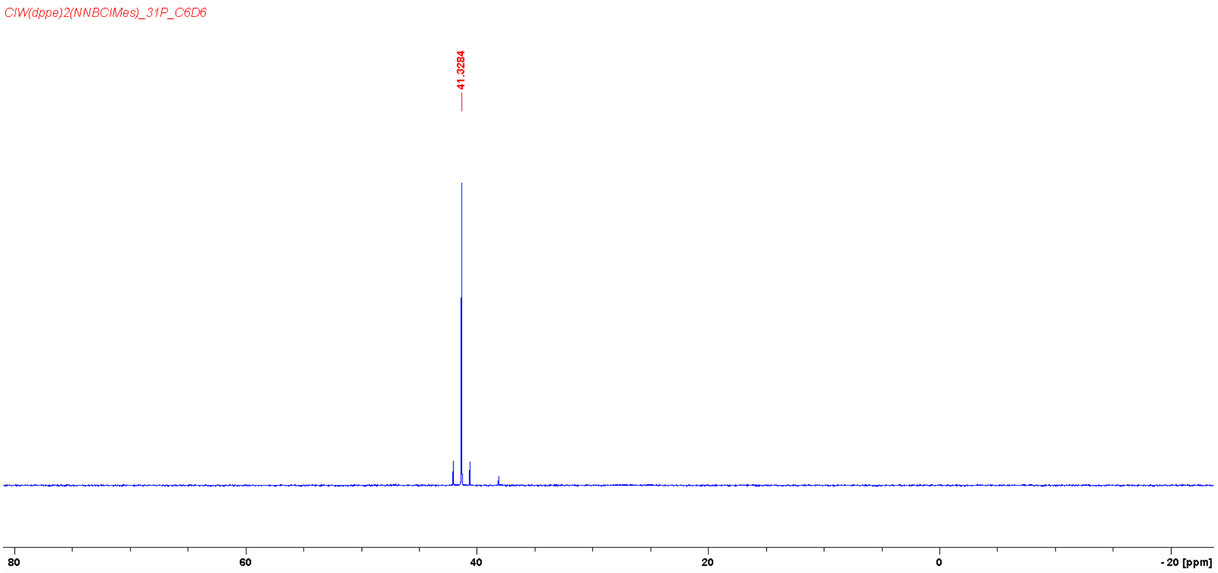


**Figure S39.** $^{31}P\{^{1}H\}$ NMR spectrum of **2e** in $C_6D_6$.

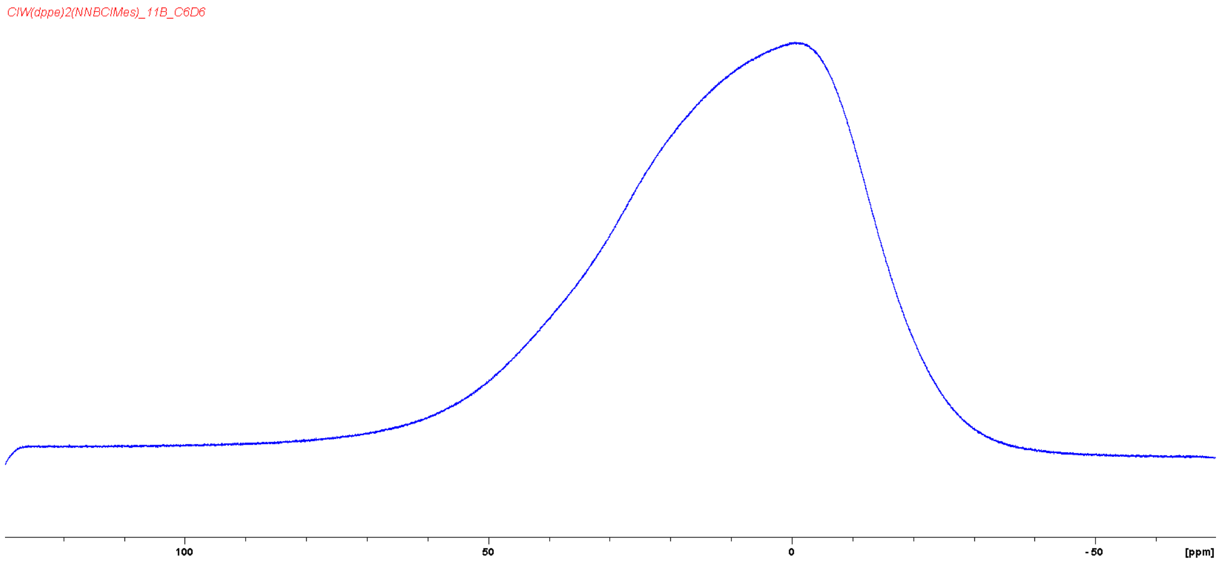


**Figure S40.** $^{11}B$ NMR spectrum of **2e** in $C_6D_6$.

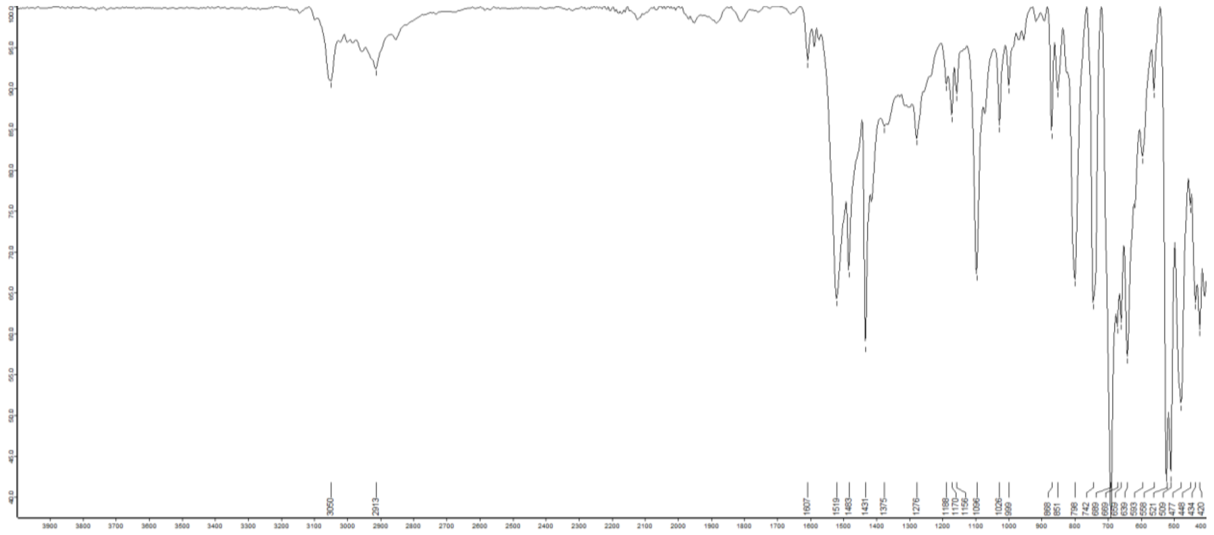

**Figure S41.** Solid-state IR spectrum of **2e**.

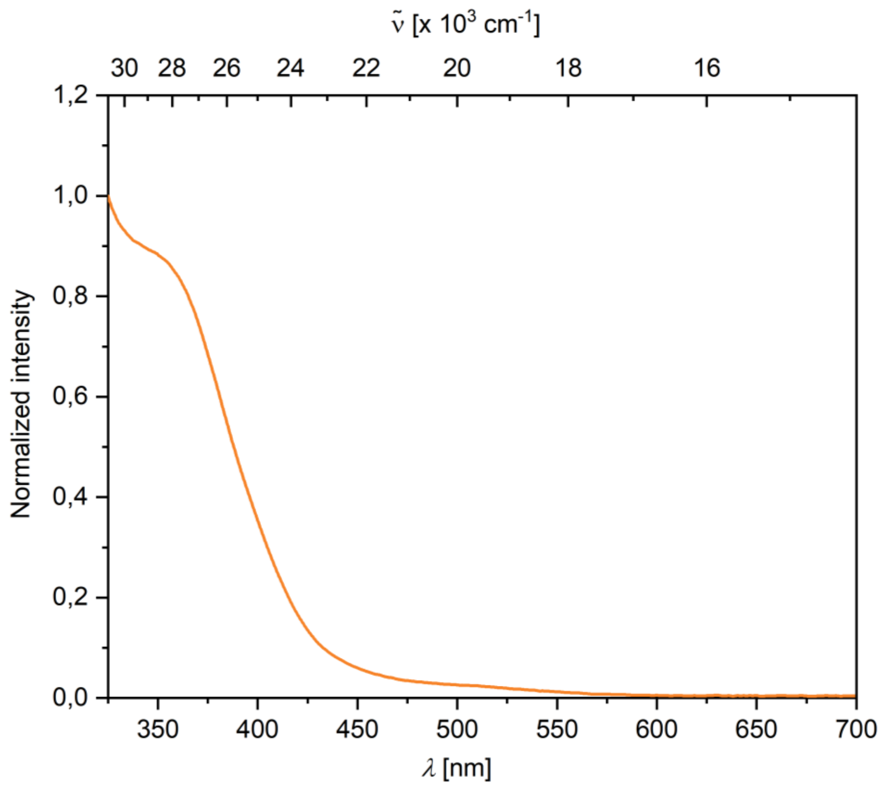


**Figure S42.** UV/Vis spectrum of **2e** in benzene.

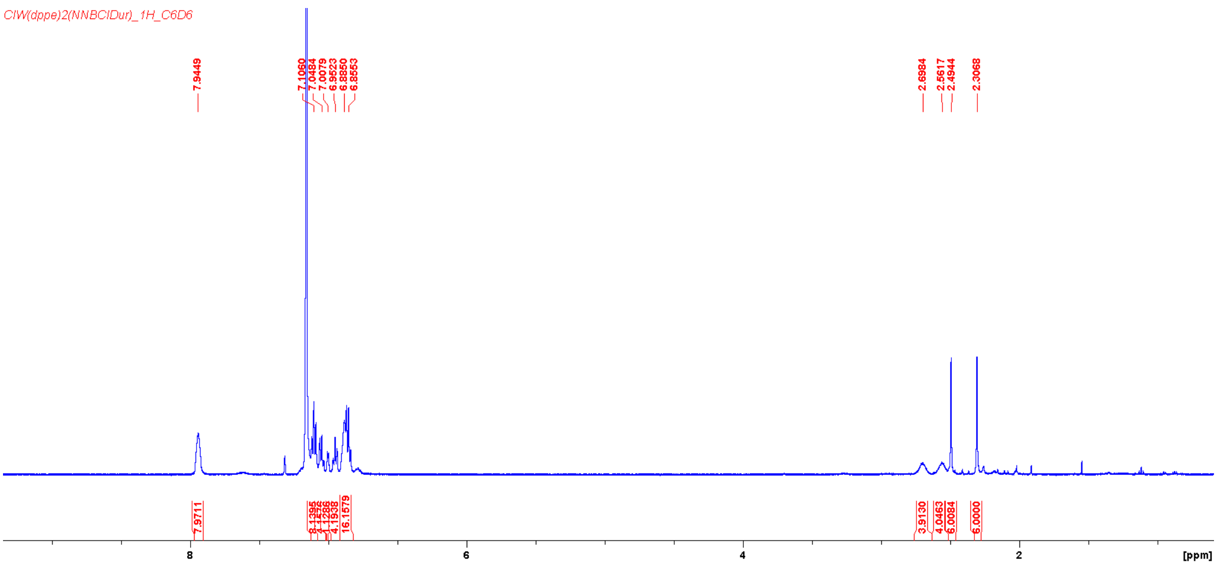


**Figure S43.** $^{1}$H NMR spectrum of **2f** in $C_6D_6$.

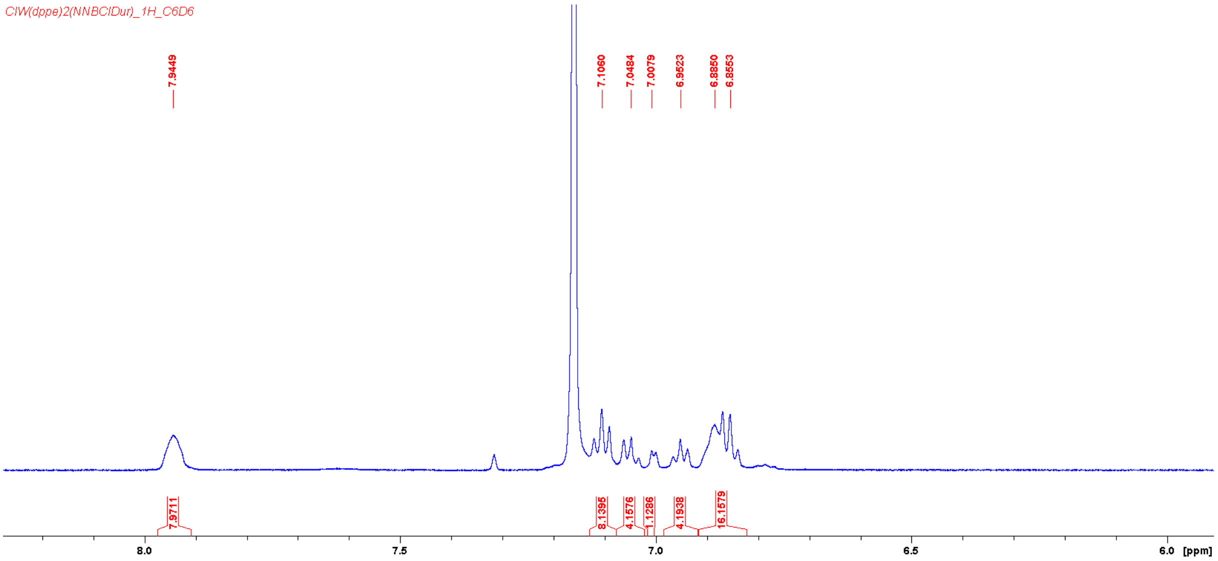


**Figure S44.** Expanded aromatic region of $^{1}H$ NMR spectrum of **2f** in $C_6D_6$.

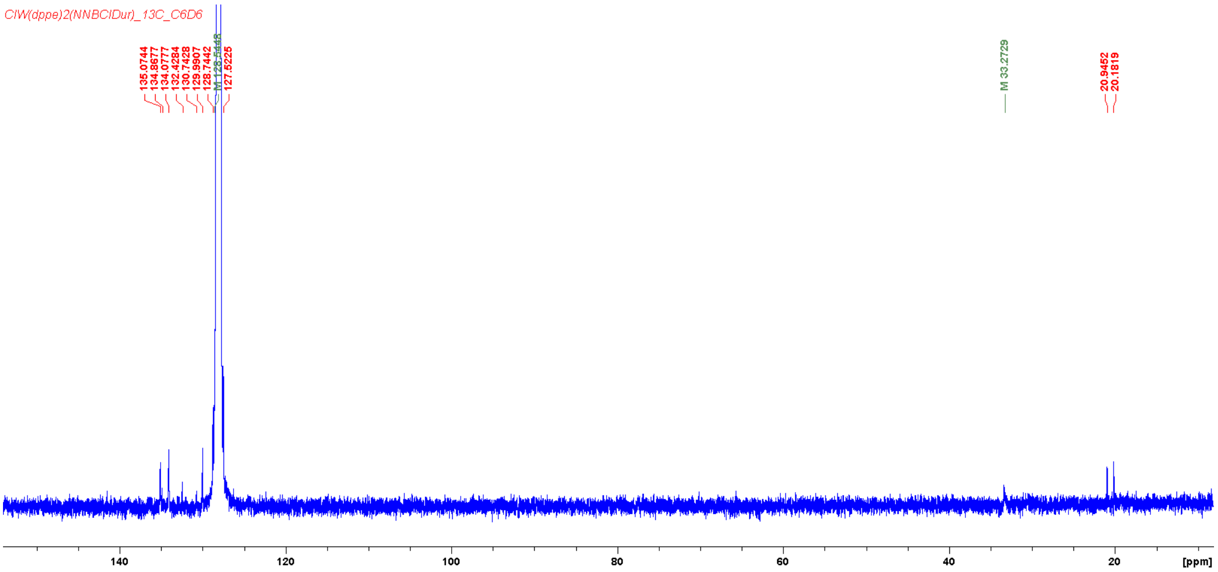


**Figure S45.** $^{13}C\{^{1}H\}$ NMR spectrum of **2f** in $C_6D_6$.

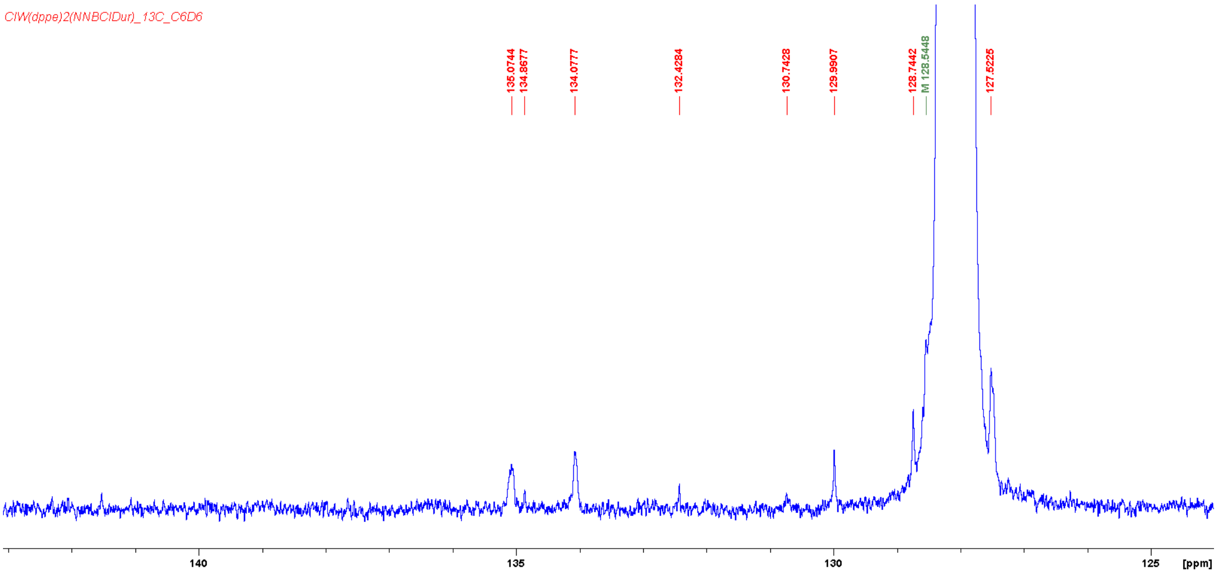


**Figure S46.** Expanded aromatic region of $^{13}C\{^{1}H\}$ NMR spectrum of **2f** in $C_6D_6$.

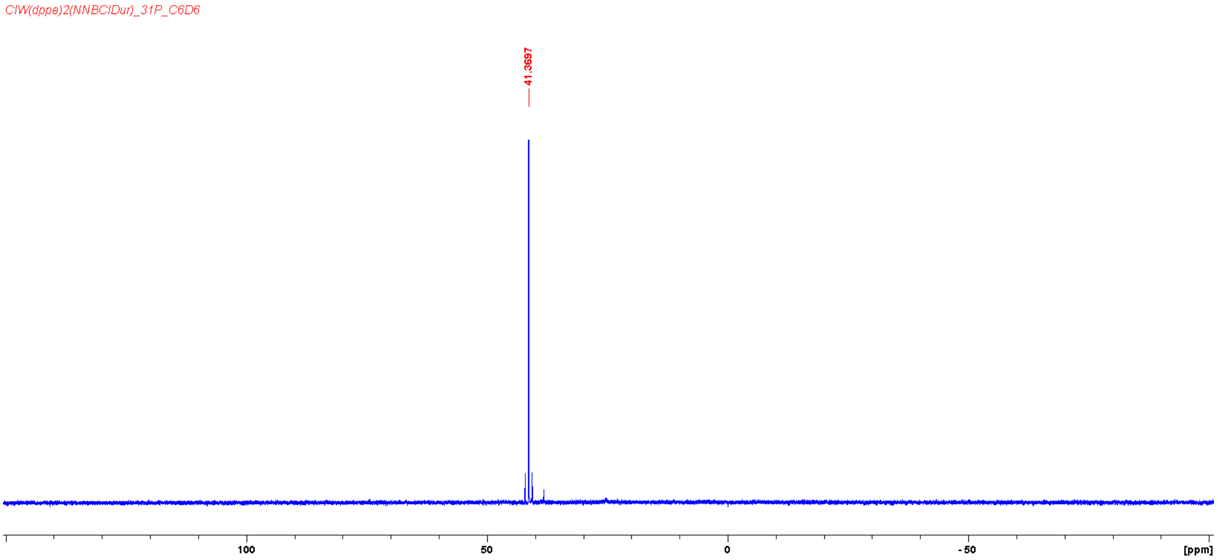


**Figure S47.** $^{31}P\{^{1}H\}$ NMR spectrum of **2f** in $C_6D_6$.

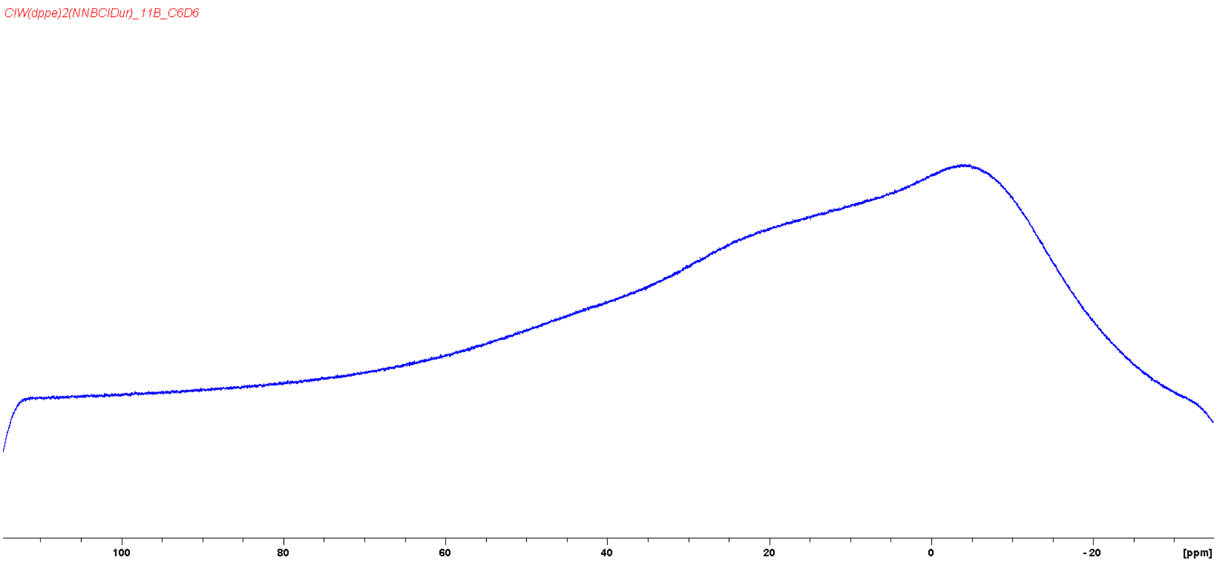


**Figure S48.** $^{11}B$ NMR spectrum of **2f** in $C_6D_6$.

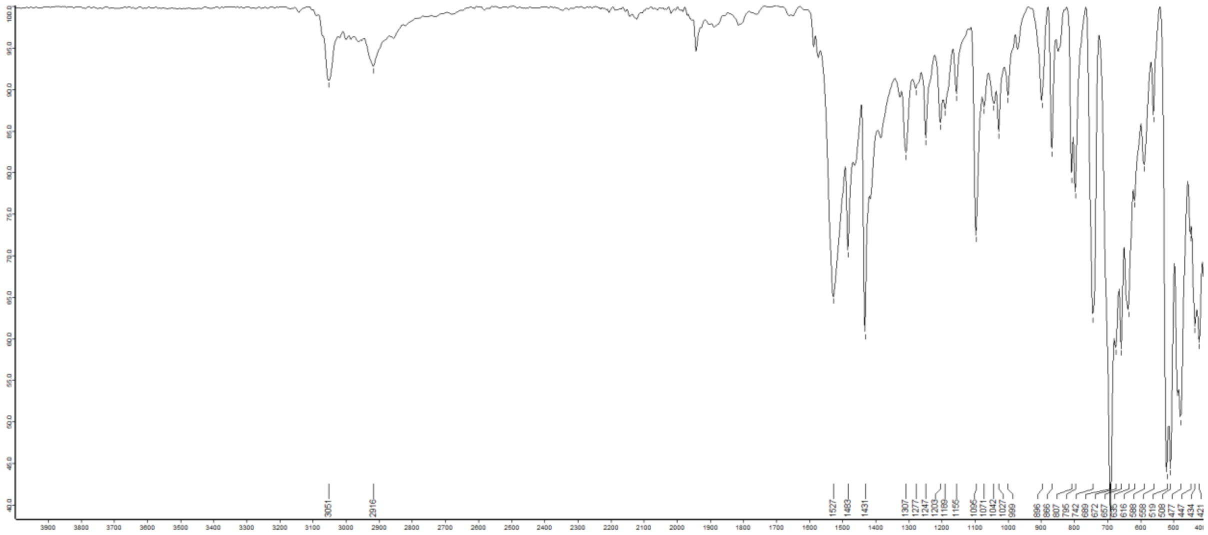


**Figure S49.** Solid-state IR spectrum of **2f**.

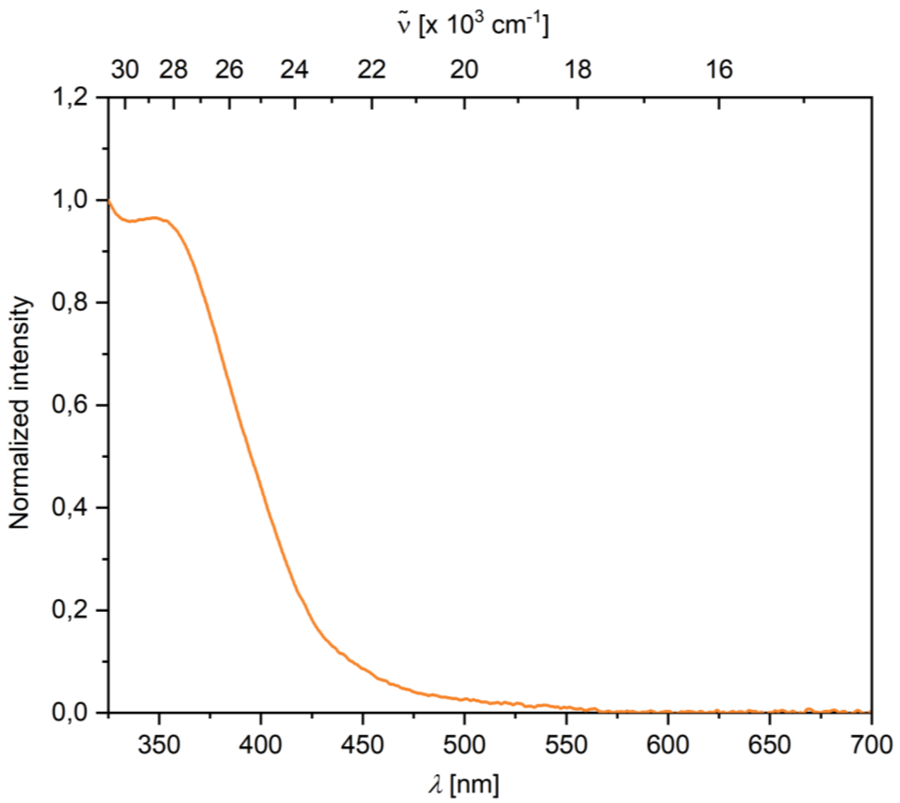


**Figure S50.** UV/Vis spectrum of **2f** in benzene.

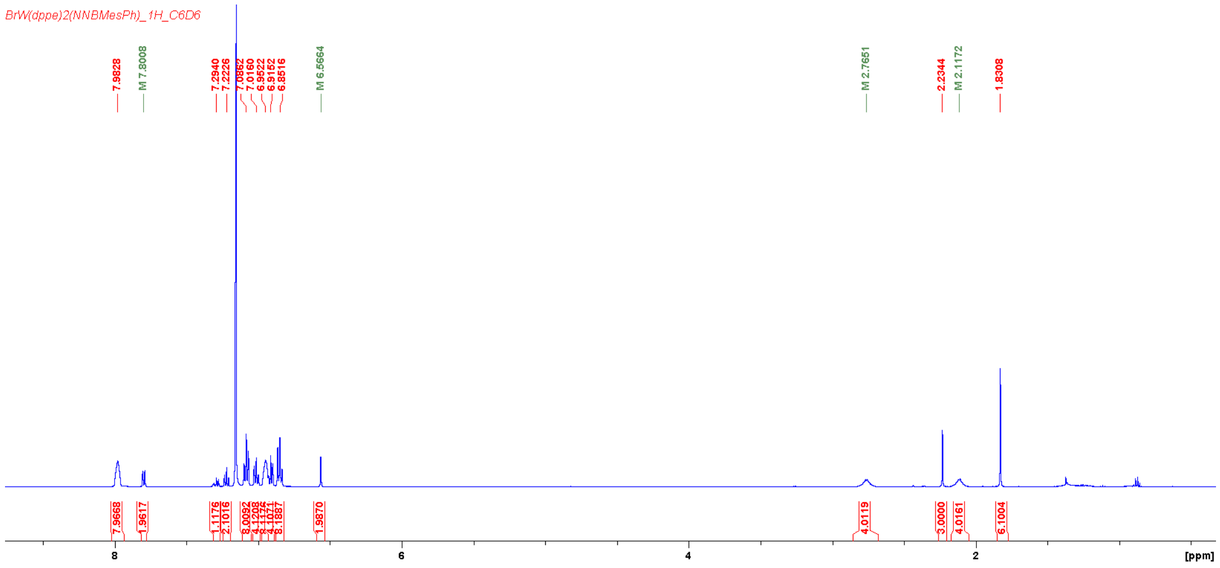


**Figure S51.** $^{1}H$ NMR spectrum of **2g** in $C_6D_6$.

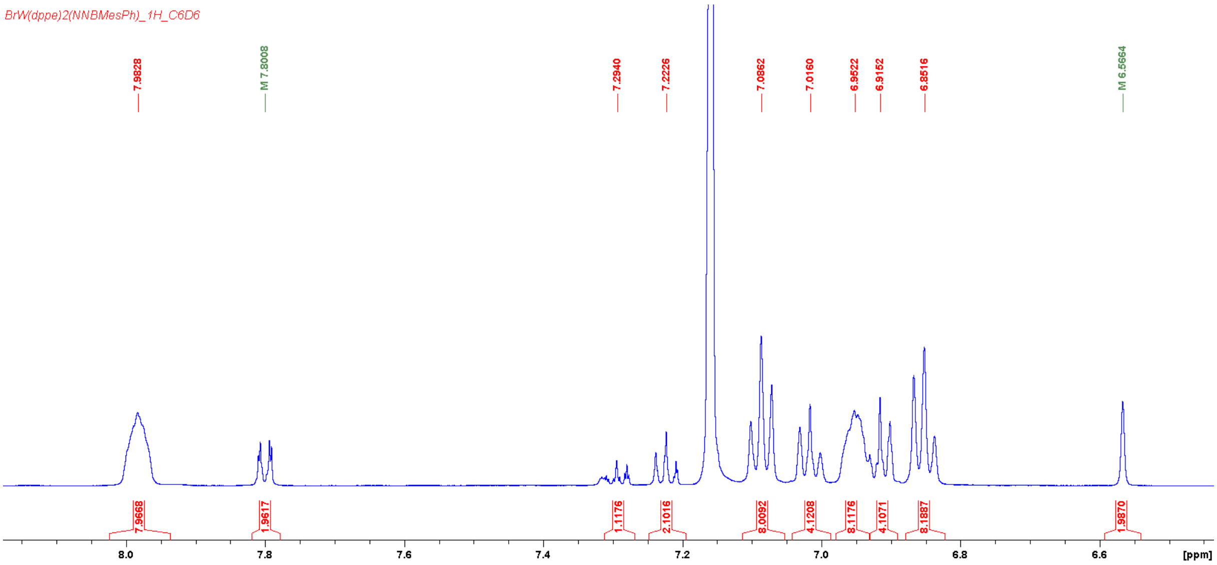


**Figure S52.** Expanded aromatic region of $^{1}$H NMR spectrum of **2g** in $C_6D_6$.

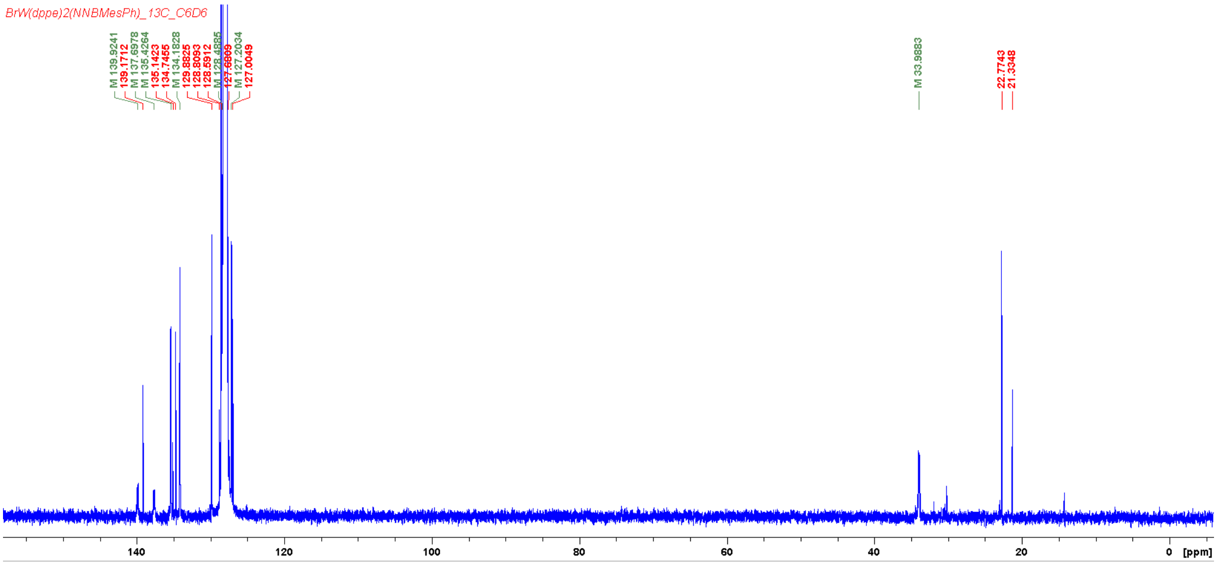


**Figure S53.** $^{13}C\{^{1}H\}$ NMR spectrum of **2g** in $C_6D_6$.

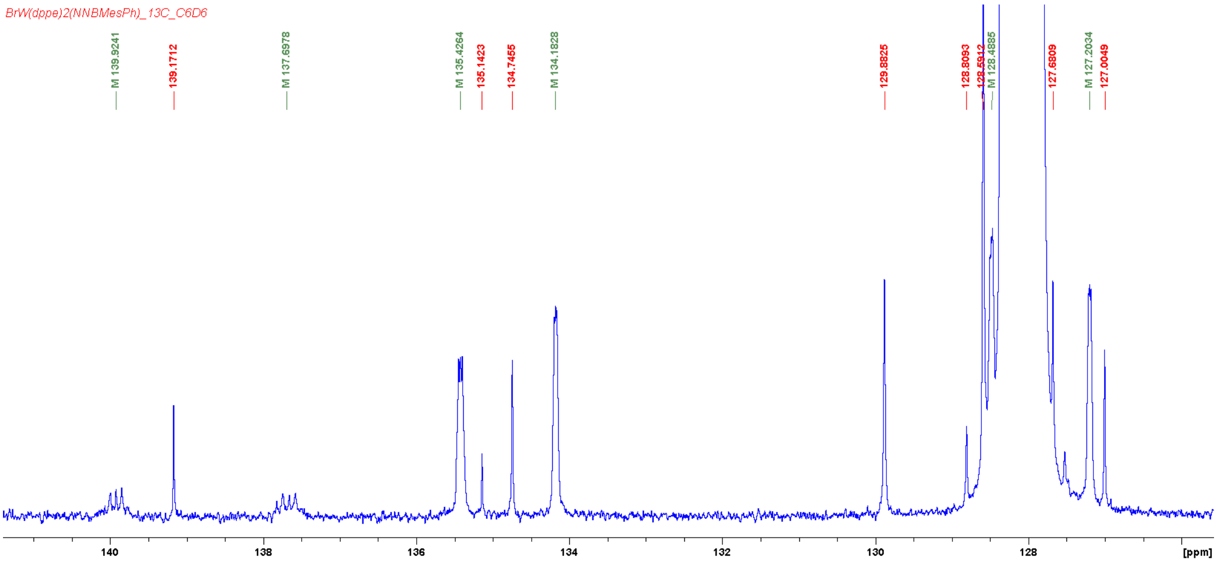


**Figure S54.** Expanded aromatic region of $^{13}C\{^1H\}$ NMR spectrum of **2g** in $C_6D_6$.

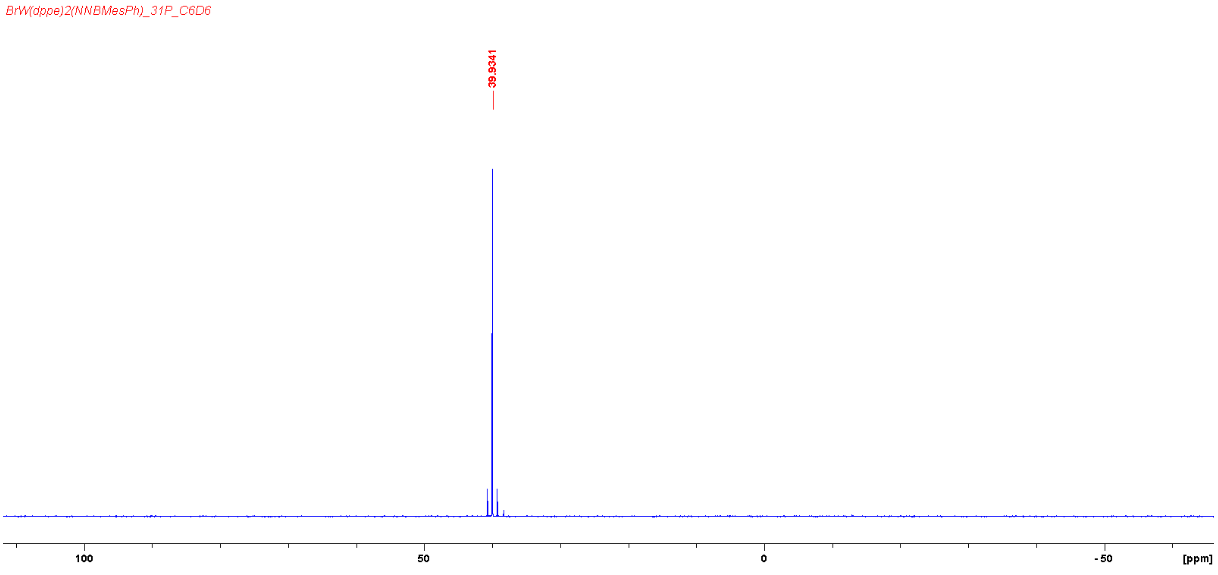


**Figure S55.** $^{31}P\{^1H\}$ NMR spectrum of **2g** in $C_6D_6$.

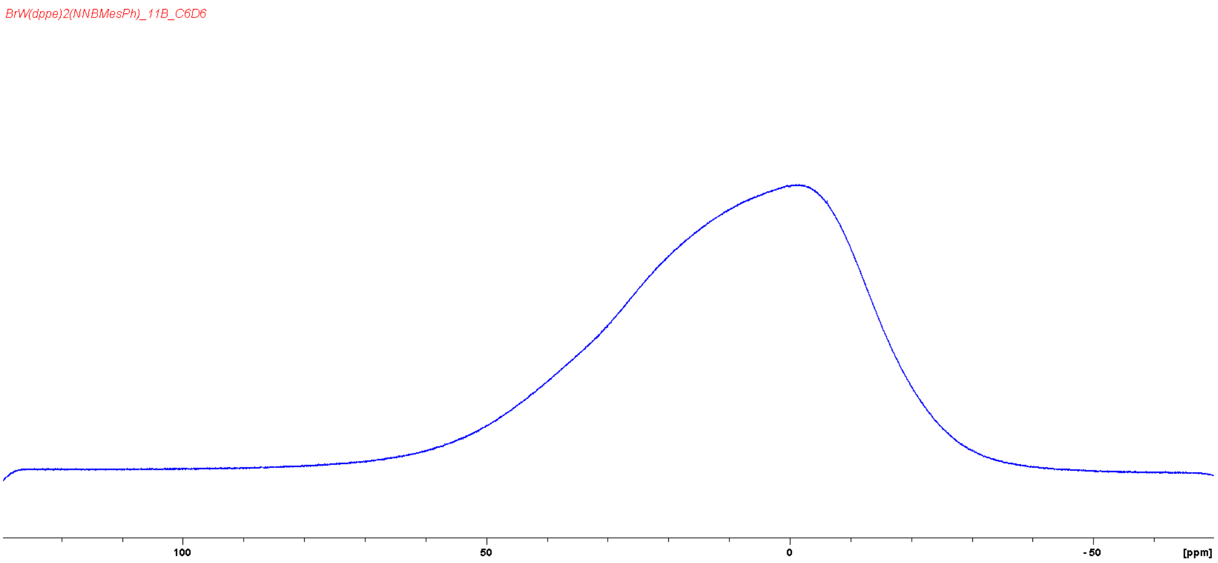


**Figure S56.** $^{11}$B NMR spectrum of **2g** in $C_6D_6$.

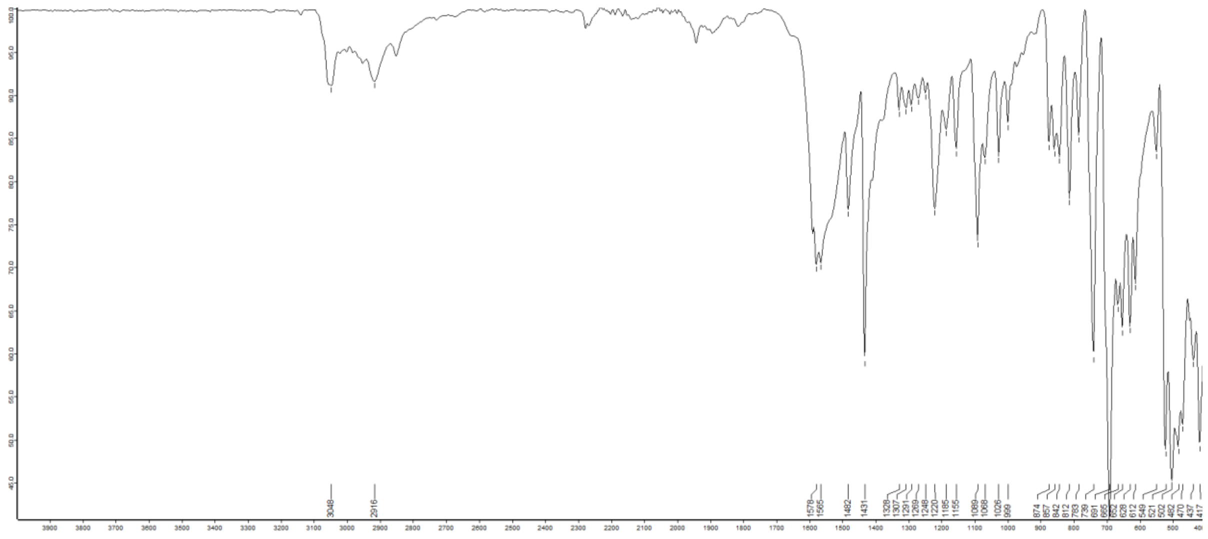

**Figure S57.** Solid-state IR spectrum of **2g**.

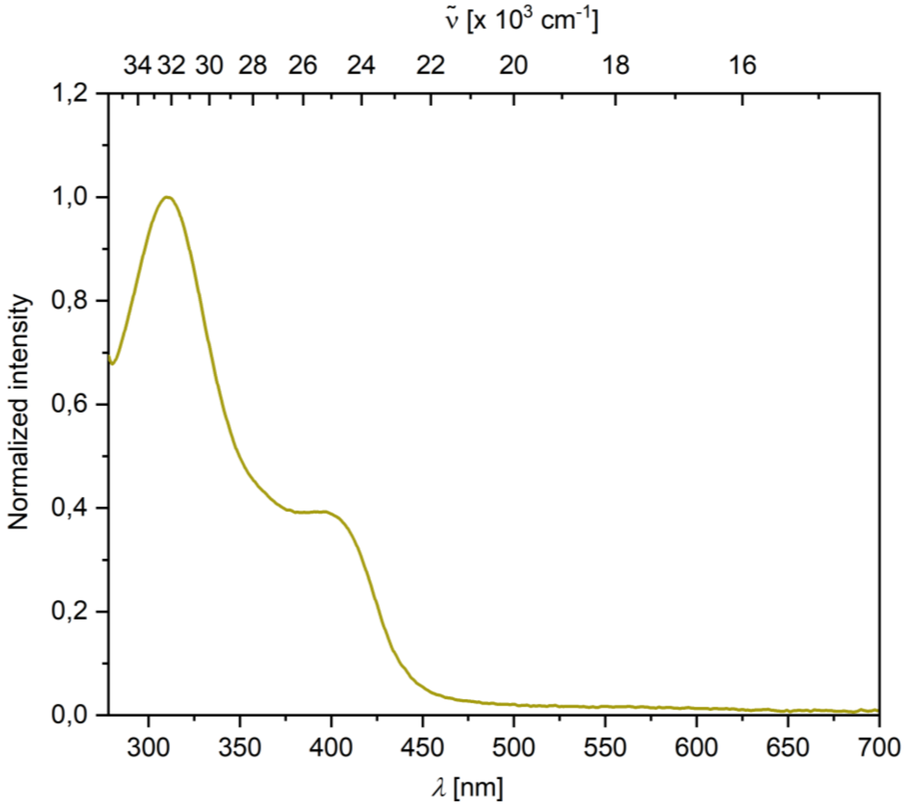


**Figure S58.** UV/Vis spectrum of **2g** in benzene.

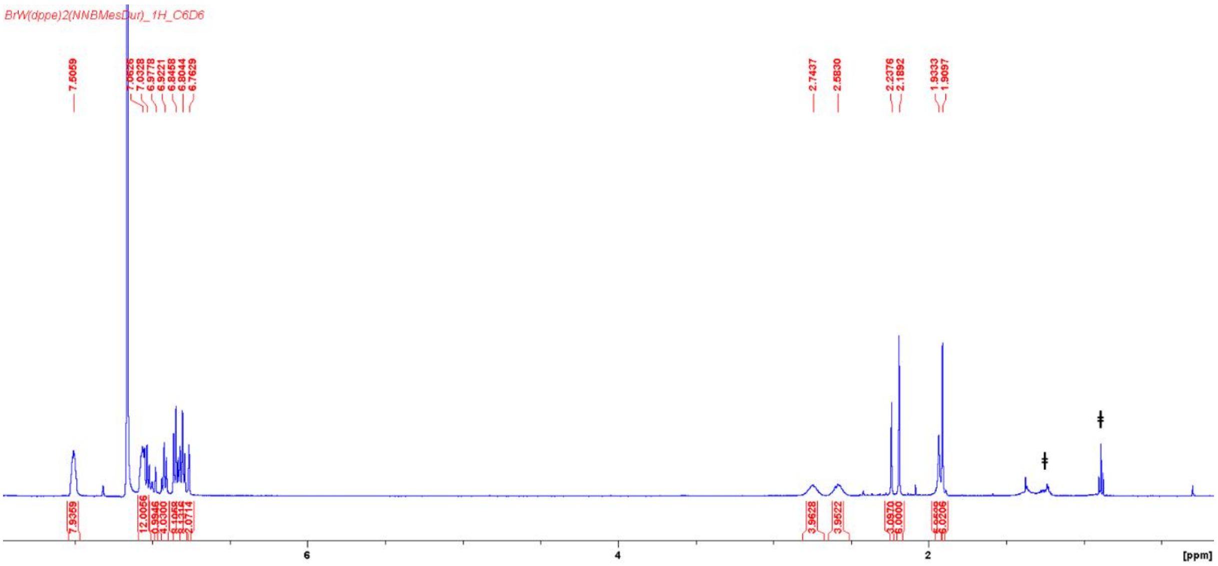


**Figure S59.** $^{1}H$ NMR spectrum of **2h** in $C_6D_6$. Resonances marked with ƚ correspond to residual hexane.

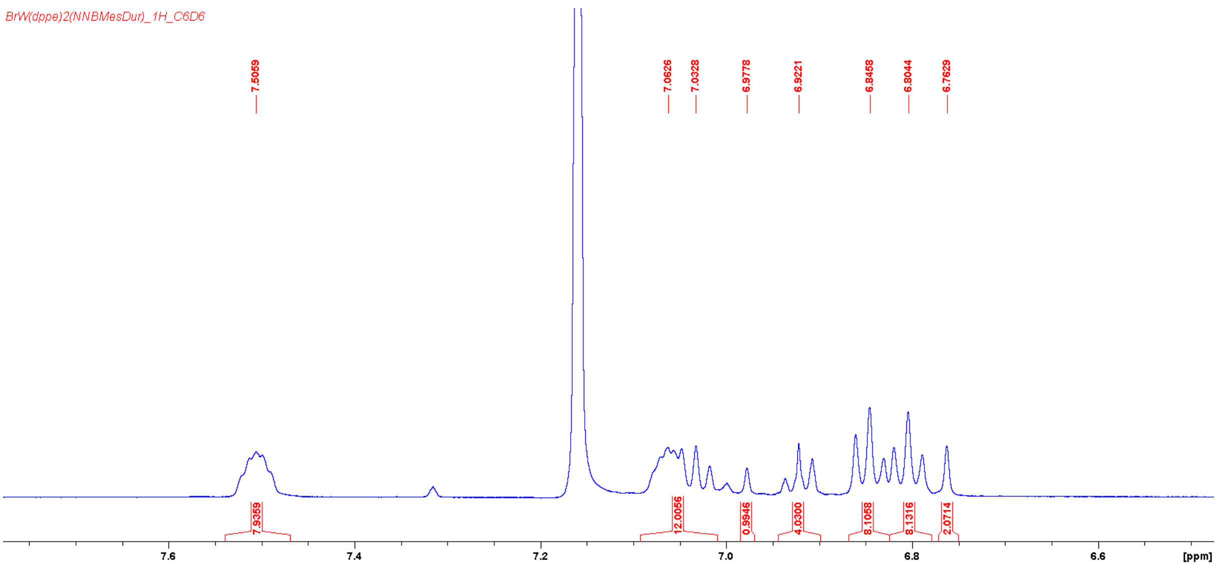


**Figure S60.** Expanded aromatic region of $^{1}$H NMR spectrum of **2h** in $C_6D_6$.

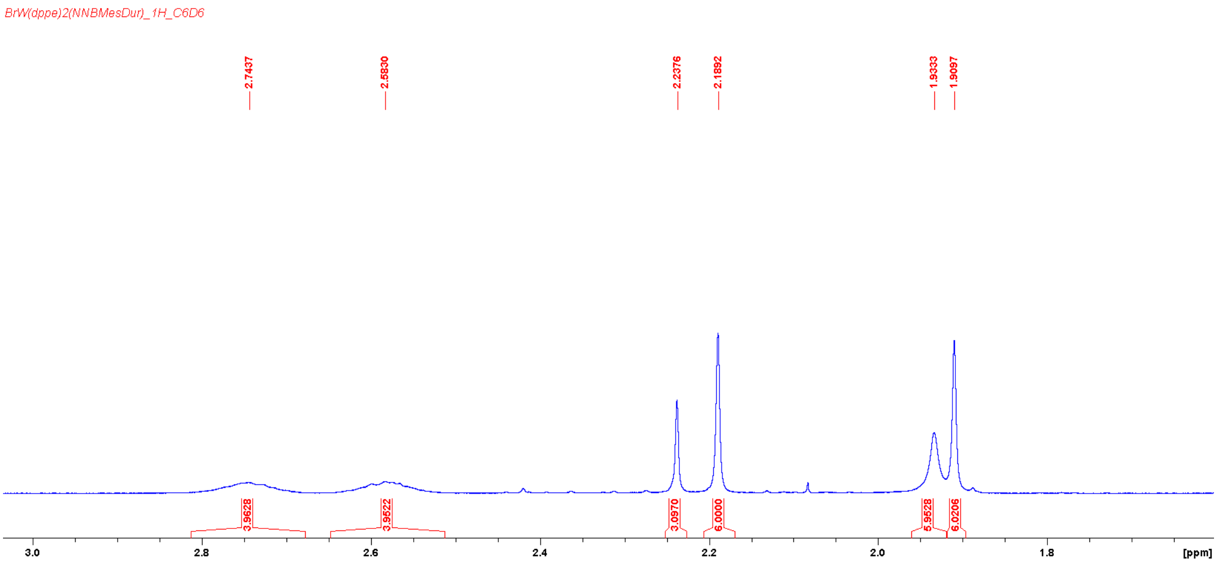


**Figure S61.** Expanded aliphatic region of $^{1}$H NMR spectrum of **2h** in $C_6D_6$.

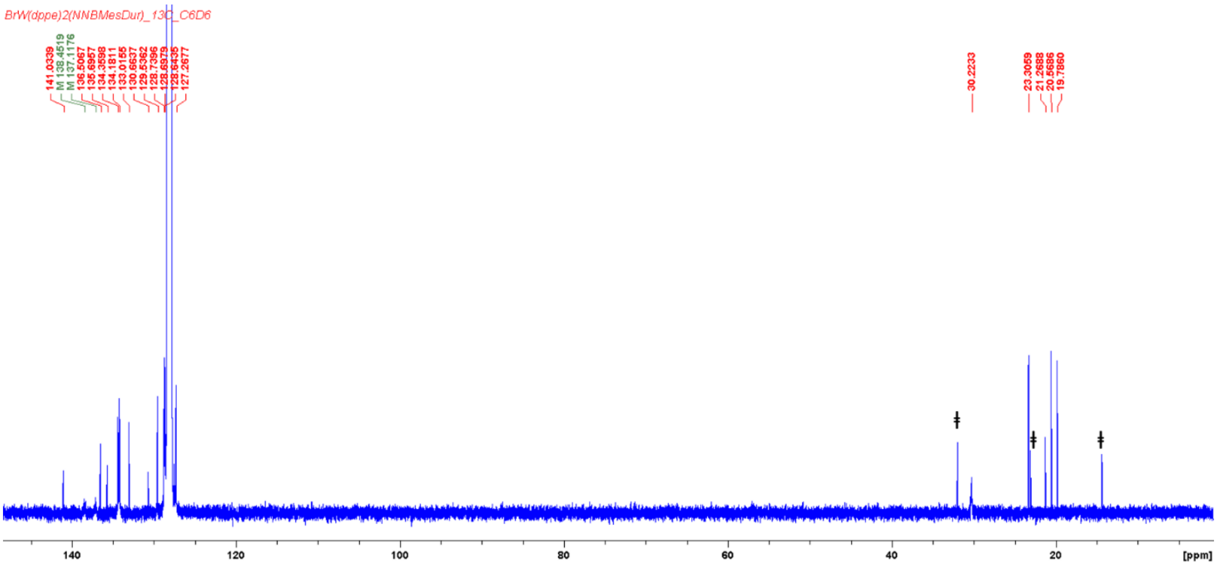


**Figure S62.** $^{13}C\{^{1}H\}$ NMR spectrum of **2h** in $C_6D_6$. Resonances marked with ǂ correspond to residual hexane.

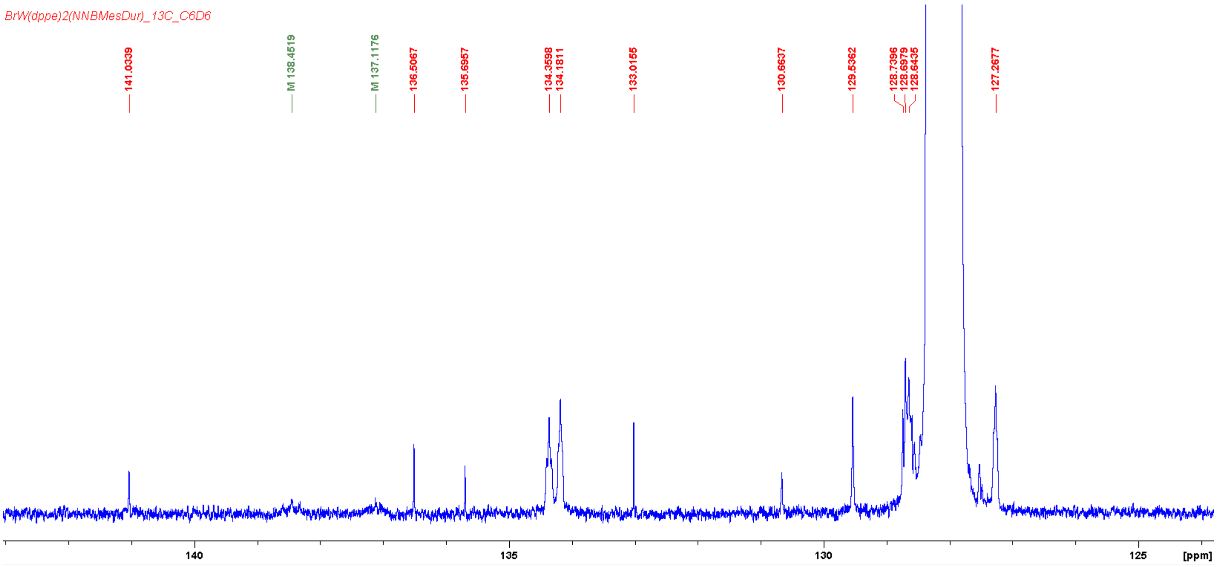


**Figure S63.** Expanded aromatic region of $^{13}C\{^{1}H\}$ NMR spectrum of **2h** in $C_6D_6$.

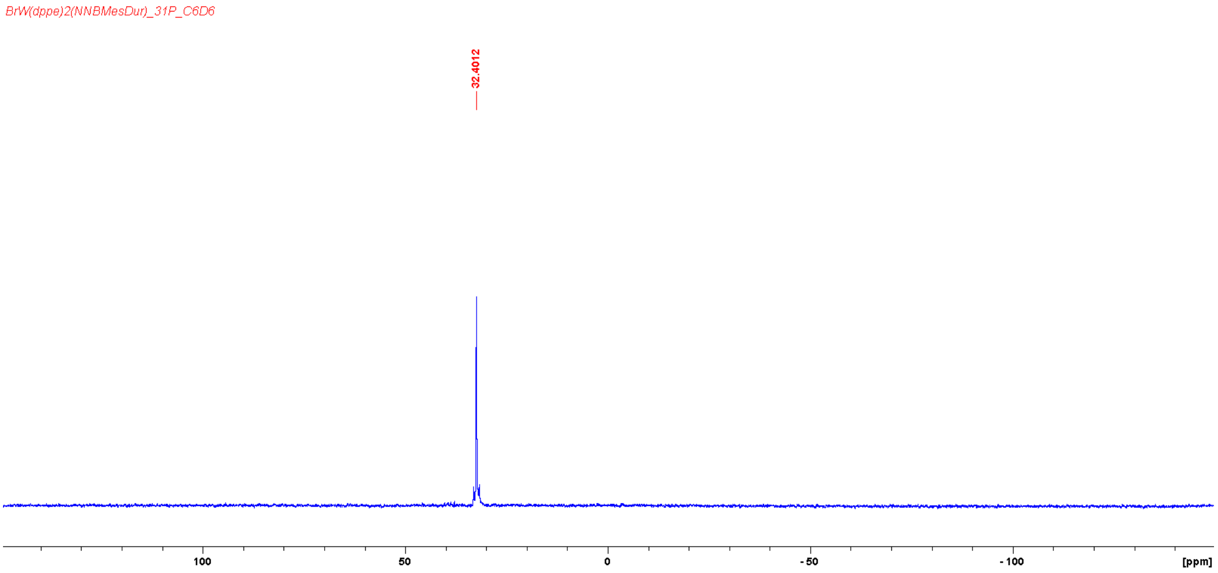


**Figure S64.** $^{31}P\{^{1}H\}$ NMR spectrum of **2h** in $C_6D_6$.

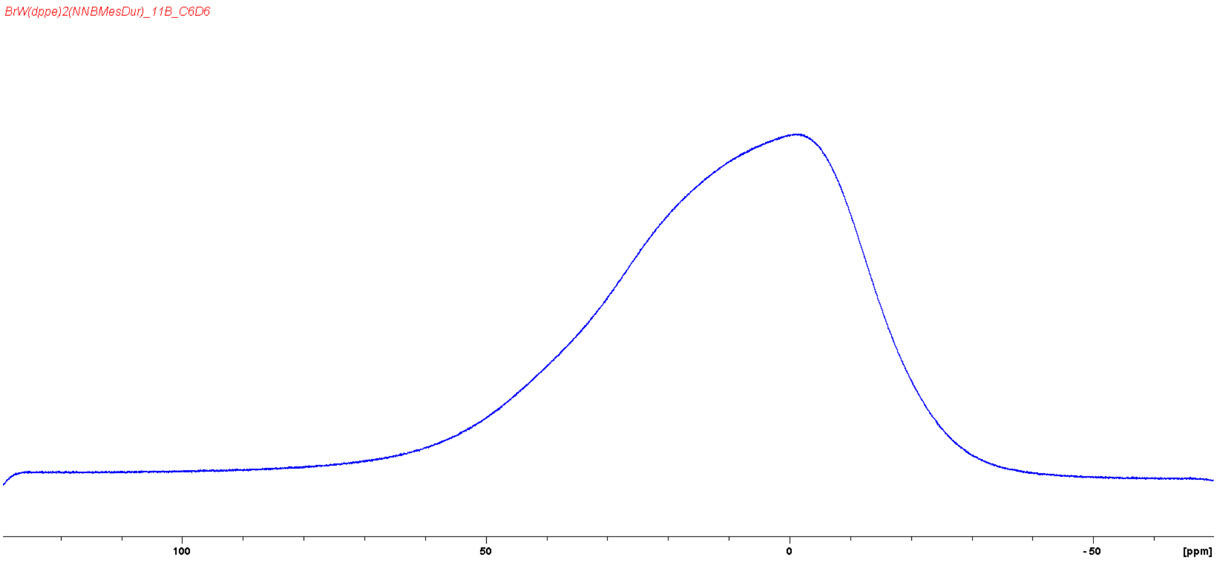


**Figure S65.** $^{11}B$ NMR spectrum of **2h** in $C_6D_6$.

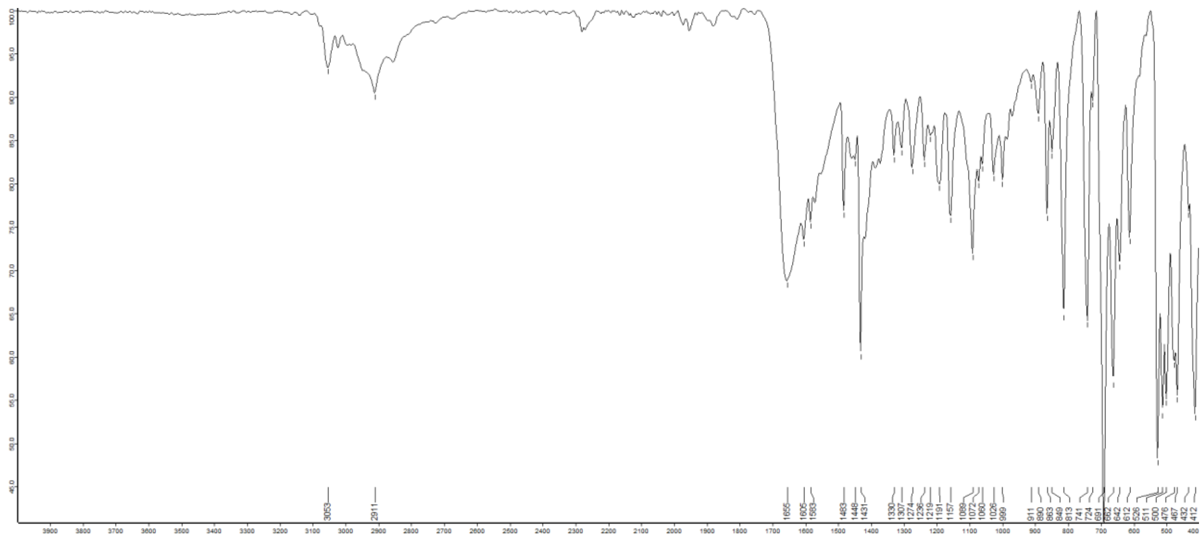

**Figure S66.** Solid-state IR spectrum of **2h**.

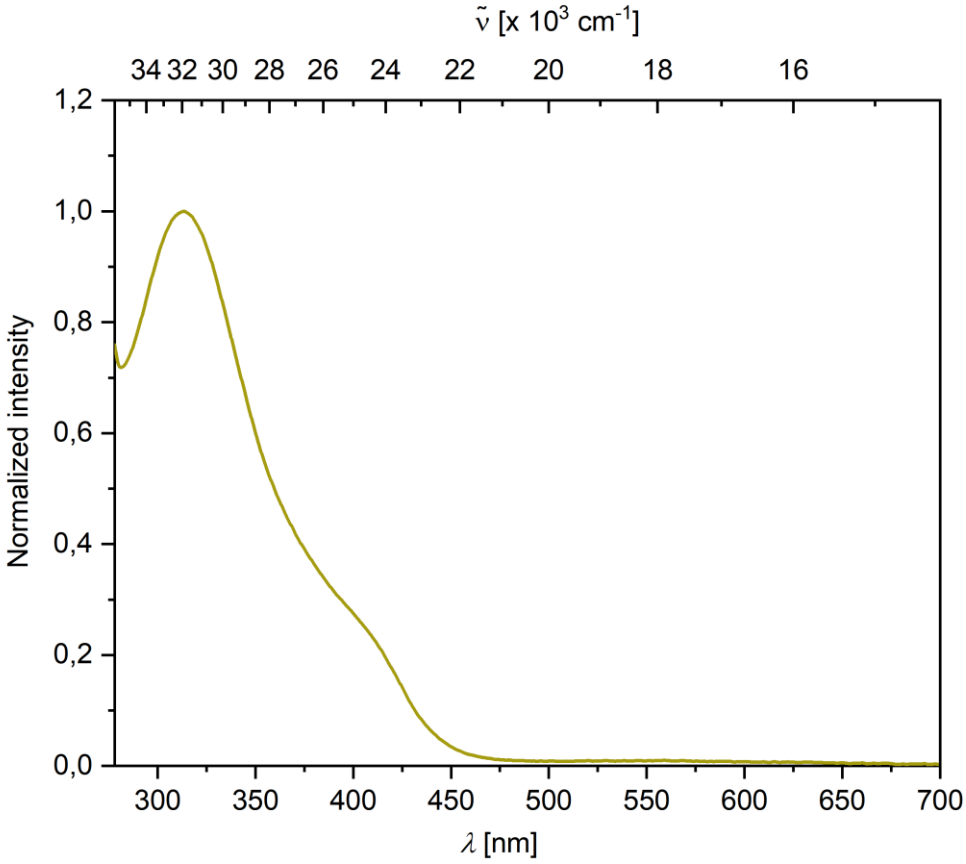


**Figure S67.** UV/Vis spectrum of **2h** in benzene.

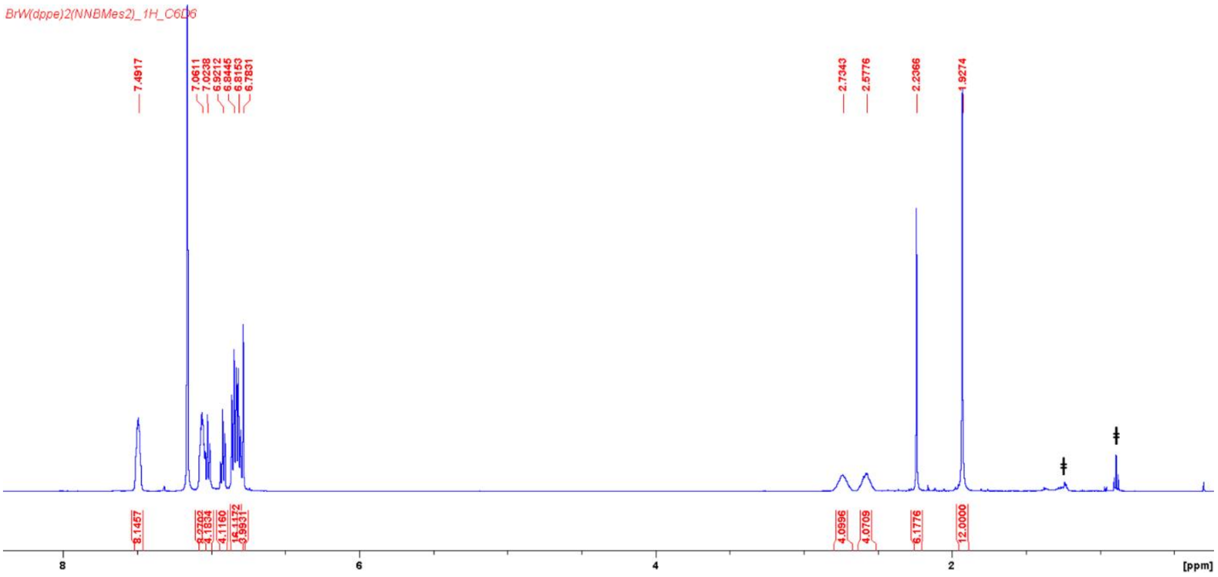


**Figure S68.** $^{1}H$ NMR spectrum of **2i** in $C_6D_6$. Resonances marked with ƚ correspond to residual hexane.

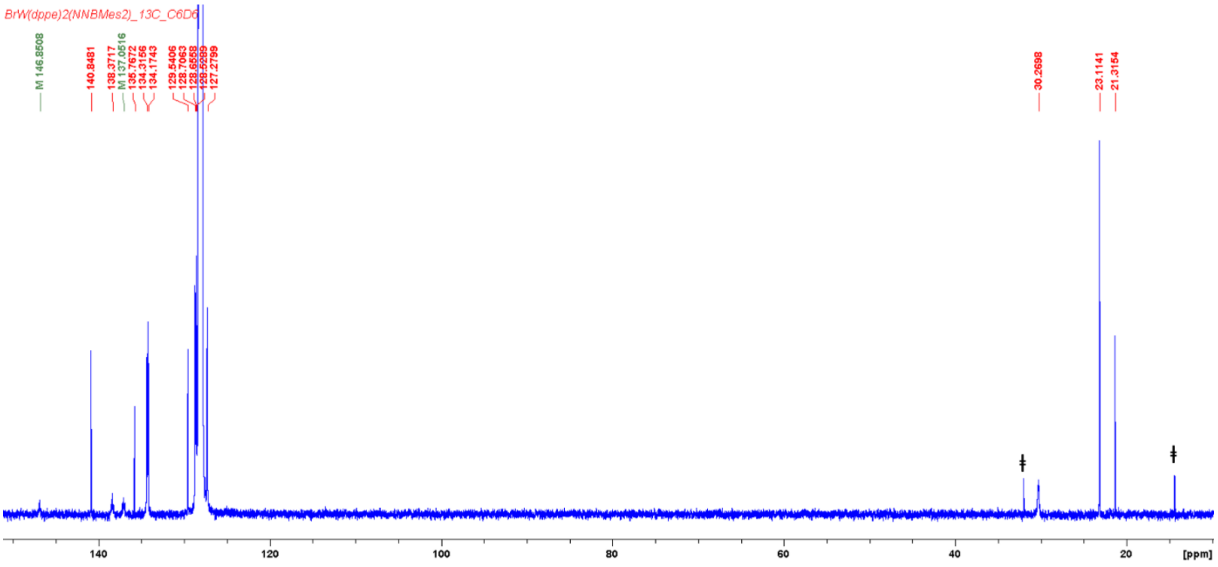


**Figure S69.** $^{13}C\{^{1}H\}$ NMR spectrum of **2i** in $C_6D_6$. Resonances marked with ƚ correspond to residual hexane.

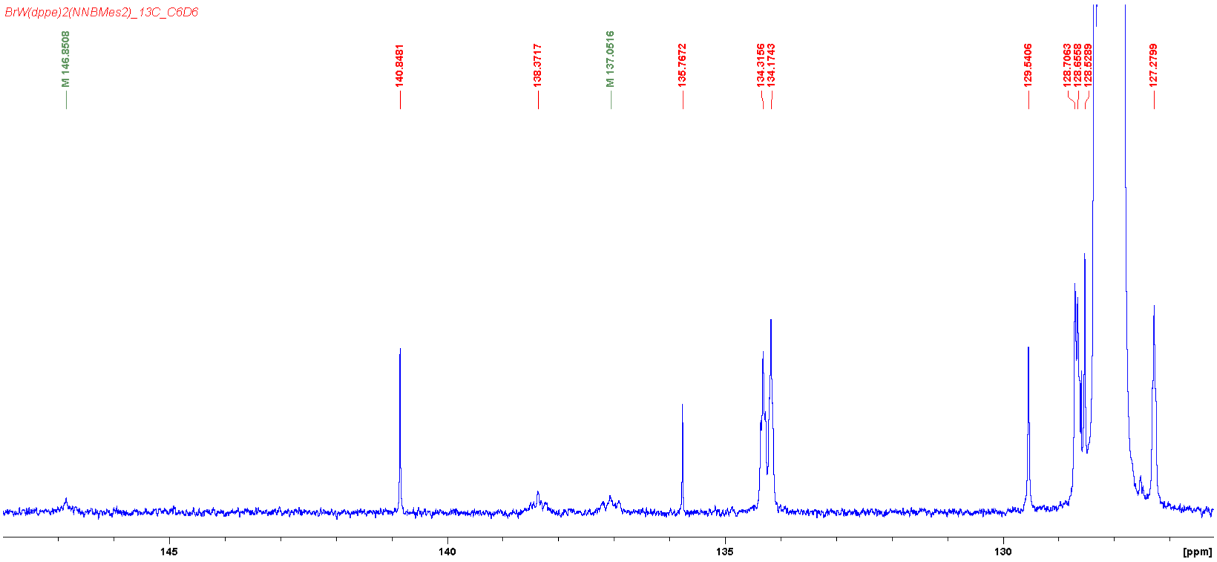


**Figure S70.** Expanded aromatic region of $^{13}C\{^{1}H\}$ NMR spectrum of **2i** in $C_6D_6$.

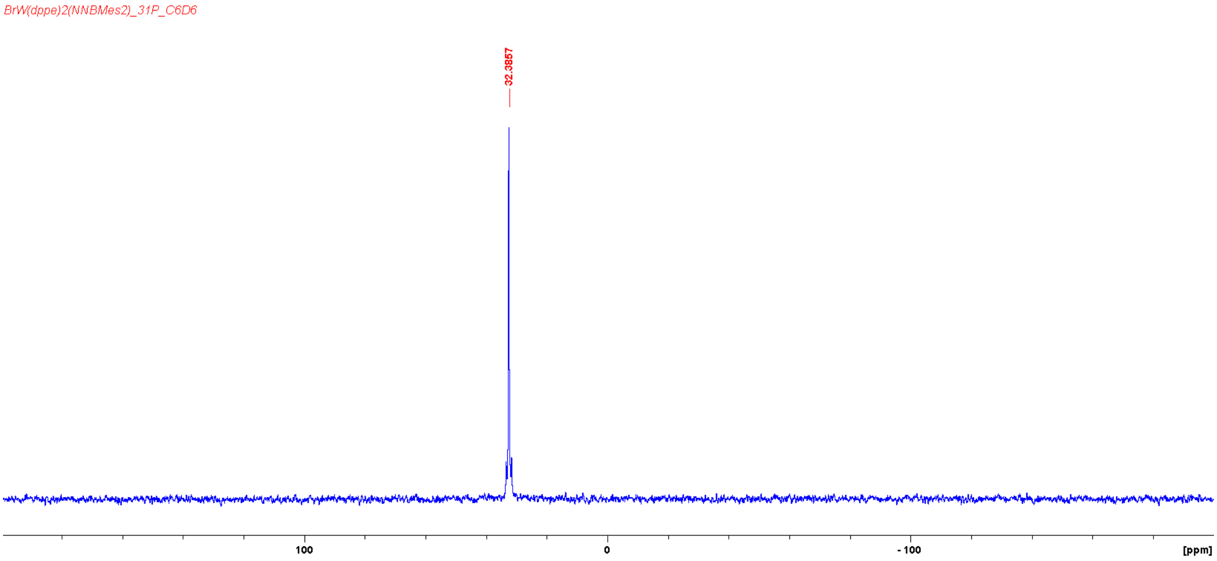


**Figure S71.** $^{31}P\{^{1}H\}$ NMR spectrum of **2i** in $C_6D_6$.

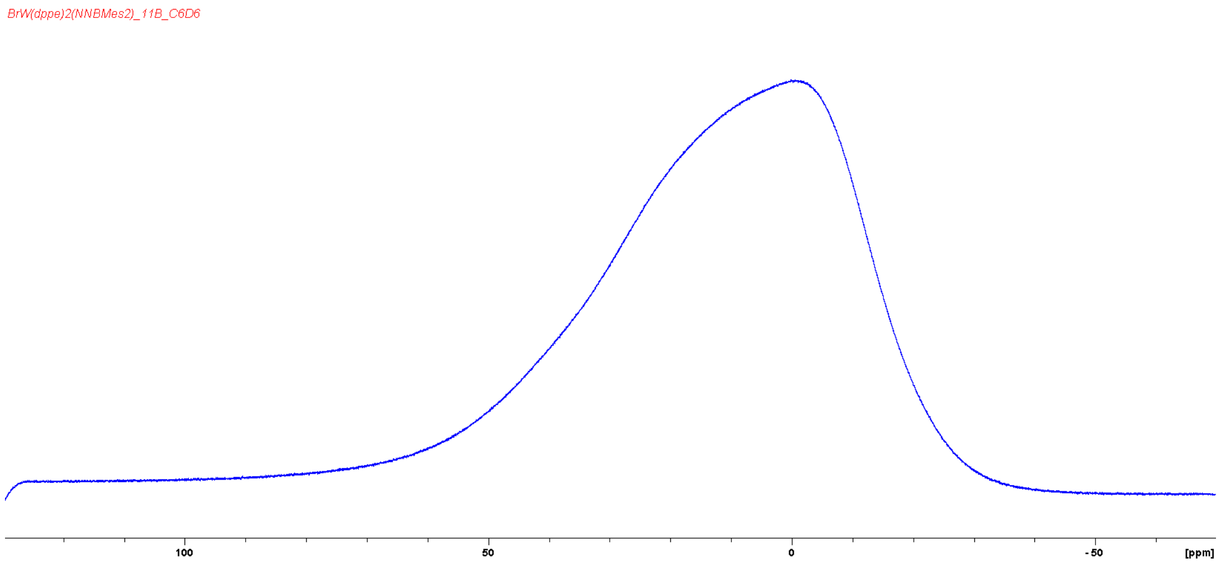


**Figure S72.** $^{11}B$ NMR spectrum of **2i** in $C_6D_6$.

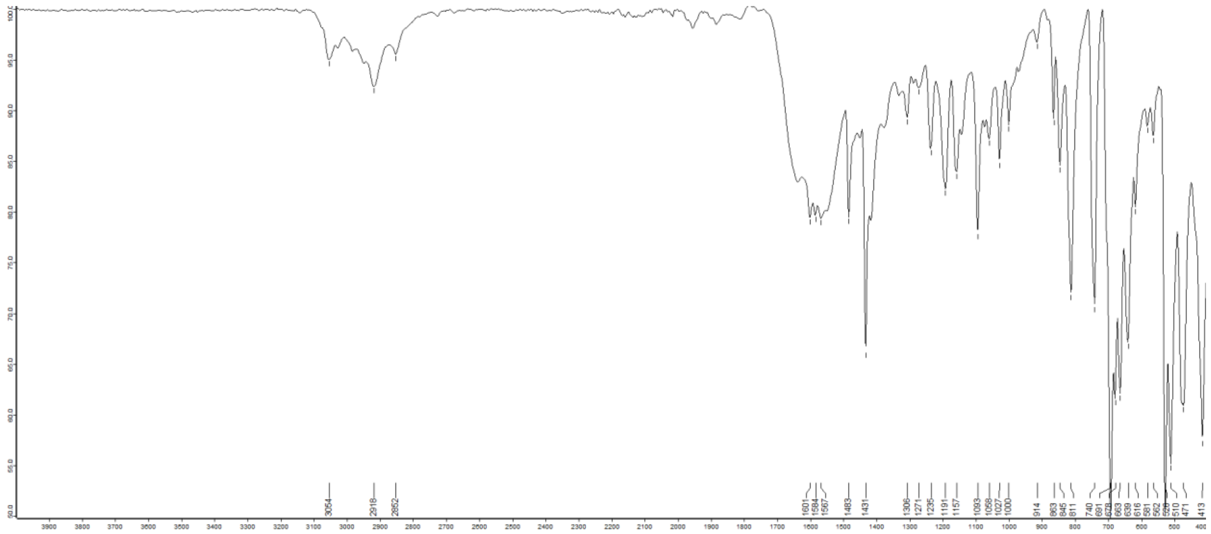

**Figure S73.** Solid-state IR spectrum of **2i**.

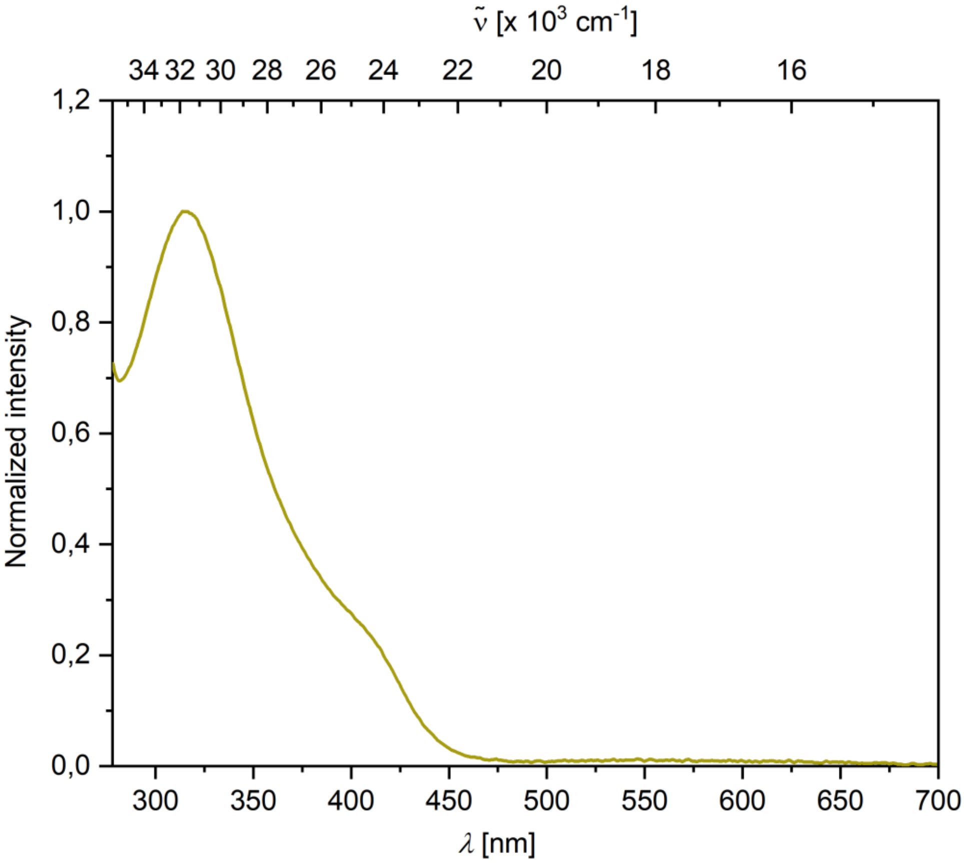


**Figure S74.** UV/Vis spectrum of **2i** in benzene.

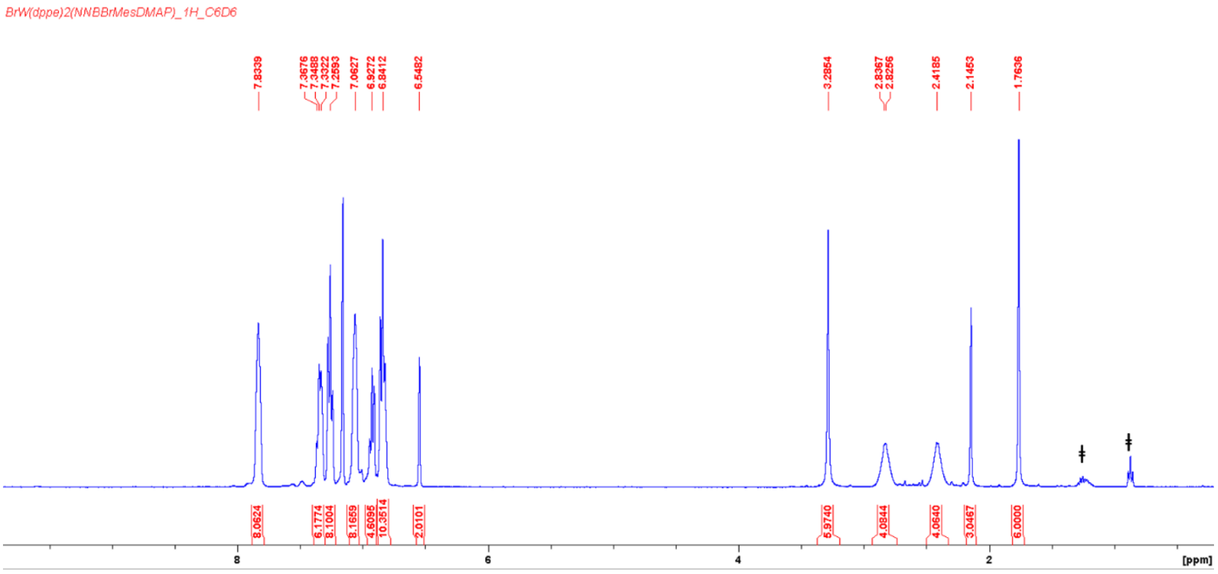


**Figure S75.** $^{1}$H NMR spectrum of **2j** in $C_6D_6$. Resonances marked with ǂ correspond to residual pentane.

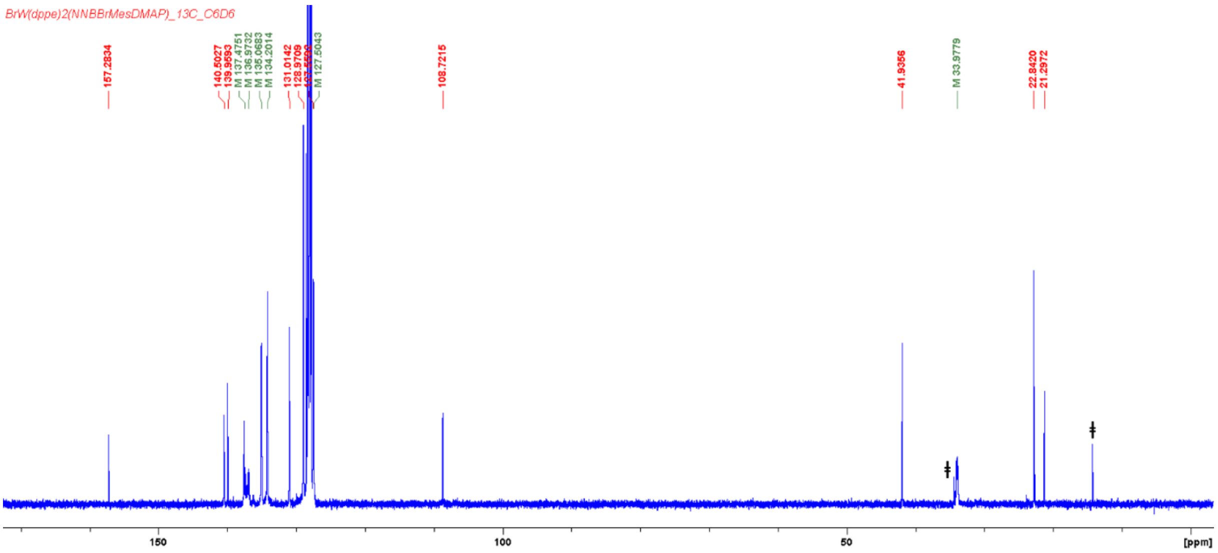


**Figure S76.** $^{13}C\{^{1}H\}$ NMR spectrum of **2j** in $C_6D_6$. Resonances marked with ‡ correspond to residual pentane.

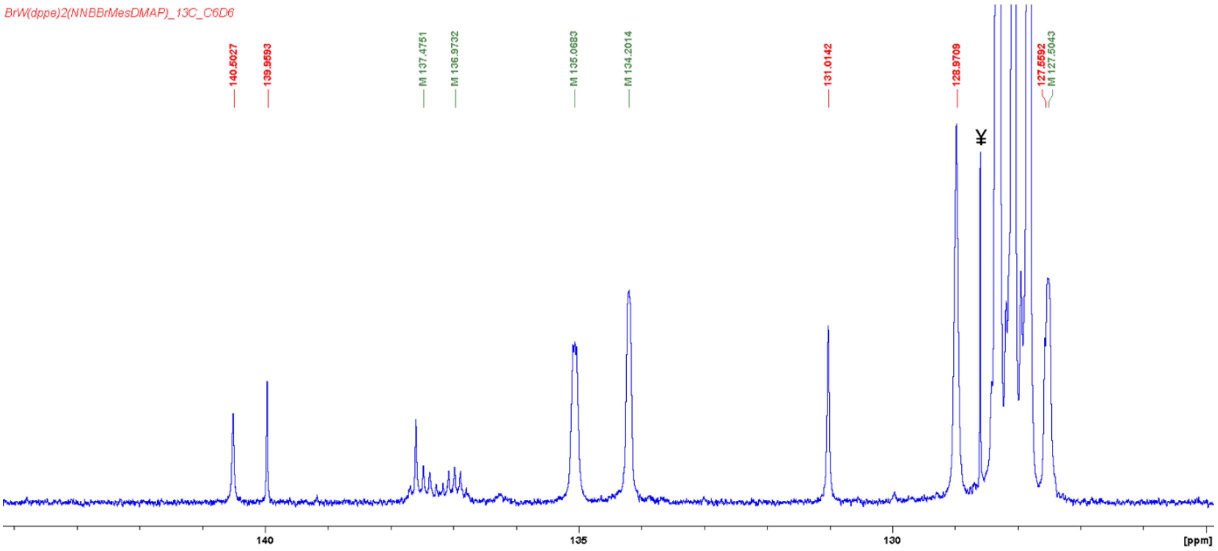


**Figure S77.** Expanded aromatic region of $^{13}C\{^{1}H\}$ NMR spectrum of **2j** in $C_6D_6$. Resonances marked with ¥ correspond to residual benzene.

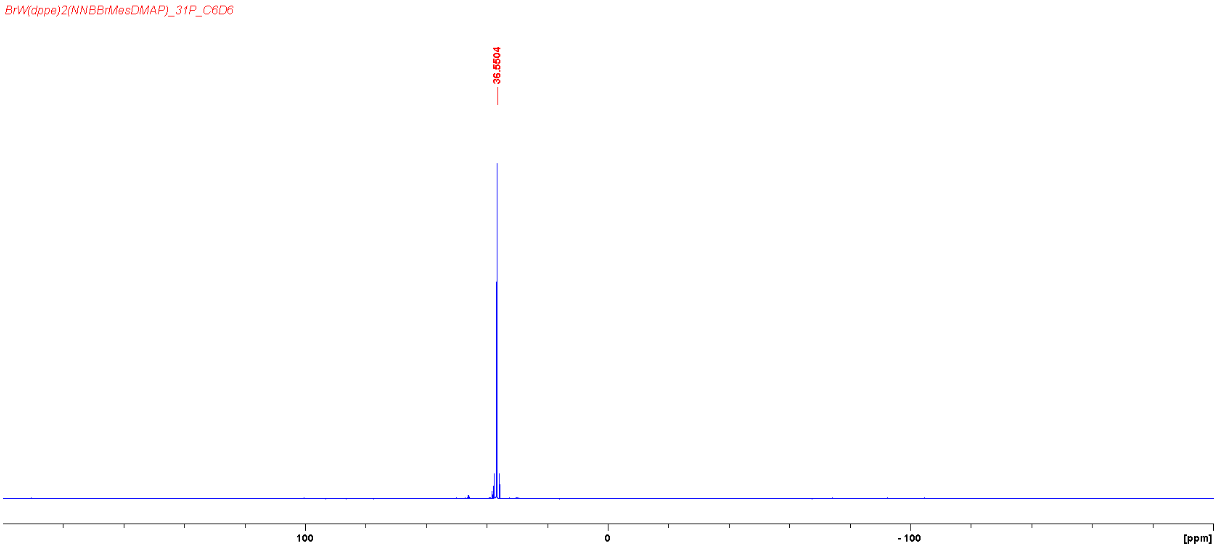


**Figure S78.** $^{31}P\{^{1}H\}$ NMR spectrum of **2j** in $C_6D_6$.

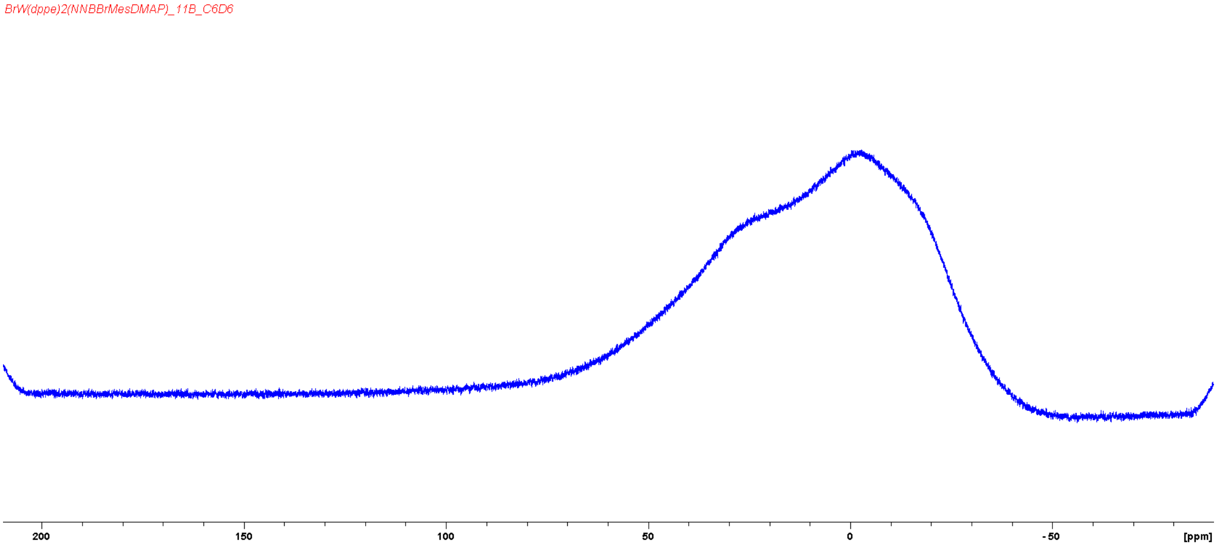


**Figure S79.** $^{11}B$ NMR spectrum of **2j** in $C_6D_6$.

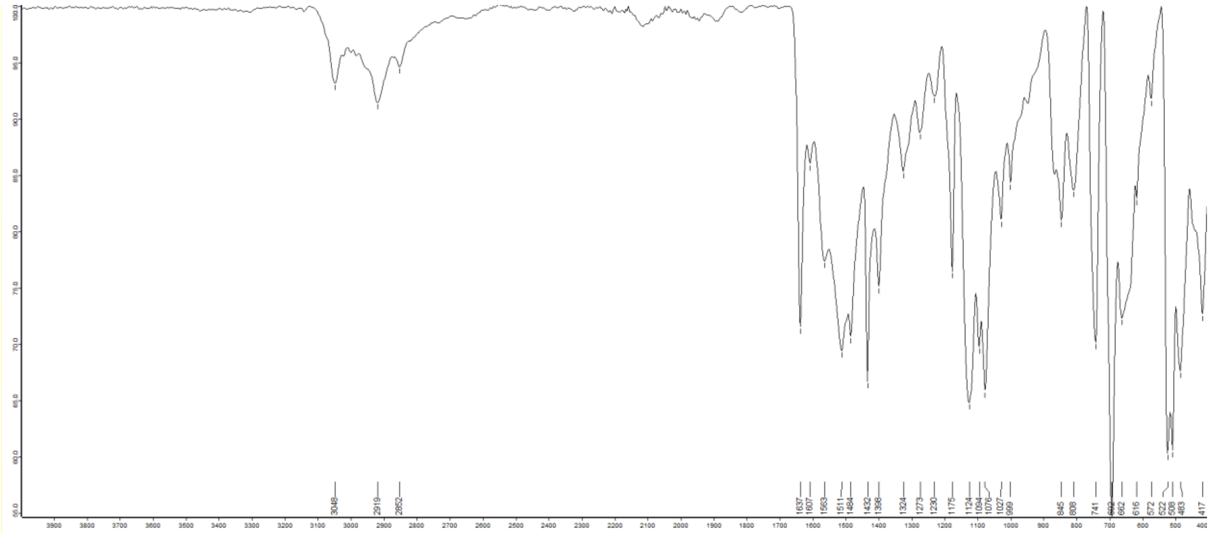


**Figure S80.** Solid-state IR spectrum of **2j**.

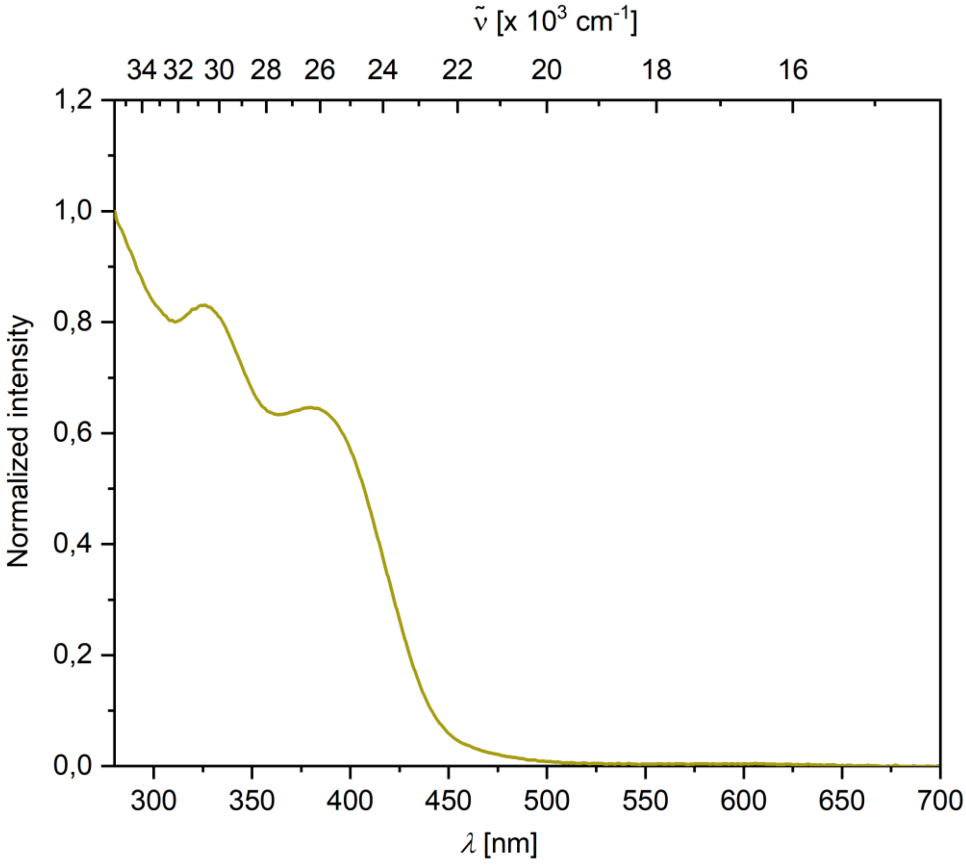


**Figure S81.** UV/Vis spectrum of **2j** in benzene.

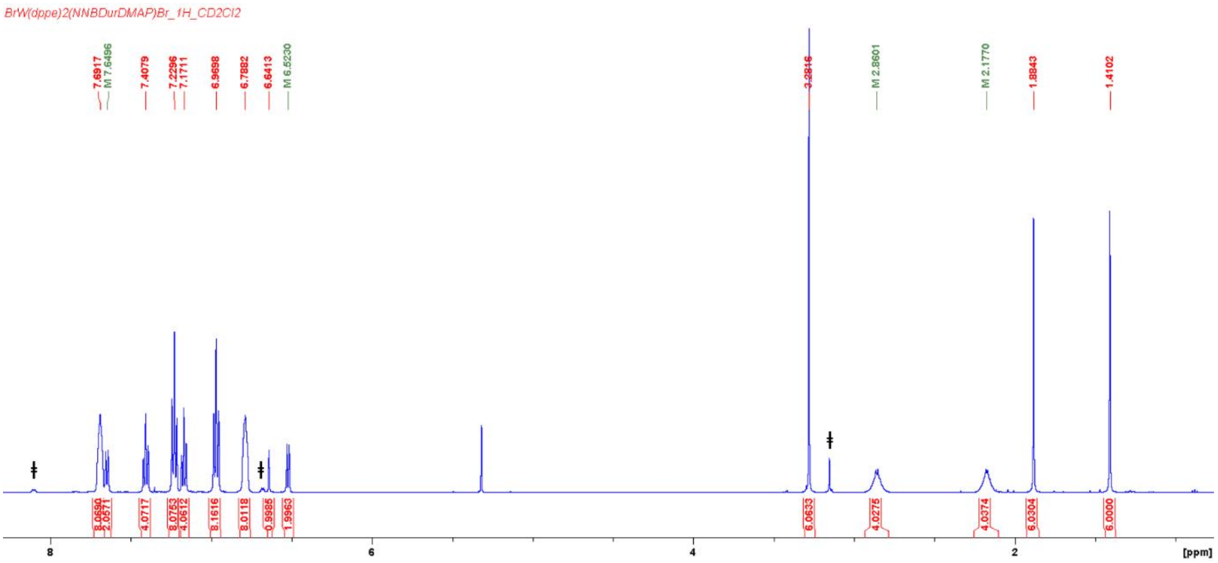


**Figure S82.** $^{1}H$ NMR spectrum of **2k** in $CD_2Cl_2$. Resonances marked with ɫ correspond to residual DMAP.

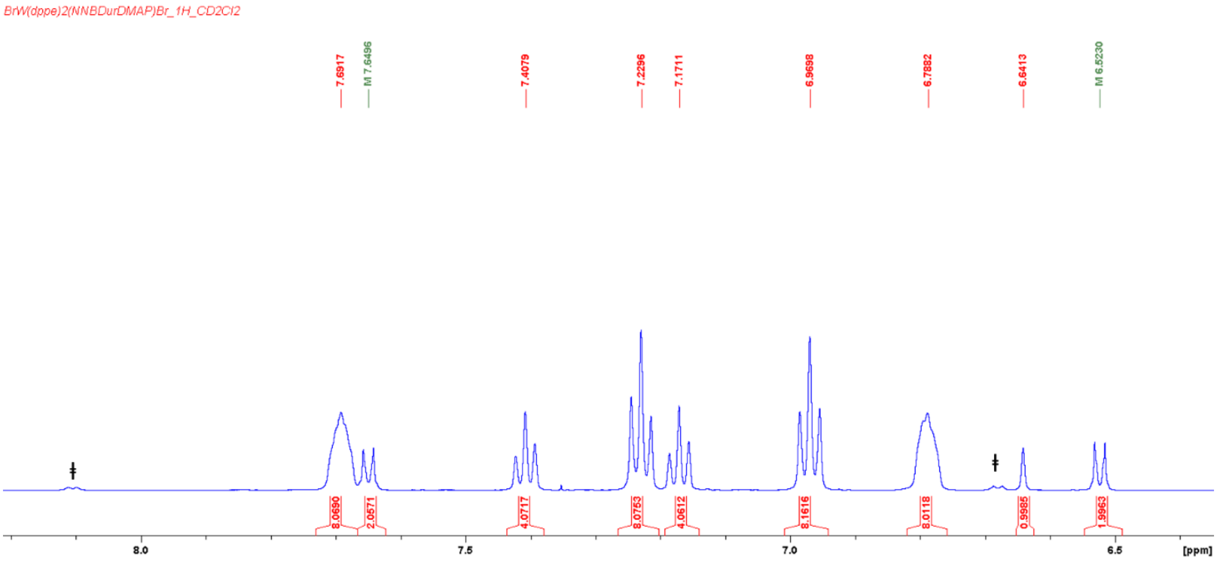


**Figure S83.** Expanded aromatic region of $^{1}H$ NMR spectrum of **2k** in $CD_2Cl_2$. Resonances marked with ɫ correspond to residual DMAP.

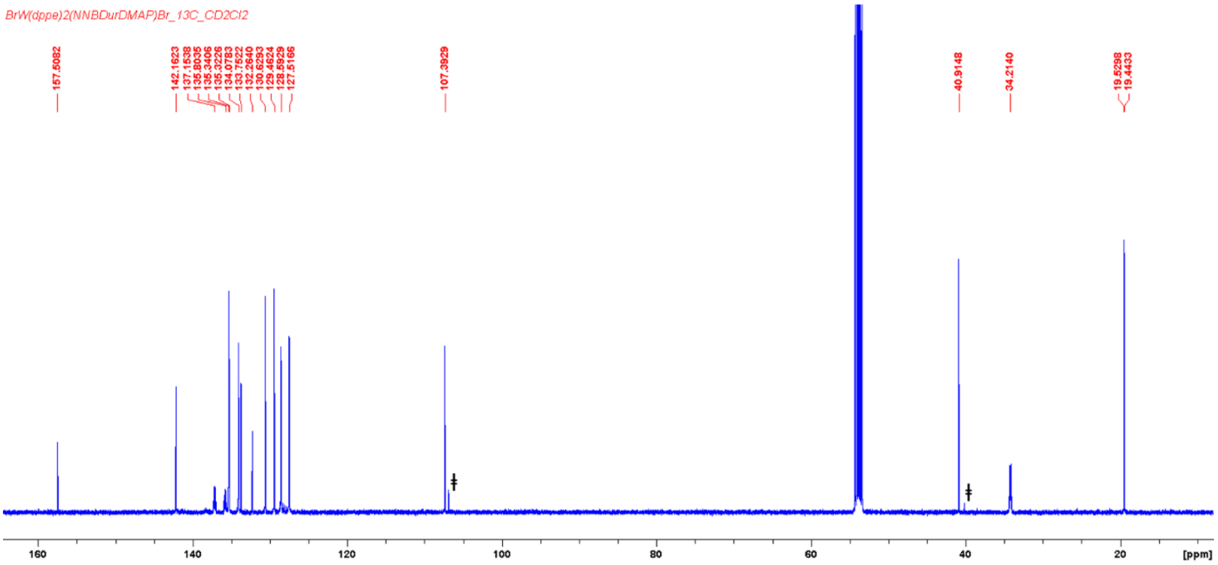


**Figure S84.** $^{13}C\{^{1}H\}$ NMR spectrum of **2k** in $CD_2Cl_2$. Resonances marked with ǂ correspond to residual DMAP.

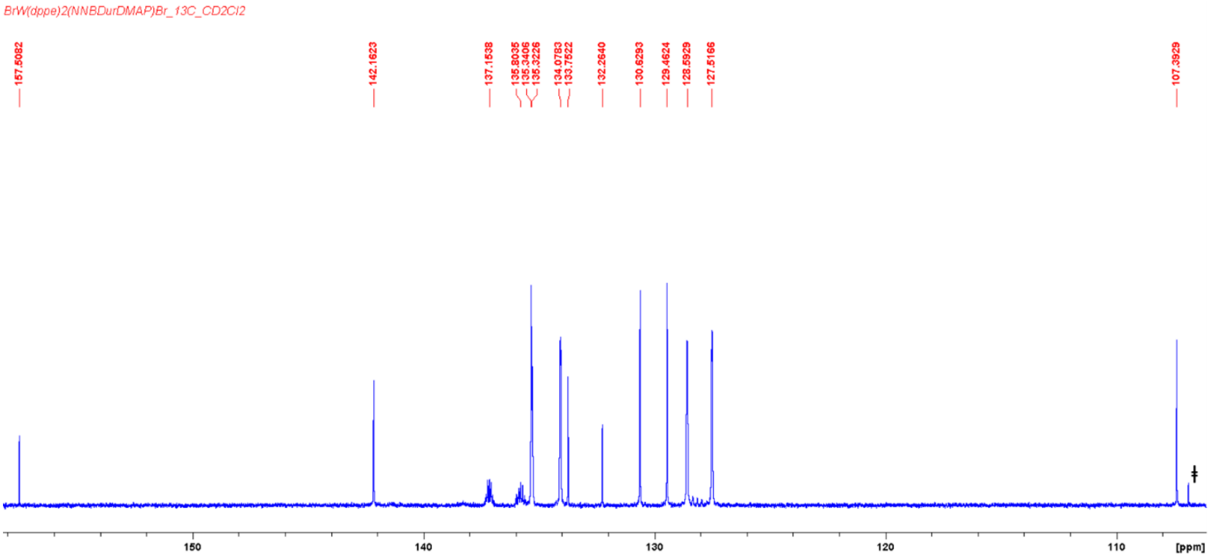


**Figure S85.** Expanded aromatic region of $^{13}C\{^{1}H\}$ NMR spectrum of **2k** in $CD_2Cl_2$. Resonances marked with ǂ correspond to residual DMAP.

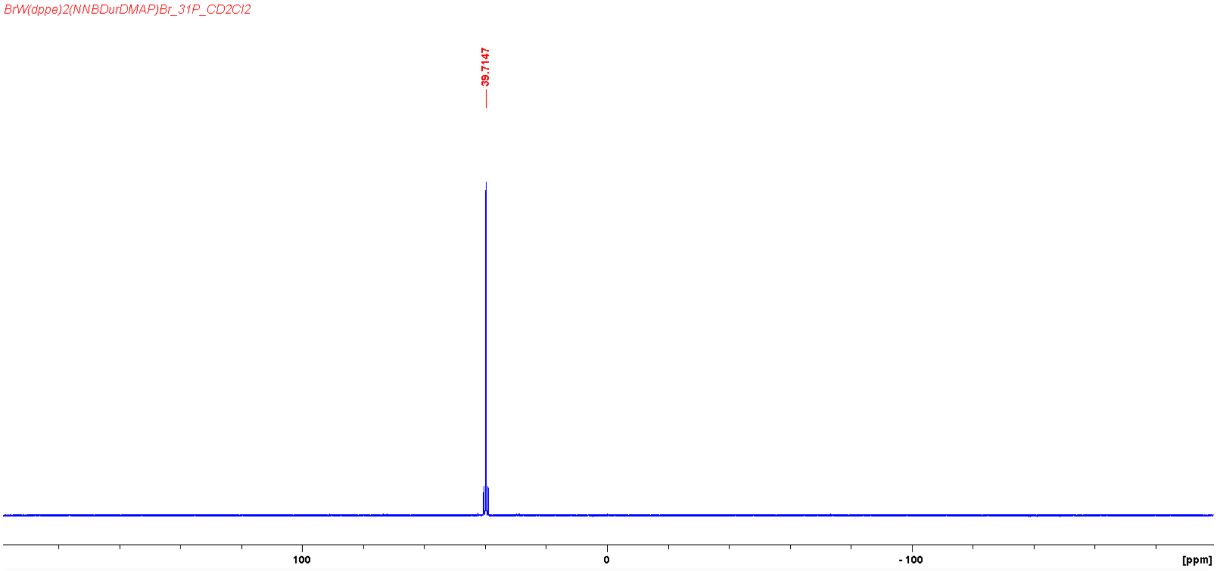


**Figure S86.** $^{31}P\{^{1}H\}$ NMR spectrum of **2k** in $CD_2Cl_2$.

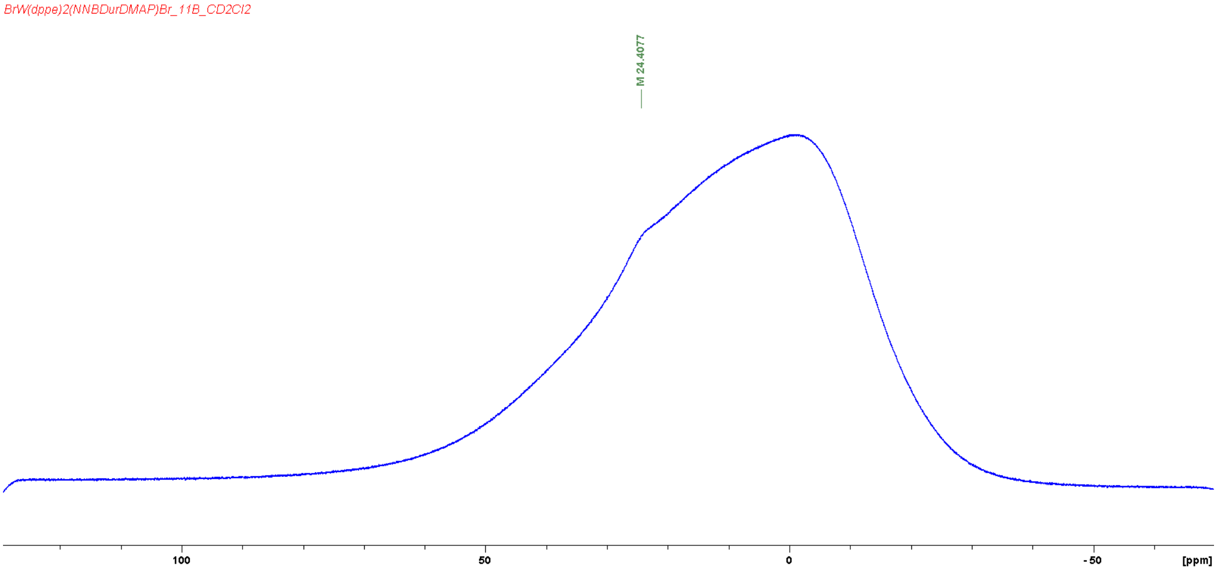


**Figure S87.** $^{11}B$ NMR spectrum of **2k** in $CD_2Cl_2$.

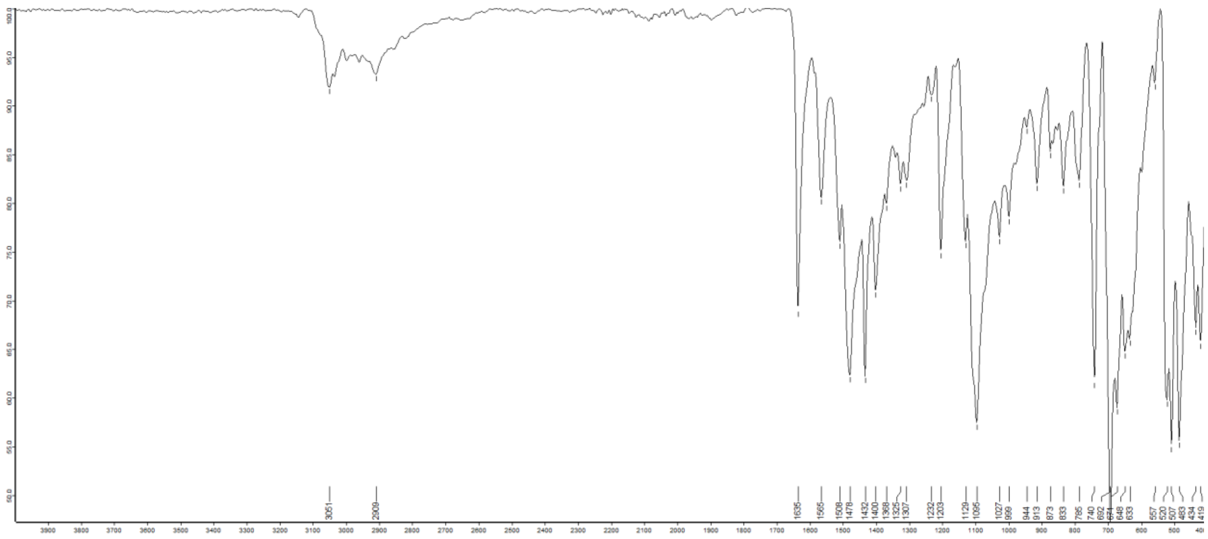


**Figure S88.** Solid-state IR spectrum of **2k**.

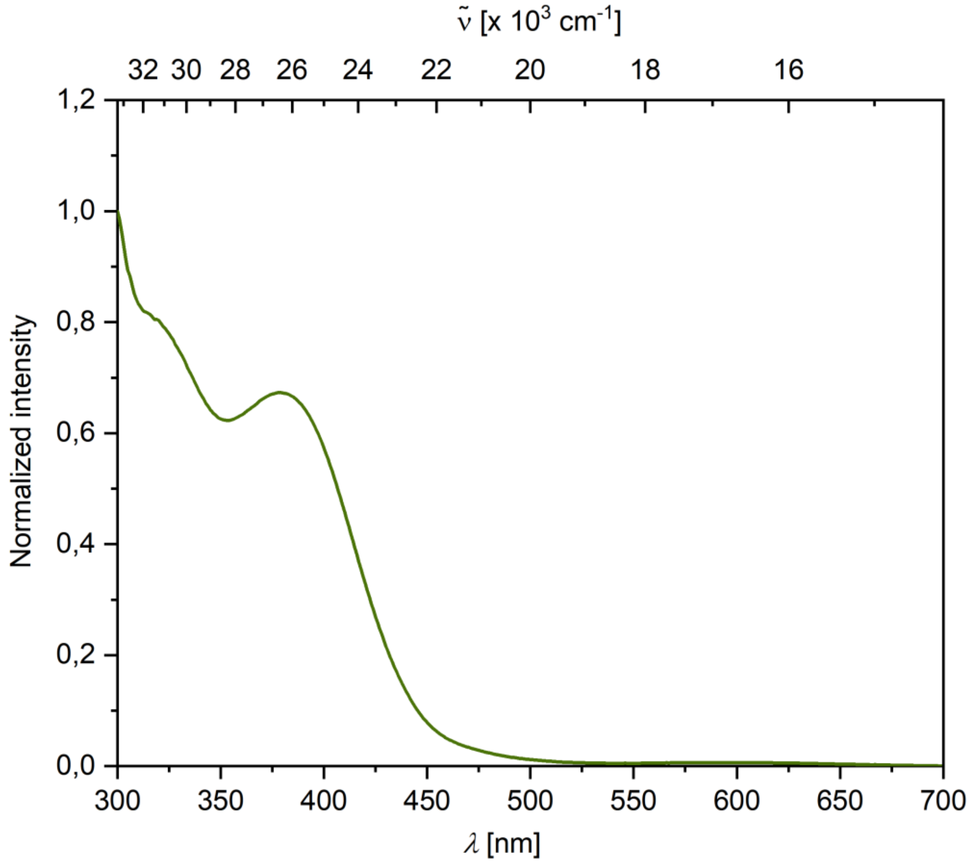


**Figure S89.** UV/Vis spectrum of **2k** in $CD_2Cl_2$.

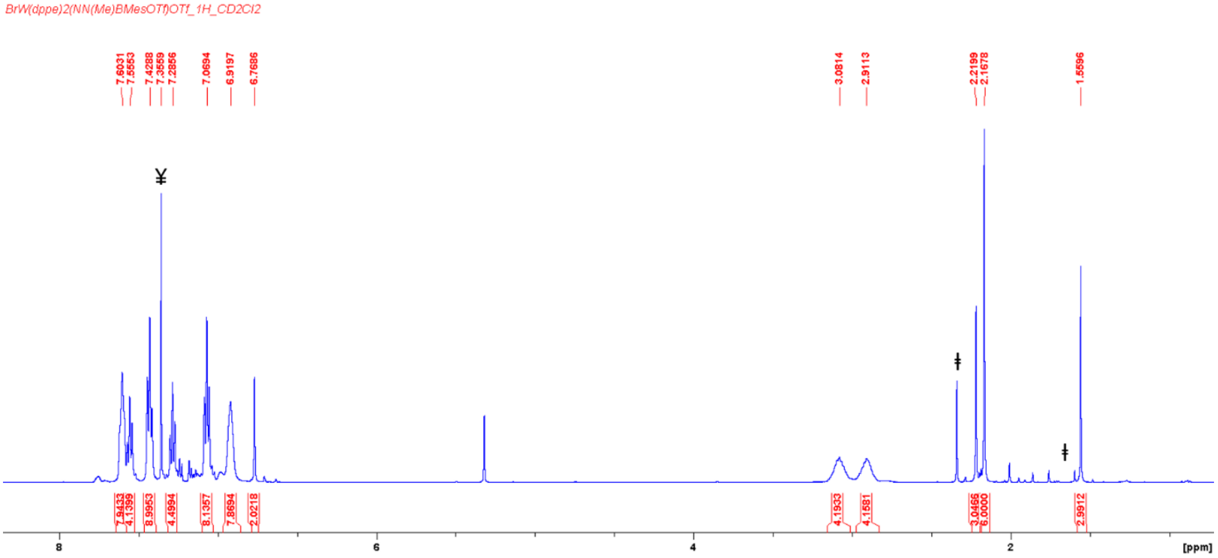


**Figure S90.** $^{1}H$ NMR spectrum of **2l** in $CD_2Cl_2$. Marked resonances correspond to residual toluene (ǂ) and benzene (¥).

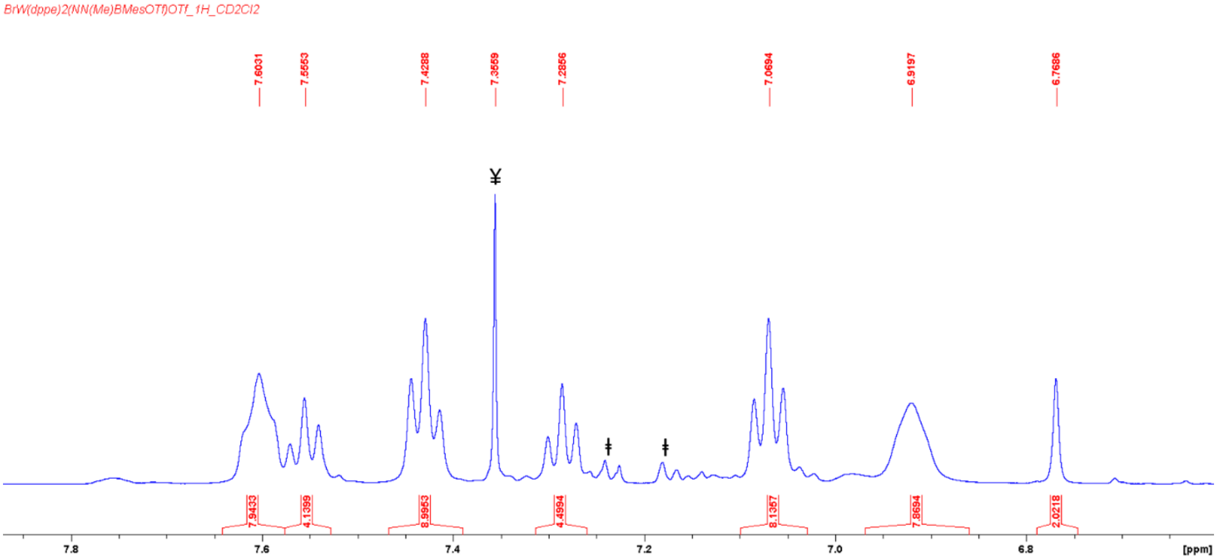


**Figure S91.** Expanded aromatic region of $^{1}H$ NMR spectrum of **2l** in $CD_2Cl_2$. Marked resonances correspond to residual toluene (ǂ) and benzene (¥).

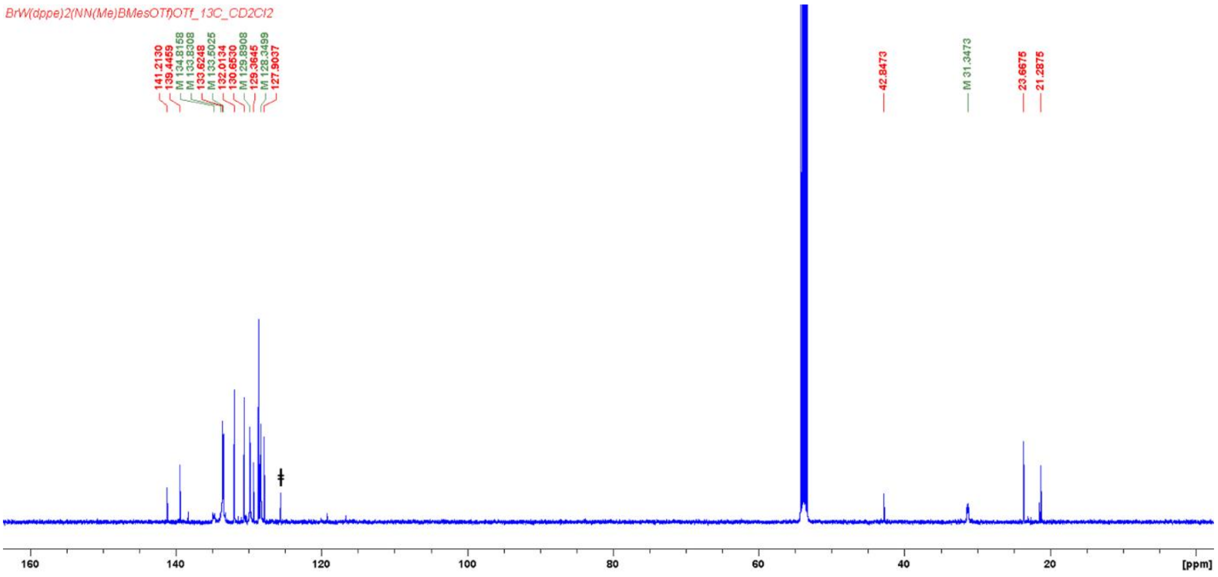


**Figure S92.** $^{13}C\{^1H\}$ NMR spectrum of **2l** in $CD_2Cl_2$. Resonances marked with ǂ correspond to residual toluene.

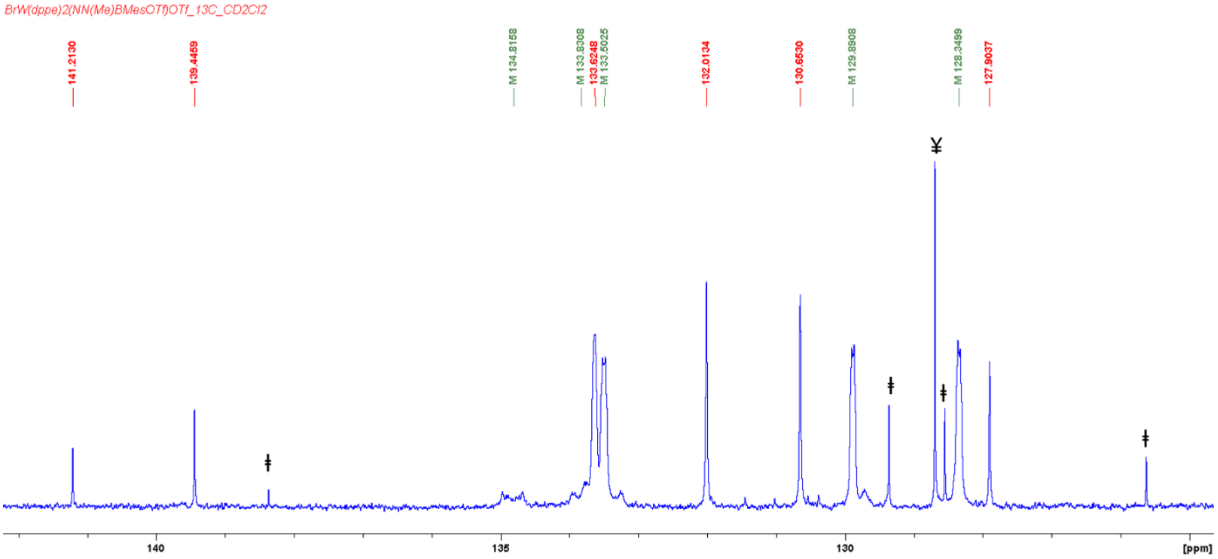


**Figure S93.** Expanded aromatic region of $^{13}C\{^1H\}$ NMR spectrum of **2l** in $CD_2Cl_2$. Marked resonances correspond to residual toluene (ǂ) and benzene (¥).

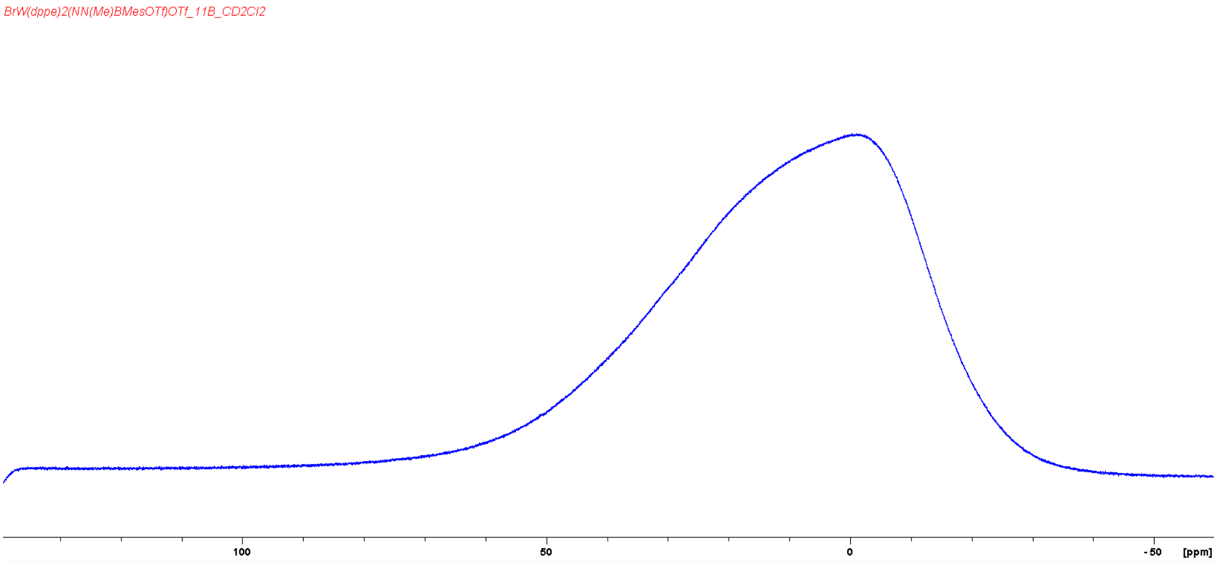


**Figure S94.** $^{11}B$ NMR spectrum of **2l** in $CD_2Cl_2$.

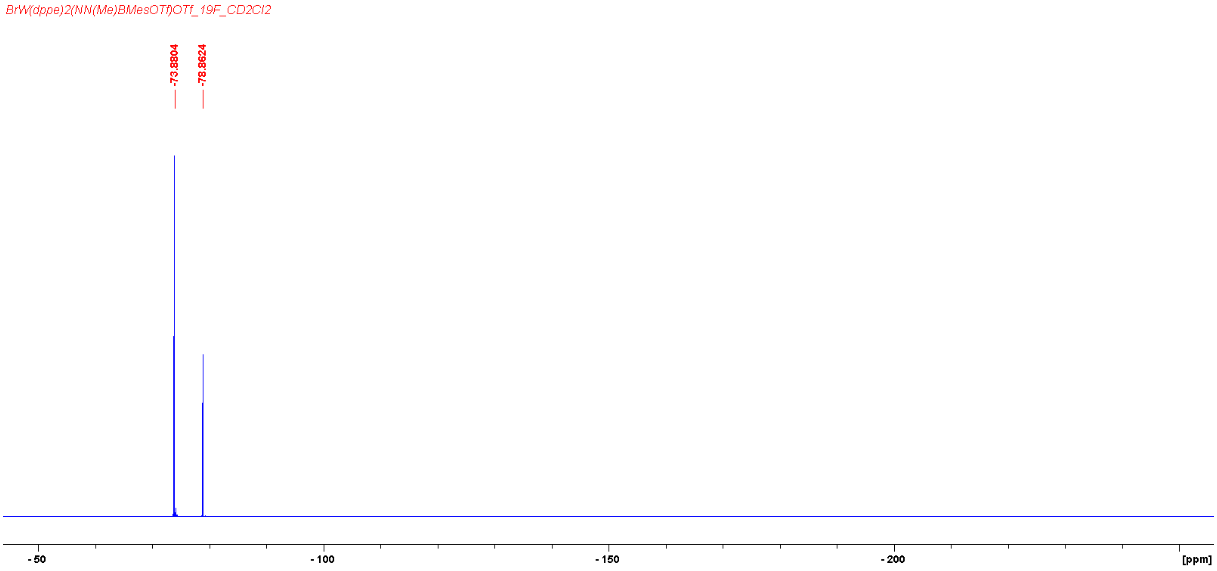


**Figure S95.** $^{19}F$ NMR spectrum of **2l** in $CD_2Cl_2$.

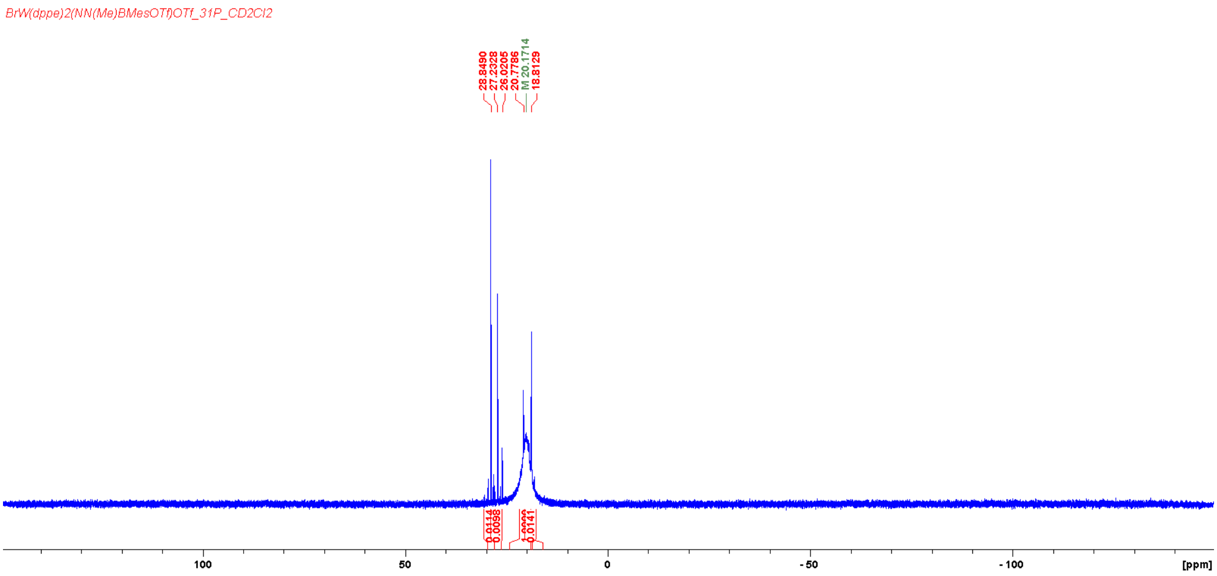


**Figure S96.** $^{31}P\{^{1}H\}$ NMR spectrum of **2l** in $CD_2Cl_2$ at rt.

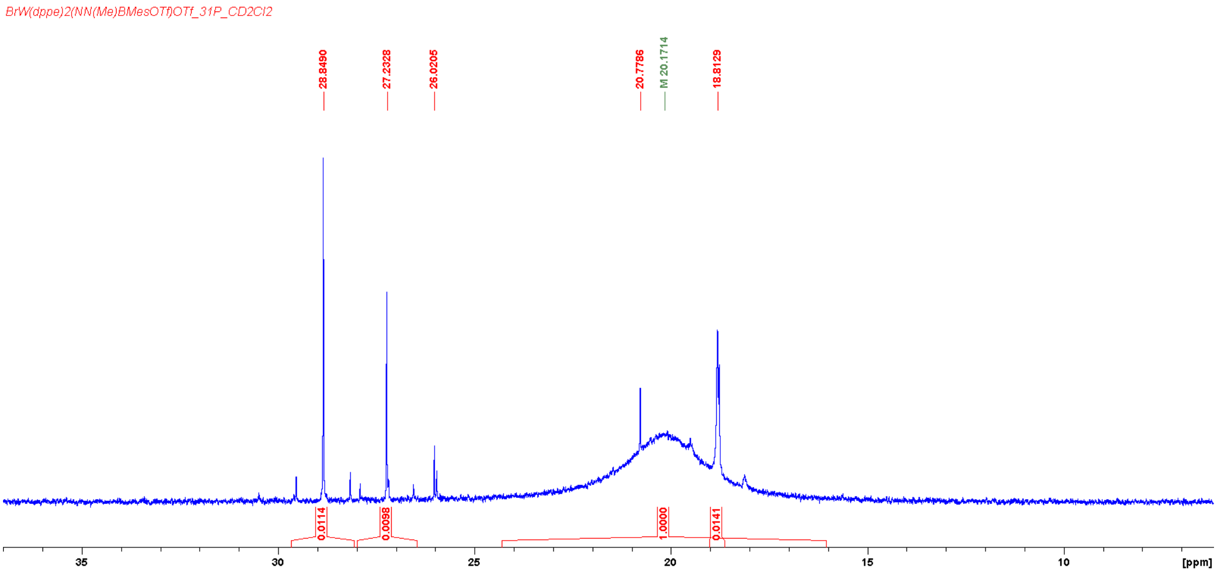


**Figure S97.** Expanded region of $^{31}P\{^{1}H\}$ NMR spectrum of **2l** in $CD_2Cl_2$ at rt..

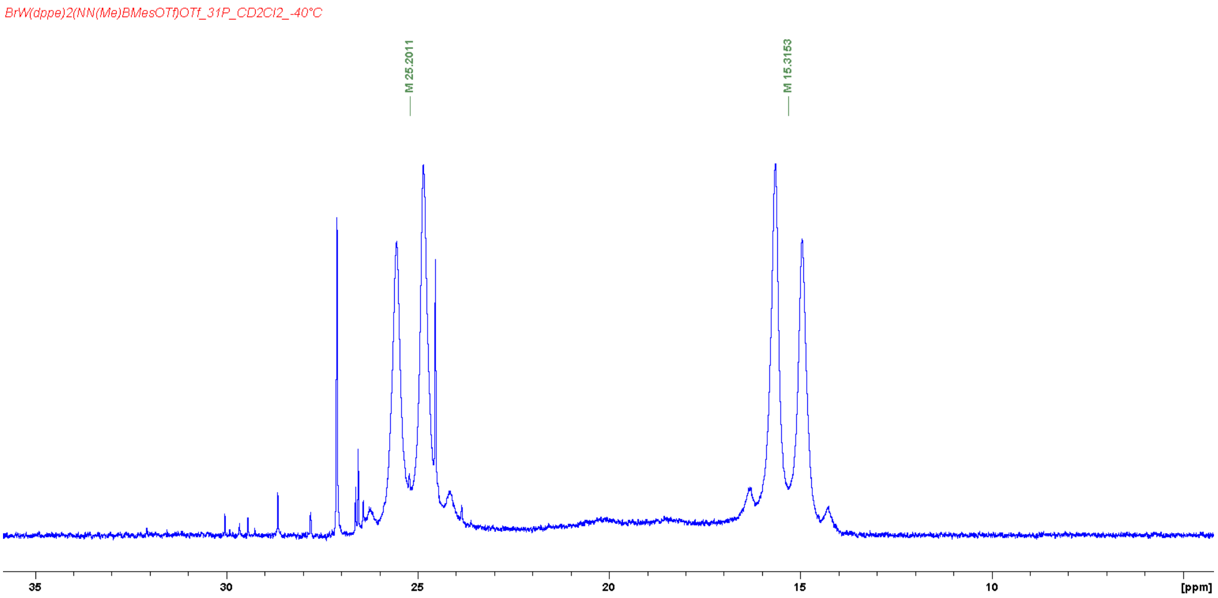


**Figure S98.** $^{31}P\{^{1}H\}$ NMR spectrum of **2l** in $CD_2Cl_2$ at 233 K.

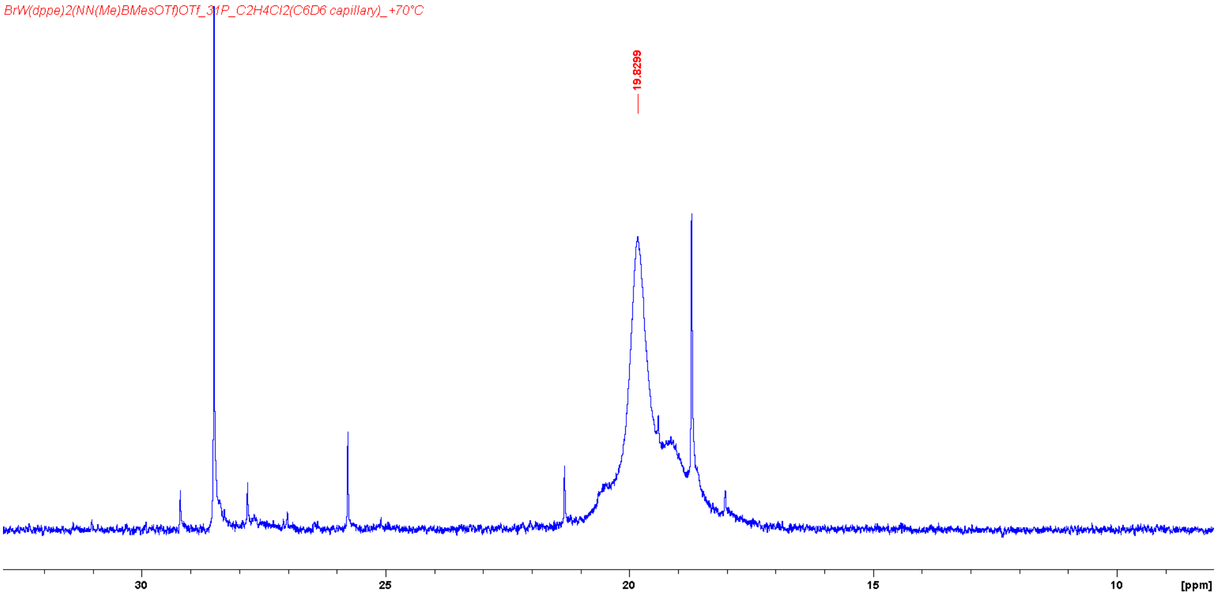


**Figure S99.** $^{31}P\{^{1}H\}$ NMR spectrum of **2l** in $C_2H_4Cl_2$ with a $C_6D_6$ capillary at 343 K.

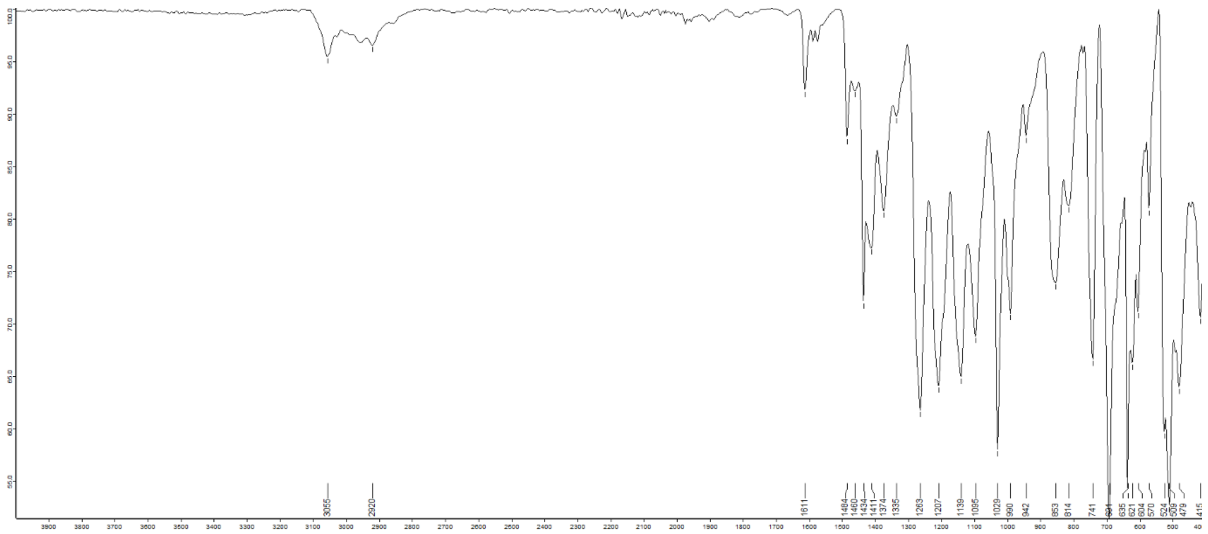

**Figure S100.** Solid-state IR spectrum of **2l**.

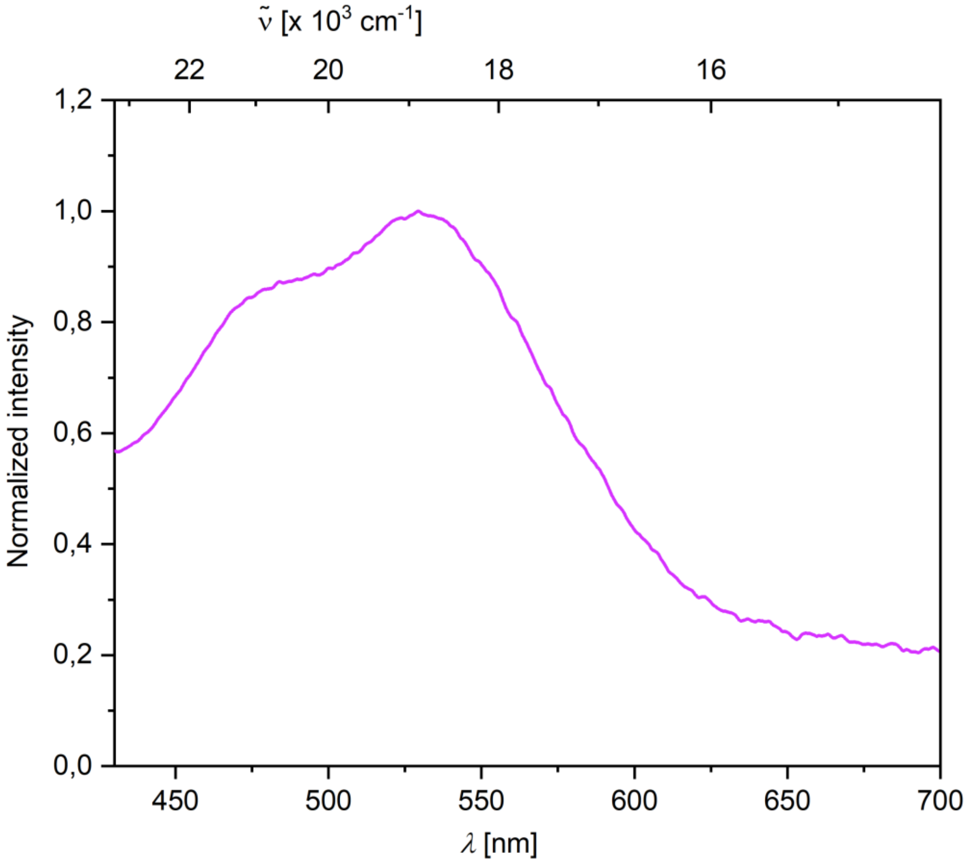


**Figure S101.** UV/Vis spectrum of **2l** in $CH_2Cl_2$.

## X-ray Crystallographic Details:

The crystal data of **2a**, **2b**, **2c**, **2f**, **2h**, **2i**, and **2l** were collected on a BRUKER D8 QUEST diffractometer with a CMOS area detector and multi-layer mirror monochromated $Mo_{K\alpha}$ radiation. The crystal data of **2d**, **2e**, and **2g** were collected on a BRUKER X8-APEX II diffractometer with a CCD area detector and multi-layer mirror monochromated $Mo_{K\alpha}$ radiation. The crystal data of **2k** were collected on a XTALAB SYNERGY, DUALFLEX, HYPIX diffractometer with a Hybrid Pixel Array Detector and multi-layer mirror monochromated $Mo_{K\alpha}$ radiation. The structures were solved using intrinsic phasing method (SHELXT, G. Sheldrick, *Acta Cryst.*, **2015**, *A71*, 3–8), refined with the SHELXL program (G. Sheldrick, *Acta Cryst.*, **2008**, *A64*, 112–122) and expanded using Fourier techniques. All non-hydrogen atoms were refined anisotropically. Hydrogen atoms were included in structure factor calculations. All hydrogen atoms were assigned to idealized geometric positions. Crystallographic data have been deposited with the Cambridge Crystallographic Data Center as supplementary publication no. CCDC-2005678-2005688. These data can be obtained free of charge from The Cambridge Crystallographic Data Centre *via* www.ccdc.cam.ac.uk/data_request/cif

**Refinement details for 2a**: The displacement parameters of atoms B1, Br2 and N2 of the residues 1 and 111 were constrained to the same value with EADP keyword. The displacement parameters of the benzene of the residue 14 were restrained to the same value with similarity restraint SIMU. The distances between atoms in the benzene of the residue 14 were restrained during refinement to the same value with the SADI restraint. The atomic displacement parameters of of the benzene of the residue 14 were restrained with RIGU keyword in ShelXL input ('enhanced rigid bond' restraint for all bonds in the connectivity list. Standard values of 0.005 for both parameters s1 and s2 were used). **Crystal data for 2a**: $C_{73}H_{69.08}BBr_{3.08}N_2P_4W$, $M_r$ = 1538.67, orange needle, 0.415×0.264×0.17 mm$^3$, triclinic space group $P\bar{1}$, $a$ = 13.769(3) Å, $b$ = 14.490(4) Å, $c$ = 17.089(4) Å, α = 90.105(8)°, β = 99.241(14)°, γ = 106.492(6)°, $V$ = 3222.7(12) Å$^3$, $Z$ = 2, $\rho_{calcd}$ = 1.586 g·cm$^{-3}$, μ = 3.843 mm$^{-1}$, $F$(000) = 1535, $T$ = 100(2) K, $R_1$ = 0.0367, $wR^2$ = 0.0670, 12675 independent reflections [2θ≤52.044°] and 692 parameters.

**Crystal data for 2b:** $C_{61}H_{59}BBr_2N_2P_4W$, $M_r$ = 1298.46, orange block, 0.313×0.238×0.222 mm$^3$, triclinic space group $P\bar{1}$, $a$ = 11.153(3) Å, $b$ = 12.439(3) Å, $c$ = 21.741(5) Å, α = 102.526(7)°, β = 95.375(6)°, γ = 109.821(11)°, $V$ = 2723.7(11) Å$^3$, $Z$ = 2, $\rho_{calcd}$ = 1.583 g·cm$^{-3}$, μ = 3.747 mm$^{-1}$, $F$(000) = 1296, $T$ = 114(2) K, $R_1$ = 0.0313, $wR^2$ = 0.0600, 10697 independent reflections [2θ≤52.044°] and 643 parameters.

**Crystal data for 2c:** $C_{62}H_{61}BBr_2N_2P_4W$, $M_r$ = 1312.48, orange plate, 0.261×0.133×0.056 mm$^3$, triclinic space group $P\bar{1}$, $a$ = 11.1496(19) Å, $b$ = 12.550(4) Å, $c$ = 21.525(7) Å, α = 104.723(15)°, β = 93.737(19)°, γ = 107.980(13)°, $V$ = 2736.9(13) Å$^3$, $Z$ = 2, $\rho_{calcd}$ = 1.593 g·cm$^{-3}$, μ = 3.729 mm$^{-1}$, $F$(000) = 1312, $T$ = 100(2) K, $R_1$ = 0.0381, $wR^2$ = 0.0823, 10781 independent reflections [2θ≤52.044°] and 653 parameters.

**Refinement details for 2d**: The displacement parameters of benzenes of the residue 14, 24 and 15 were restrained to the same value with similarity restraint SIMU. The atomic space parameters of both benzenes were refined isotropically. **Crystal data for 2d:** $C_{71}H_{66}BBr_2FeN_2P_4W$, $M_r$ = 1481.46, red plate, 0.308×0.256×0.184 mm$^3$, monoclinic space group $P2_1/n$, $a$ = 13.298(4) Å, $b$ = 27.792(8) Å, $c$ = 17.229(5) Å, β = 106.29(2)°,

$V$ = 6111(3) Å$^3$, $Z$ = 4, $\rho_{calcd}$ = 1.610 g·cm$^{-3}$, μ = 3.576 mm$^{-1}$, $F$(000) = 2964, $T$ = 103(2) K, $R_1$ = 0.0409, $wR^2$ = 0.0910, 12501 independent reflections [2θ≤52.738°] and 677 parameters.

**Crystal data for 2e:** $C_{61}H_{59}BCl_2N_2P_4W$, $M_r$ = 1209.54, orange block, 0.403×0.136×0.112 mm$^3$, triclinic space group $P\bar{1}$, $a$ = 11.066(3) Å, $b$ = 12.402(2) Å, $c$ = 21.637(4) Å, α = 102.423(16)°, β = 94.77(2)°, γ = 109.528(9)°, $V$ = 2694.2(10) Å$^3$, $Z$ = 2, $\rho_{calcd}$ = 1.491 g·cm$^{-3}$, μ = 2.404 mm$^{-1}$, $F$(000) = 1224, $T$ = 103(2) K, $R_1$ = 0.0302, $wR^2$ = 0.0595, 10628 independent reflections [2θ≤52.044°] and 643 parameters.

**Crystal data for 2f:** $C_{62}H_{61}BCl_2N_2P_4W$, $M_r$ = 1223.56, orange plate, 0.284×0.256×0.128 mm$^3$, triclinic space group $P\bar{1}$, $a$ = 11.0966(18) Å, $b$ = 12.646(3) Å, $c$ = 21.3046(19) Å, α = 104.827(7)°, β = 93.149(12)°, γ = 107.893(9)°, $V$ = 2721.2(7) Å$^3$, $Z$ = 2, $\rho_{calcd}$ = 1.493 g·cm$^{-3}$, μ = 2.381 mm$^{-1}$, $F$(000) = 1240, $T$ = 100(2) K, $R_1$ = 0.0342, $wR^2$ = 0.0797, 10723 independent reflections [2θ≤52.042°] and 653 parameters.

**Refinement details for 2g**: The displacement parameters of the mesityl group (C7 to C15 residue 1) and C1 and C2 of residue 3 were restrained to the same value with similarity restraint SIMU. The atomic displacement parameters of the mesityl group (C7 to C15 residue 1) and C1 and C2 of residue 3 were restrained with RIGU keyword in ShelXL input ('enhanced rigid bond' restraint for all bonds in the connectivity list. Standard values of 0.003 for both parameters s1 and s2 were used). **Crystal data for 2g:** $C_{79}H_{76}BBrN_2P_4W$, $M_r$ = 1451.86, brown plate, 0.591×0.104×0.077 mm$^3$, triclinic space group $P\bar{1}$, $a$ = 10.436(3) Å, $b$ = 17.414(6) Å, $c$ = 18.404(6) Å, α = 80.834(11)°, β = 81.511(10)°, γ = 86.451(11)°, $V$ = 3263.1(19) Å$^3$, $Z$ = 2, $\rho_{calcd}$ = 1.478 g·cm$^{-3}$, μ = 2.528 mm$^{-1}$, $F$(000) = 1476, $T$ = 103(2) K, $R_1$ = 0.0994, $wR^2$ = 0.1283, 12467 independent reflections [2θ≤52.044°] and 736 parameters.

**Crystal data for 2h:** $C_{80}H_{81}BBrN_2P_4W$, $M_r$ = 1468.91, yellow block, 0.131×0.091×0.061 mm$^3$, triclinic space group $P\bar{1}$, $a$ = 12.5723(4) Å, $b$ = 14.8130(6) Å, $c$ = 19.7973(7) Å, α = 76.385(2)°, β = 72.690(2)°, γ = 74.4340(10)°, $V$ = 3342.0(2) Å$^3$, $Z$ = 2, $\rho_{calcd}$ = 1.460 g·cm$^{-3}$, μ = 2.469 mm$^{-1}$, $F$(000) = 1498, $T$ = 100(2) K, $R_1$ = 0.0292, $wR^2$ = 0.0478, 13169 independent reflections [2θ≤52.042°] and 794 parameters.

**Refinement details for 2i**: The unit cell contains zero molecules which have been treated as a diffuse contribution to the overall scattering without specific atom positions by SQUEEZE/PLATON. Squeeze was used (as suggested by checkCIF), because too much accessible volume was left over in the unit cell, which is caused by the high volume of the unit cell, due to the three different disordered molecules that are included. (A.L. Spek, *Acta Cryst.*, **2015**, *C71*, 9–18). The displacement parameters of the phenyl groups (residue 6, 9, 11, 26) were restrained to the same value with similarity restraint SIMU. The distances between the atoms of the benzene molecules (residue 32-39) were restrained during refinement to the same value with the SADI restraint. The atomic displacement parameters of the phenyl groups (residue 6, 9, 11, 26) were restrained with the RIGU keyword in ShelXL input ('enhanced rigid bond' for all bonds in the connectivity list. Standard values of 0.003 for both parameters s1 and s2 were used. **Crystal data for 2i:** $C_{94}H_{94}BBrN_2P_4W$, $M_r$ = 1650.16, brown plate, 0.126×0.113×0.015 mm$^3$, triclinic space group $P\bar{1}$, $a$ = 20.739(3) Å, $b$ = 21.463(3) Å, $c$ = 22.878(3) Å, α = 93.788(5)°, β = 111.353(4)°, γ = 115.216(4)°, $V$ = 8279.9(19) Å$^3$, $Z$ = 4, $\rho_{calcd}$ = 1.324 g·cm$^{-3}$, μ = 2.001 mm$^{-1}$, $F$(000) = 3384, $T$ = 120(2) K, $R_1$ = 0.1061, $wR^2$ = 0.2166, 32521 independent reflections [2θ≤52.044°] and 1556 parameters.

**Crystal data for 2k:** $C_{69}H_{71}BBr_2N_4P_4W$, $M_r$ = 1434.65, brown block, 0.178×0.130×0.097 mm$^3$, monoclinic space group *C 1 2/c 1*, *a* = 21.3271(6) Å, *b* = 22.4443(4) Å, *c* = 25.8929(5) Å, α = 90.0°, β = 90.204(2)°, γ = 90.0°, *V* = 12394.1(5) Å$^3$, *Z* = 8, $\rho_{calcd}$ = 1.538 g·cm$^{-3}$, μ = 3.302 mm$^{-1}$, *F*(000) = 5776 *T* = 101(4) K, $R_1$ = 0.0414, $wR^2$ = 0.0815, 12070 independent reflections [2θ≤52.044°] and 737 parameters.

**Refinement details for 2l**: The unit cell contains 7.5 molecules of toluene, which have been treated as a diffuse contribution to the overall scattering without specific atom positions by SQUEEZE/PLATON (A.L. Spek, *Acta Cryst.*, **2015**, *C71*, 9–18). **Crystal data for 2l:** $C_{64}H_{62}BBrF_6N_2O_6P_4S_2W$, $M_r$ = 1531.72, purple block, 0.296×0.121×0.078 mm$^3$, triclinic space group *P* $\bar{1}$, *a* = 15.7694(6) Å, *b* = 17.1605(6) Å, *c* = 17.3677(6) Å, α = 111.7390(10)°, β = 91.9450(10)°, γ = 105.6570(10)°, *V* = 4156.3(3) Å$^3$, *Z* = 2, $\rho_{calcd}$ = 1.224 g·cm$^{-3}$, μ = 2.051 mm$^{-1}$, *F*(000) = 1536, *T* = 100(2) K, $R_1$ = 0.0505, $wR^2$ = 0.0902, 16391 independent reflections [2θ≤52.044°] and 788 parameters.

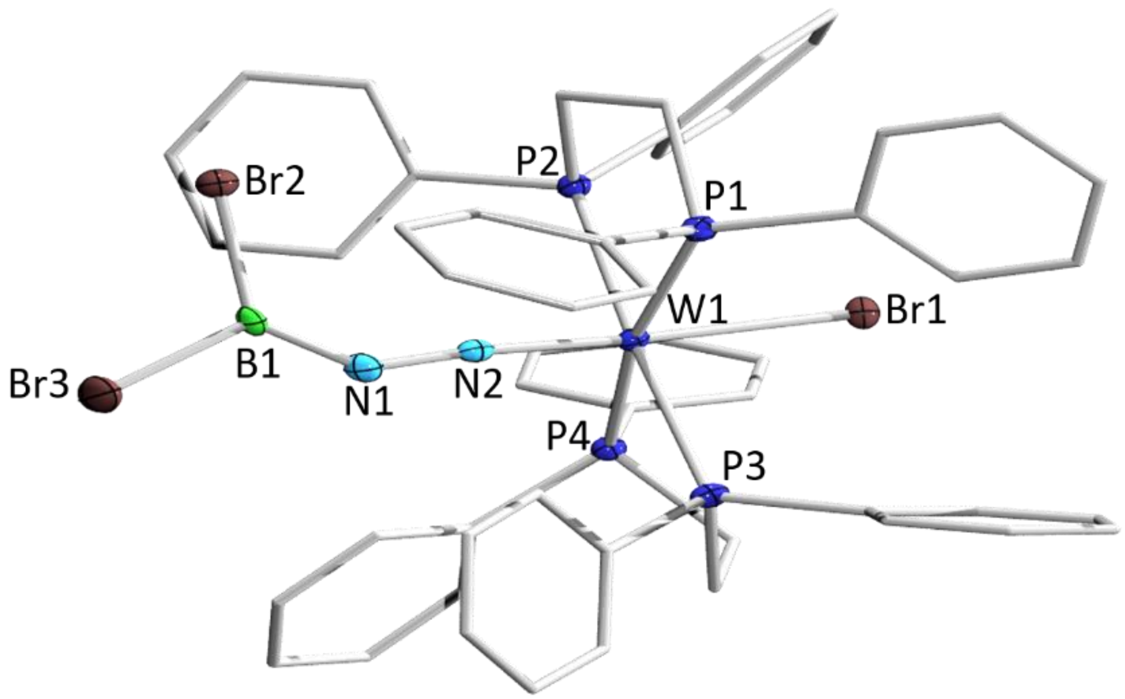


**Figure S100.** Molecular structure of **2a** as determined by single-crystal X-ray diffraction. Hydrogen atoms are omitted for clarity and thermal ellipsoids are set at 50% probability. Selected bond lengths [Å] and angles [°]: W1−N2 1.786(3), N1−N2 1.263(5), B1−N1 1.351(7), B1-N1-N2 138.8(4).

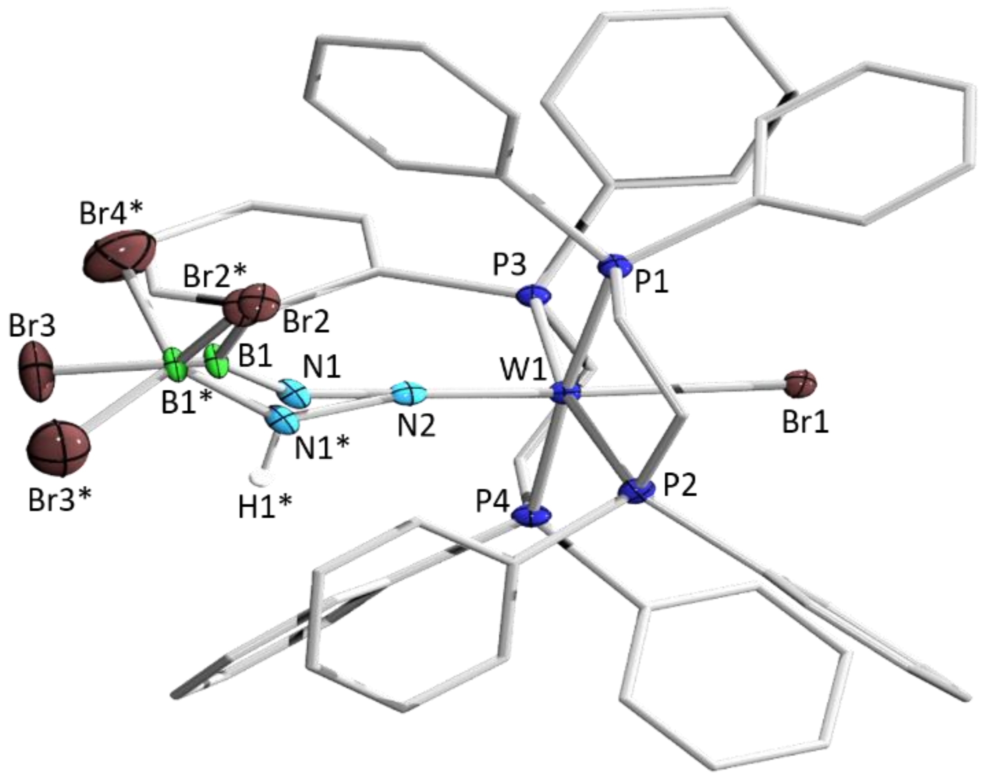


**Figure S101.** Molecular structure of **2a** and **2a’** as determined by single-crystal X-ray diffraction.

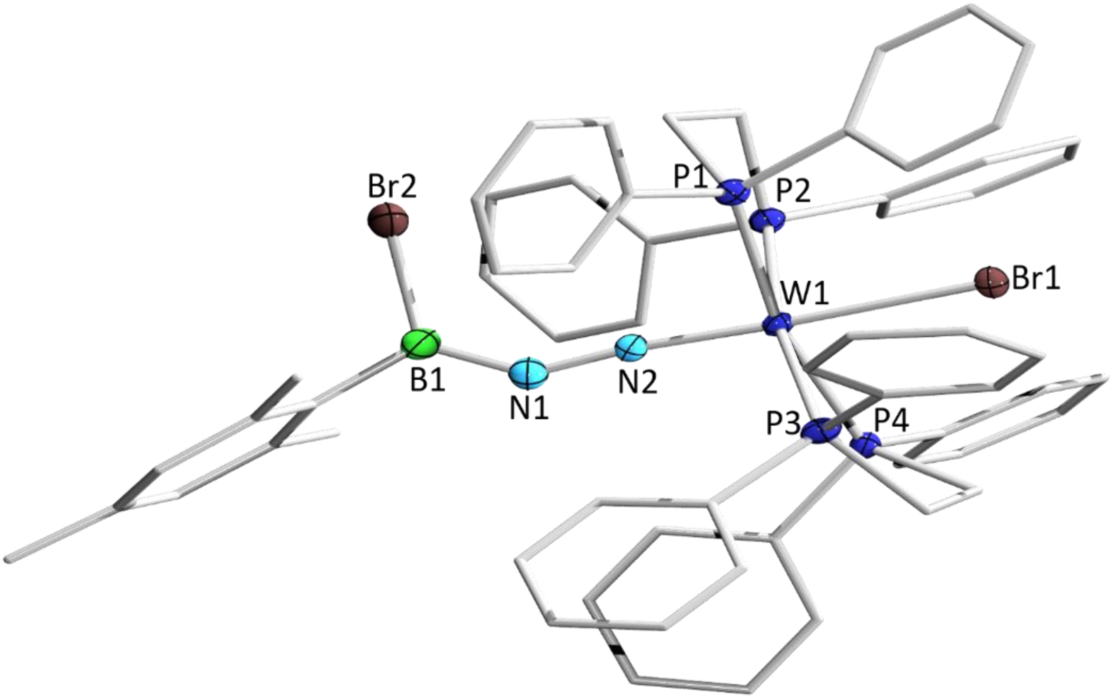


**Figure S102.** Molecular structure of **2b** as determined by single-crystal X-ray diffraction. Hydrogen atoms are omitted for clarity and thermal ellipsoids are set at 50% probability. Selected bond lengths [Å] and angles [°]: W1−N2 1.780(2), N1−N2 1.279(4), B1−N1 1.356(5), B1-N1-N2 141.5(3).

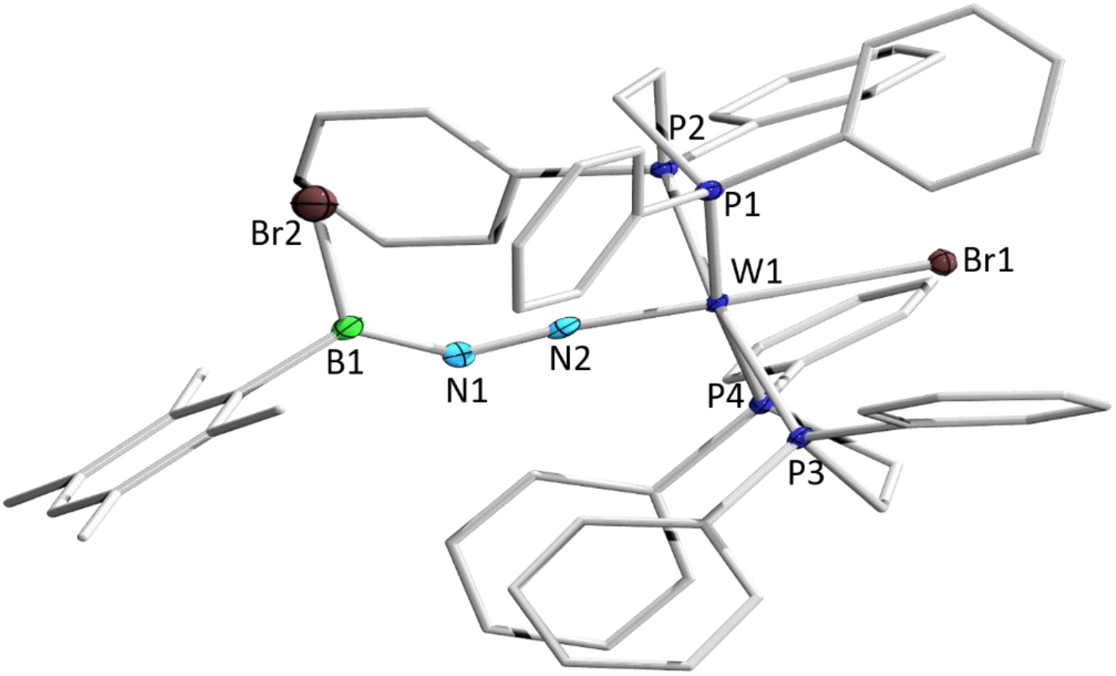


**Figure S103.** Molecular structure of **2c** as determined by single-crystal X-ray diffraction. Hydrogen atoms are omitted for clarity and thermal ellipsoids are set at 50% probability. Selected bond lengths [Å] and angles [°]: W1−N2 1.792(3), N1−N2 1.252(5), B1−N1 1.375(6), B1-N1-N2 142.2(4).

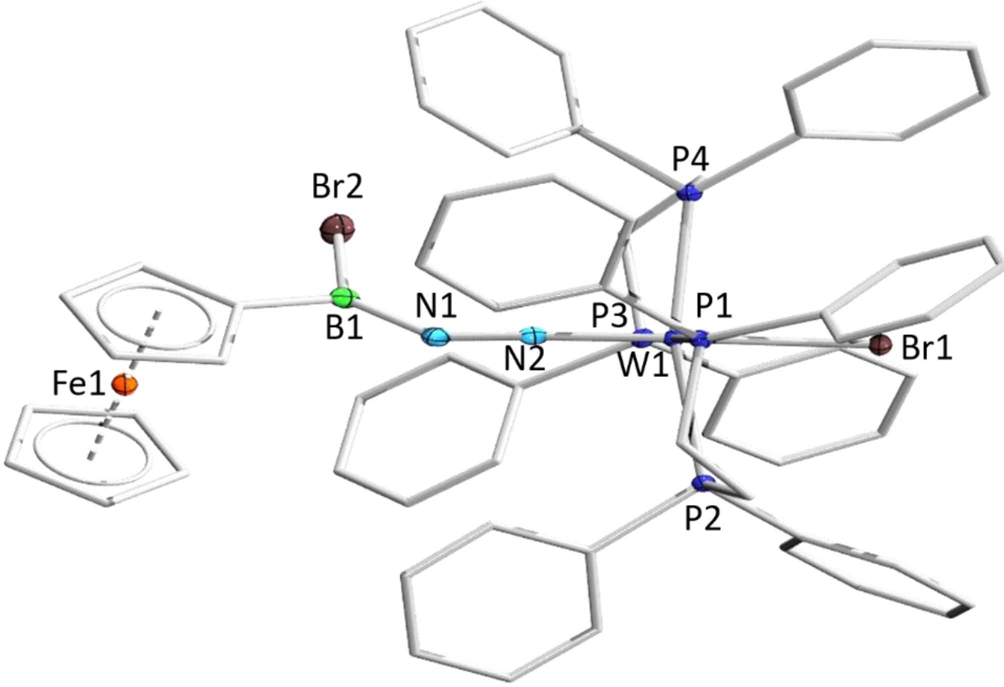


**Figure S104.** Molecular structure of **2d** as determined by single-crystal X-ray diffraction. Hydrogen atoms are omitted for clarity and thermal ellipsoids are set at 50% probability. Selected bond lengths [Å] and angles [°]: W1−N2 1.793(4), N1−N2 1.256(6), B1−N1 1.381(7), B1-N1-N2 138.6(4).

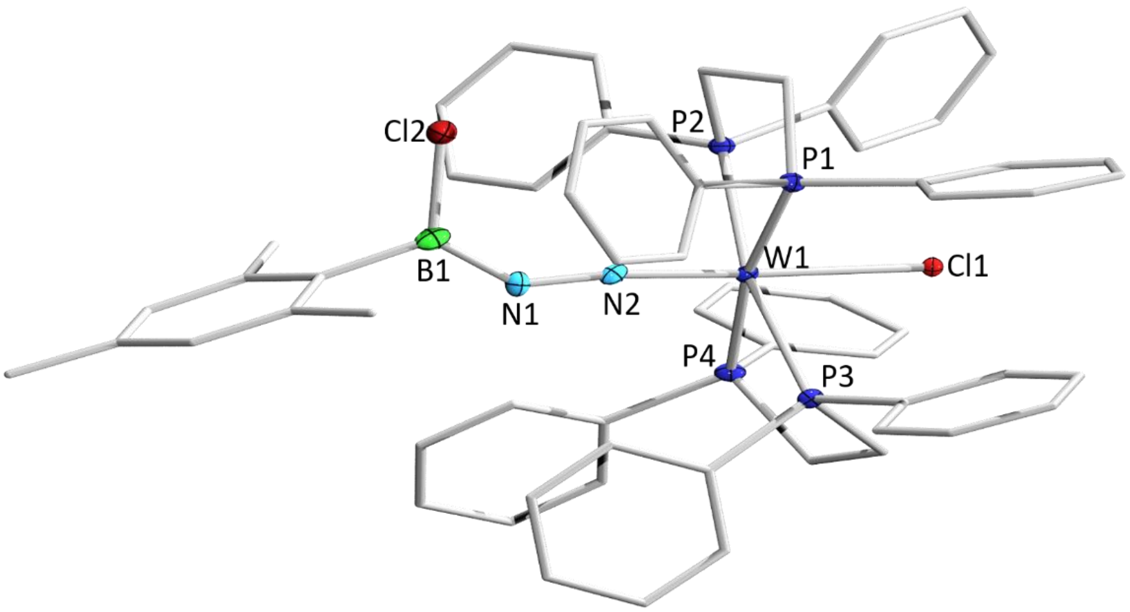


**Figure S105.** Molecular structure of **2e** as determined by single-crystal X-ray diffraction. Hydrogen atoms are omitted for clarity and thermal ellipsoids are set at 50% probability. Selected bond lengths [Å] and angles [°]: W1−N2 1.783(2), N1−N2 1.284(3), B1−N1 1.361(4), B1-N1-N2 138.2(3).

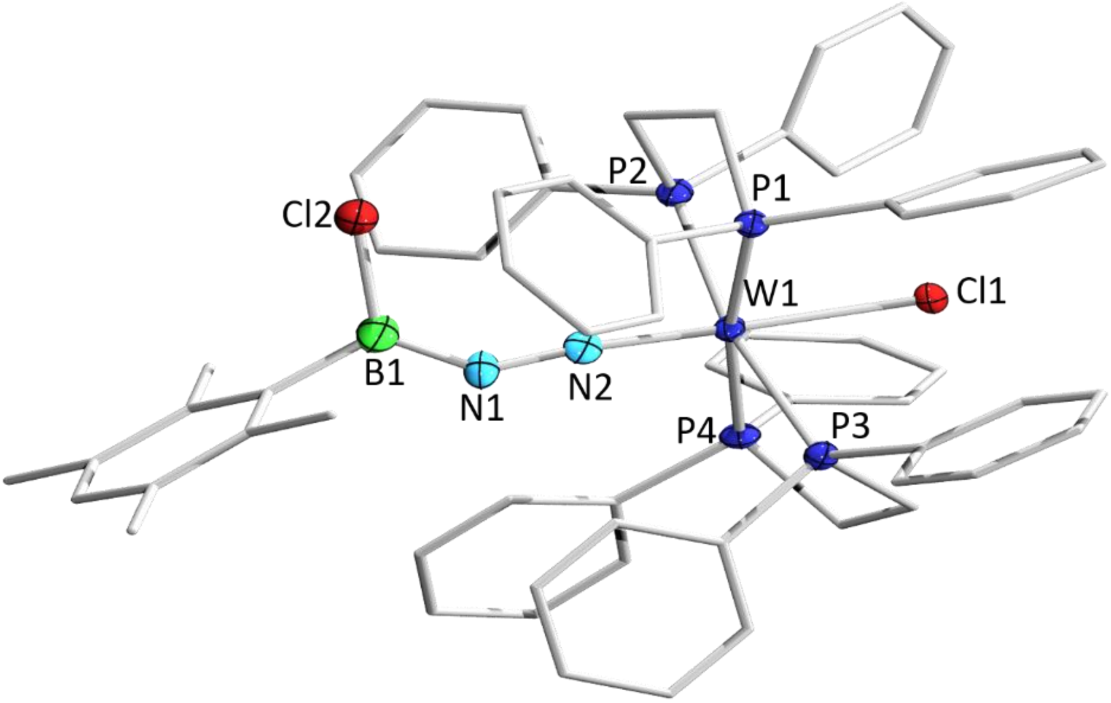


**Figure S106.** Molecular structure of **2f** as determined by single-crystal X-ray diffraction. Hydrogen atoms are omitted for clarity and thermal ellipsoids are set at 50% probability. Selected bond lengths [Å] and angles [°]: W1−N2 1.793(4), N1−N2 1.269(5), B1−N1 1.363(6), B1-N1-N2 139.9(4).

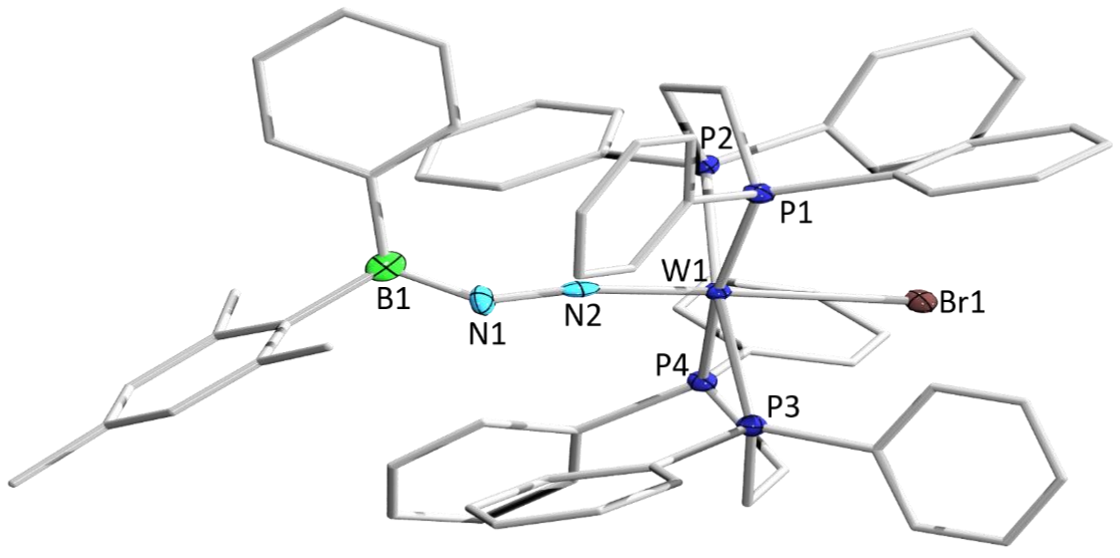


**Figure S107.** Molecular structure of **2g** as determined by single-crystal X-ray diffraction. Hydrogen atoms are omitted for clarity and thermal ellipsoids are set at 50% probability. Selected bond lengths [Å] and angles [°]: W1−N2 1.797(5), N1−N2 1.280(7), B1−N1 1.391(10), B1-N1-N2 146.9(7).

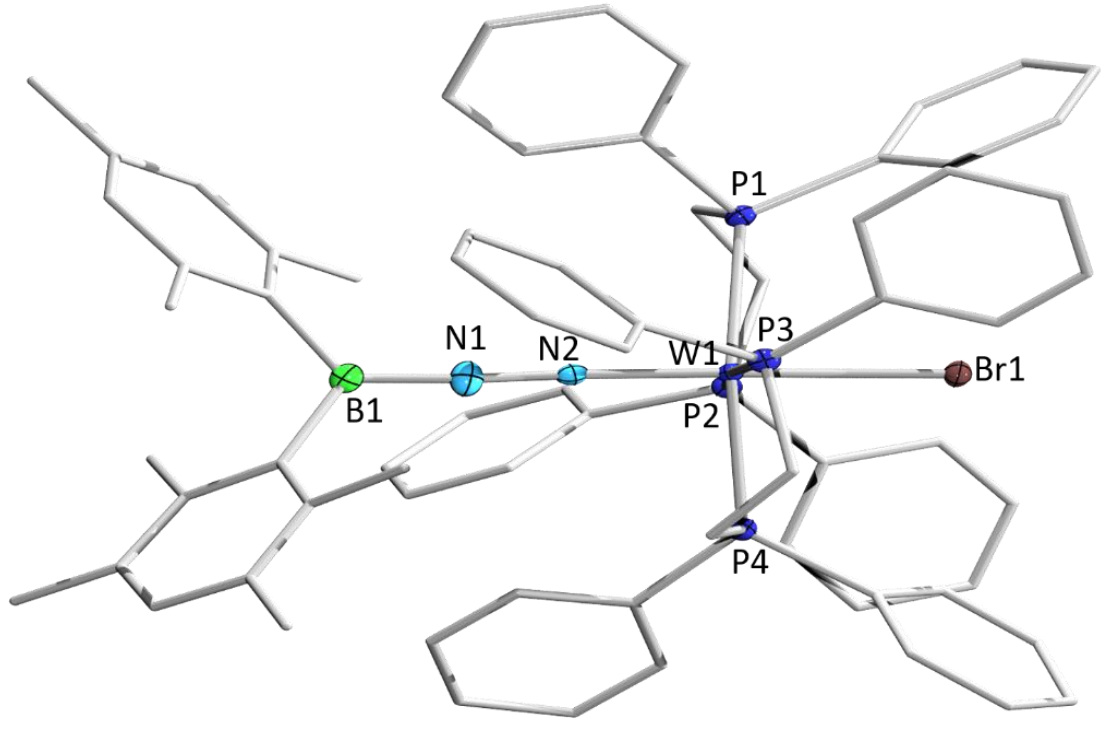


**Figure S108.** Molecular structure of **2h** as determined by single-crystal X-ray diffraction. Hydrogen atoms are omitted for clarity and thermal ellipsoids are set at 50% probability. Selected bond lengths [Å] and angles [°]: W1−N2 1.8353(19), N1−N2 1.226(3), B1−N1 1.392(3), B1-N1-N2 177.40(18).

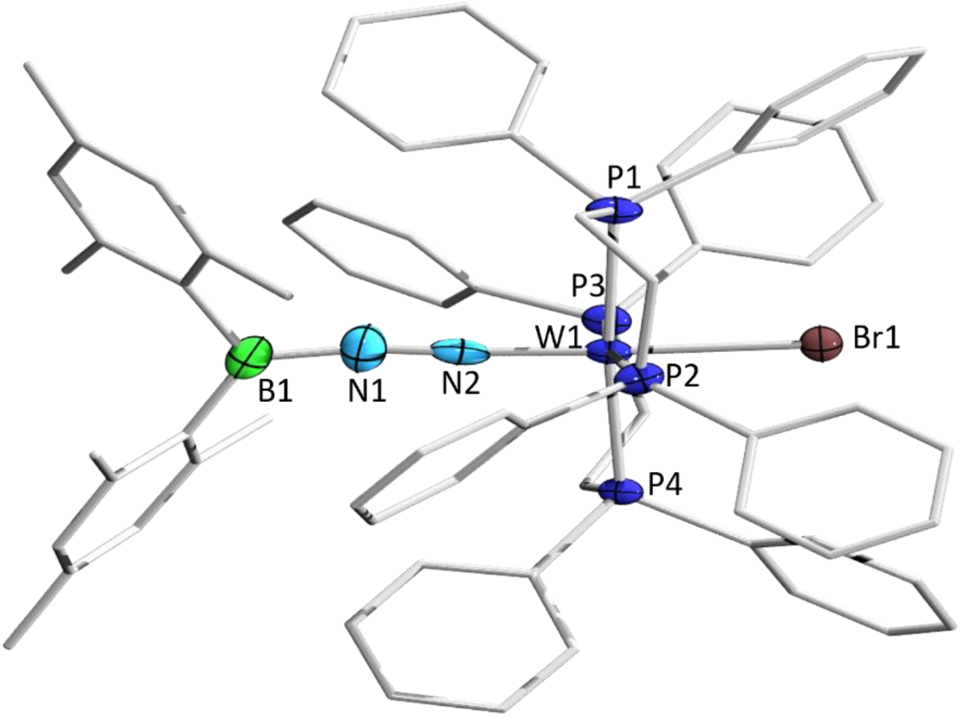


**Figure S109.** Molecular structure of **2i** as determined by single-crystal X-ray diffraction. Hydrogen atoms are omitted for clarity and thermal ellipsoids are set at 50% probability. Selected bond lengths [Å] and angles [°]: W1−N2 1.835(9), N1−N2 1.200(12), B1−N1 1.420(15), B1-N1-N2 169.9(12).

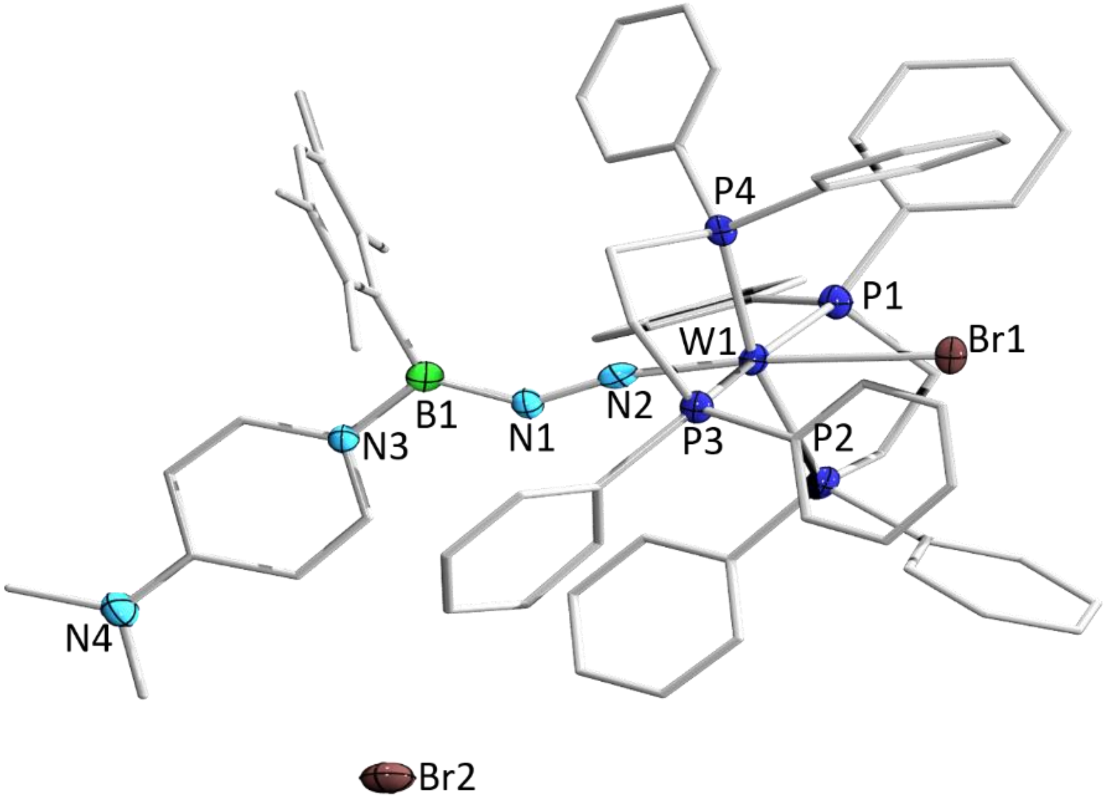


**Figure S110.** Molecular structure of **2k** as determined by single-crystal X-ray diffraction. Hydrogen atoms are omitted for clarity and thermal ellipsoids are set at 50% probability. Selected bond lengths [Å] and angles [°]: W1−N2 1.797(3), N1−N2 1.283(5), B1−N1 1.377(6), B1−N3 1.543(6), B1-N1-N2 135.0(4).

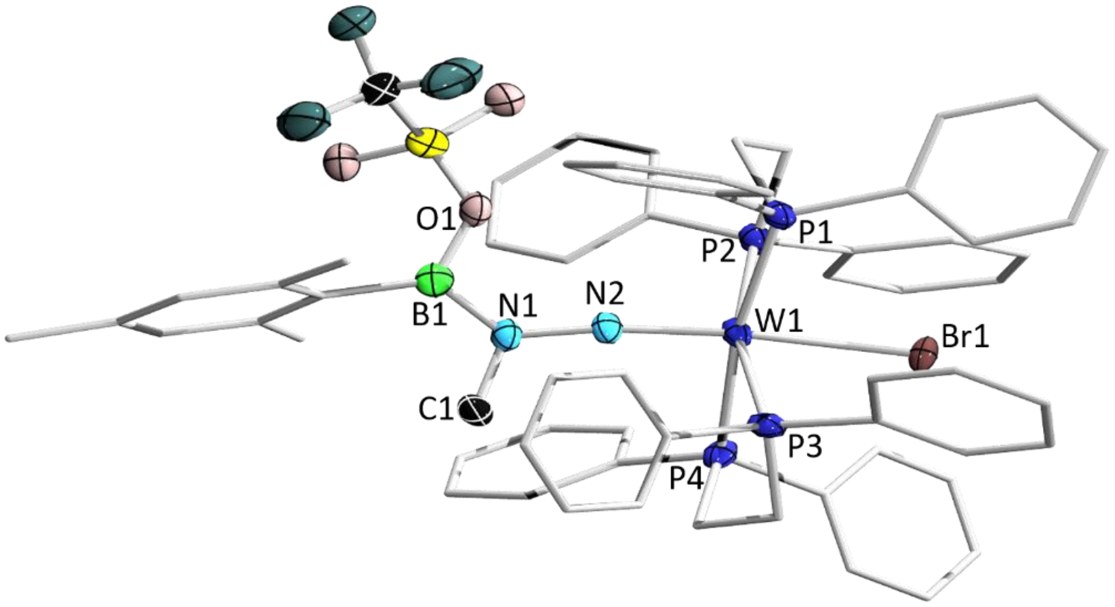


**Figure S110.** Molecular structure of **2l** as determined by single-crystal X-ray diffraction. Hydrogen atoms and the counterion are omitted for clarity and thermal ellipsoids are set at 50% probability. Selected bond lengths [Å] and angles [°]: W1−N2 1.766(3), N1−N2 1.380(4), B1−N1 1.402(6), N1−C1 1.491(5), B1-N1-N2 126.1(3).

## Computational Details

Initially, we performed geometry optimizations and Hessian calculations at the density functional theory level for compounds **1**, **2b**, **2f** and **2k** starting from their crystal structures. We employed the B3LYP[1] functional with D3[2] dispersion corrections and the Becke-Johnson[3] damping function, leading to B3LYP-D3(BJ),[4] in conjunction with the def2-SVP[5] basis sets for all non-metal atoms. Tungsten was described using the Stuttgart-Dresden SDD basis set[6] and effective core potential (ECP),[7] where its 60 core electrons were replaced by the ECP. All structures were characterized as minimum energy geometries as only real frequencies were observed in their vibrational analyses.

At the optimized structures, single-point calculations at the B3LYP-D3(BJ)/TZ2P level of theory were performed, followed by energy decomposition analysis in conjunction with natural orbitals for chemical valence (EDA-NOCV)[8] calculations. Scalar relativistic effects were taken into account by applying the zeroth order regular approximation (ZORA).[9] The EDA-NOCV calculations were used to investigate the W–$N_2$ bonding in **1**, **2b** and **2f**, as well as the B–DMAP bonding in **2k**. For **1**, neutral [$N_2$W] and $N_2$ fragments were used in the analysis. In turn, charged fragments were used in **2b** and **2f**, where the combination of $[XW]^-$ + $[N_2BXAr]^+$ was the only that allowed direct comparison with **1**. Due to the use of charged fragments, the total interaction energy ($\Delta E_{int}$) is much larger in **2b** (−323.7 kcal mol$^{-1}$) and **2f** (−317.9 kcal mol$^{-1}$) than in **1** (−48.4 kcal mol$^{-1}$). Finally, the B–DMAP bonding in **2k** was investigated considering $[BrWN_2BDur]^+$ and neutral DMAP fragments. In addition to the EDA-NOCV calculations, we also obtained Mayer bond orders (MBOs)[10] at the same level of theory, namely B3LYP-D3(BJ)/ZORA/TZ2P.

All geometry optimizations and Hessian calculations were performed with the Gaussian 16, Revision B.01 program.[11] The EDA-NOCV and MBO analyses were carried out using the ADF 2018 software.[12] Plots of the NOCV deformation densities were obtained using Gaussview.

**Table S1.** EDA-NOCV results for **1**. Energies are given in kcal mol$^{-1}$.

| Energy Terms | Orbital Interaction | Energy $[N_2W] + N_2$ | NOCV Eigenvalue, $\nu_k$ |
|---|---|---|---|
| $\Delta E_{int}$ | - | −48.4 | - |
| $\Delta E_{Pauli}$ | - | 111.3 | - |
| $\Delta E_{disp}$[*] | - | −13.0 (8.1%) | |
| $\Delta E_{elstat}$[*] | - | −67.0 (42.0%) | - |
| $\Delta E_{orb}$[*] | - | −79.6 (49.9%) | - |
| $\Delta E_{orb(1)}$[†] | $W \rightarrow N_2$ ($\pi_I$) | −27.0 (33.8%) | 0.65 |
| $\Delta E_{orb(2)}$[†] | $W \rightarrow N_2$ ($\pi_\perp$) | −23.8 (29.9%) | 0.59 |
| $\Delta E_{orb(3)}$[†] | $N_2 \rightarrow W$ ($\sigma$) | −23.5 (29.5%) | 0.37 |
| $\Delta E_{orb(rest)}$[†] | - | −5.4 (6.8%) | - |

[*]The values in parentheses show the weight of each contribution with respect to the total attractive interaction.
[†]The values in parentheses show the weight of each contribution with respect to the total orbital interaction, $\Delta E_{orb}$.

**Table S2.** EDA-NOCV results for **2b**. Energies are given in kcal mol$^{-1}$.

| Energy Terms | Orbital Interaction | Energy $[XW]^{-}$ + $[N2BXAr]^{+}$ | NOCV Eigenvalue, $\nu_k$ |
|---|---|---|---|
| $\Delta E_{int}$ | - | −323.7 | - |
| $\Delta E_{Pauli}$ | - | 246.0 | - |
| $\Delta E_{disp}$[*] | - | −33.5 (5.9%) | |
| $\Delta E_{elstat}$[*] | - | −159.5 (28.0%) | - |
| $\Delta E_{orb}$[*] | - | −376.8 (66.1%) | - |
| $\Delta E_{orb(1)}$[†] | $W \rightarrow N_2$ ($\pi_I$) | −171.9 (45.6%) | 1.55 |
| $\Delta E_{orb(2)}$[†] | $W \rightarrow N_2$ ($\pi_\perp$) | −138.1 (36.7%) | 1.27 |
| $\Delta E_{orb(3)}$[†] | $N_2 \rightarrow W$ ($\sigma$) | −36.0 (9.6%) | 0.55 |
| $\Delta E_{orb(rest)}$[†] | - | −30.8 (8.2%) | - |

[*]The values in parentheses show the weight of each contribution with respect to the total attractive interaction.
[†]The values in parentheses show the weight of each contribution with respect to the total orbital interaction, $\Delta E_{orb}$.

**Table S3.** EDA-NOCV results for **2f**. Energies are given in kcal mol$^{-1}$.

| Energy Terms | Orbital Interaction | Energy $[XW]^{-}$ + $[N2BXAr]^{+}$ | NOCV Eigenvalue, $\nu_k$ |
|---|---|---|---|
| $\Delta E_{int}$ | - | −317.9 | - |
| $\Delta E_{Pauli}$ | - | 245.3 | - |
| $\Delta E_{disp}$[*] | - | −34.4 (6.1%) | |
| $\Delta E_{elstat}$[*] | - | −161.9 (28.7%) | - |
| $\Delta E_{orb}$[*] | - | −366.9 (65.2%) | - |
| $\Delta E_{orb(1)}$[†] | W→$N_2$ ($\pi_I$) | −175.3 (47.8%) | 1.57 |
| $\Delta E_{orb(2)}$[†] | W→$N_2$ ($\pi_\perp$) | −127.2 (34.7%) | 1.22 |
| $\Delta E_{orb(3)}$[†] | $N_2$→W (σ) | −35.5 (9.7%) | 0.53 |
| $\Delta E_{orb(rest)}$[†] | - | −29.0 (7.9%) | - |

[*]The values in parentheses show the weight of each contribution with respect to the total attractive interaction.
[†]The values in parentheses show the weight of each contribution with respect to the total orbital interaction, $\Delta E_{orb}$.

**Table S4.** EDA-NOCV results for **2k**. Energies are given in kcal mol$^{-1}$.

| Energy Terms | Orbital Interaction | Energy $[BrWN_2BDur]^{+}$ + DMAP | NOCV Eigenvalue, $\nu_k$ |
|---|---|---|---|
| $\Delta E_{int}$ | - | -84.2 | - |
| $\Delta E_{Pauli}$ | - | 236.3 | - |
| $\Delta E_{disp}$[*] | - | -14.9 (4.6%) | - |
| $\Delta E_{elstat}$[*] | - | -157.1 (49.0%) | - |
| $\Delta E_{orb}$[*] | - | -148.4 (46.3%) | - |
| $\Delta E_{orb(1)}$[†] | N→B (σ) | -102.8 (69.2%) | 0.75 |
| $\Delta E_{orb(2)}$[†] | N–B (π) | -16.2 (10.9%) | 0.41 |
| $\Delta E_{orb(rest)}$[†] | - | -29.5 (19.9%) | - |

[*]The values in parentheses show the weight of each contribution with respect to the total attractive interaction.
[†]The values in parentheses show the weight of each contribution with respect to the total orbital interaction, $\Delta E_{orb}$.

## Cartesian Coordinates

**1**

**E = –3660.550175 $E_h$**

**ΔΔG(298.15) = 0.779426 $E_h$**

**Lowest frequency = 17.02 $cm^{-1}$**

| | | | |
|---|---|---|---|
| C | 3.545001000 | 1.004960000 | -3.483797000 |
| H | 3.531198000 | 2.097352000 | -3.465367000 |
| C | -2.357554000 | 2.711557000 | -1.055018000 |
| C | 4.436889000 | -1.052753000 | -4.393278000 |
| H | 5.099257000 | -1.569450000 | -5.092400000 |
| C | 4.393853000 | 0.347027000 | -4.372385000 |
| H | 5.025887000 | 0.925931000 | -5.050582000 |
| C | 4.061716000 | 2.022497000 | -0.390853000 |
| H | 4.464957000 | 1.085235000 | -0.778043000 |
| C | -2.903151000 | 2.808885000 | 0.234317000 |
| H | -2.864214000 | 1.950118000 | 0.900033000 |
| C | -2.400728000 | 3.840131000 | -1.889856000 |
| H | -1.991638000 | 3.799615000 | -2.899735000 |
| C | 3.036754000 | 4.407718000 | 0.633264000 |
| H | 2.625621000 | 5.335082000 | 1.039119000 |
| C | -2.819203000 | -0.825228000 | -3.211739000 |
| H | -1.824105000 | -0.995439000 | -3.622828000 |
| C | -3.490479000 | 3.995842000 | 0.675110000 |
| H | -3.910572000 | 4.040327000 | 1.681843000 |
| C | 4.884691000 | 2.879318000 | 0.341096000 |
| H | 5.927307000 | 2.605796000 | 0.519962000 |
| C | 4.376828000 | 4.076900000 | 0.852446000 |
| H | 5.021323000 | 4.746176000 | 1.427379000 |
| C | -4.323079000 | 0.345425000 | -1.723527000 |
| H | -4.509856000 | 1.115389000 | -0.974613000 |
| C | -2.975141000 | 5.033084000 | -1.444030000 |
| H | -2.996865000 | 5.902598000 | -2.105806000 |
| C | -3.524635000 | 5.113899000 | -0.161289000 |
| H | -3.974228000 | 6.047730000 | 0.185037000 |
| C | -3.872475000 | -1.640886000 | -3.632608000 |
| H | -3.686204000 | -2.417996000 | -4.377773000 |

| | | | |
|---|---|---|---|
| C | -5.151613000 | -1.473020000 | -3.095861000 |
| H | -5.973331000 | -2.116038000 | -3.419857000 |
| C | -5.371988000 | -0.475843000 | -2.141060000 |
| H | -6.369262000 | -0.334027000 | -1.717031000 |
| C | 2.717564000 | 2.352822000 | -0.629540000 |
| C | 2.211771000 | 3.552159000 | -0.101036000 |
| H | 1.162794000 | 3.817233000 | -0.242167000 |
| C | -3.031163000 | 0.177761000 | -2.249441000 |
| C | 2.791061000 | -1.120198000 | -2.617054000 |
| H | 2.187627000 | -1.697587000 | -1.924232000 |
| C | 2.728675000 | 0.277042000 | -2.597680000 |
| C | 3.636408000 | -1.782089000 | -3.514620000 |
| H | 3.667947000 | -2.873018000 | -3.510900000 |
| P | -1.597772000 | 1.106200000 | -1.555170000 |
| P | 1.579373000 | 1.155461000 | -1.443933000 |
| C | 0.660565000 | 2.255813000 | -2.671681000 |
| H | 0.436060000 | 3.207497000 | -2.168229000 |
| H | 1.300776000 | 2.478693000 | -3.536582000 |
| C | -0.628813000 | 1.557135000 | -3.090410000 |
| H | -0.393880000 | 0.608420000 | -3.597497000 |
| H | -1.227845000 | 2.156635000 | -3.791648000 |
| W | -0.046327000 | 0.000019000 | 0.000023000 |
| N | -0.132788000 | 1.610476000 | 1.224426000 |
| N | -0.229129000 | 2.511611000 | 1.903688000 |
| N | -0.229365000 | -2.511810000 | -1.903298000 |
| N | -0.132904000 | -1.610543000 | -1.224229000 |
| P | 1.579445000 | -1.155444000 | 1.443942000 |
| C | 0.660633000 | -2.255810000 | 2.671661000 |
| H | 0.436138000 | -3.207494000 | 2.168203000 |
| H | 1.300824000 | -2.478716000 | 3.536575000 |
| C | -0.628733000 | -1.557138000 | 3.090422000 |
| H | -0.393759000 | -0.608433000 | 3.597512000 |
| H | -1.227723000 | -2.156626000 | 3.791713000 |
| P | -1.597780000 | -1.106212000 | 1.555247000 |
| C | 2.728789000 | -0.277097000 | 2.597701000 |
| C | 2.791227000 | 1.120140000 | 2.617158000 |
| H | 2.187747000 | 1.697582000 | 1.924425000 |
| C | 3.636663000 | 1.781945000 | 3.514703000 |

| | | | |
|---|---|---|---|
| H | 3.668257000 | 2.872871000 | 3.511057000 |
| C | 4.437162000 | 1.052522000 | 4.393270000 |
| H | 5.099595000 | 1.569144000 | 5.092382000 |
| C | 4.394043000 | -0.347254000 | 4.372330000 |
| H | 5.026084000 | -0.926210000 | 5.050471000 |
| C | 3.545104000 | -1.005097000 | 3.483758000 |
| H | 3.531223000 | -2.097487000 | 3.465258000 |
| C | 2.717590000 | -2.352743000 | 0.629428000 |
| C | 4.061627000 | -2.022189000 | 0.390379000 |
| H | 4.464765000 | -1.084796000 | 0.777362000 |
| C | 4.884599000 | -2.878941000 | -0.341634000 |
| H | 5.927122000 | -2.605243000 | -0.520785000 |
| C | 4.376861000 | -4.076703000 | -0.852694000 |
| H | 5.021361000 | -4.745917000 | -1.427690000 |
| C | 3.036909000 | -4.407765000 | -0.633147000 |
| H | 2.625873000 | -5.335276000 | -1.038752000 |
| C | 2.211922000 | -3.552262000 | 0.101221000 |
| H | 1.163040000 | -3.817549000 | 0.242672000 |
| C | -2.357533000 | -2.711550000 | 1.055039000 |
| C | -2.903176000 | -2.808802000 | -0.234293000 |
| H | -2.864215000 | -1.950005000 | -0.899976000 |
| C | -3.490539000 | -3.995721000 | -0.675127000 |
| H | -3.910676000 | -4.040148000 | -1.681845000 |
| C | -3.524673000 | -5.113831000 | 0.161207000 |
| H | -3.974306000 | -6.047631000 | -0.185156000 |
| C | -2.975101000 | -5.033110000 | 1.443913000 |
| H | -2.996775000 | -5.902673000 | 2.105631000 |
| C | -2.400648000 | -3.840190000 | 1.889788000 |
| H | -1.991485000 | -3.799769000 | 2.899640000 |
| C | -3.031174000 | -0.177798000 | 2.249515000 |
| C | -2.819236000 | 0.825162000 | 3.211851000 |
| H | -1.824156000 | 0.995329000 | 3.622996000 |
| C | -3.872505000 | 1.640846000 | 3.632671000 |
| H | -3.686266000 | 2.417941000 | 4.377859000 |
| C | -5.151618000 | 1.473030000 | 3.095849000 |
| H | -5.973328000 | 2.116078000 | 3.419804000 |
| C | -5.371973000 | 0.475871000 | 2.141024000 |
| H | -6.369226000 | 0.334094000 | 1.716938000 |

| | | | |
|---|---|---|---|
| C | -4.323065000 | -0.345414000 | 1.723524000 |
| H | -4.509824000 | -1.115356000 | 0.974583000 |

**2b**

**E = –9073.234042 $E_h$**

**ΔΔG(298.15) = 0.934717 $E_h$**

**Lowest frequency = 14.98 $cm^{-1}$**

| | | | |
|---|---|---|---|
| W | 0.684538000 | -0.093428000 | 0.224696000 |
| Br | 3.158255000 | -0.699070000 | 1.068013000 |
| Br | -2.819098000 | -0.563196000 | -3.202636000 |
| P | -0.067657000 | -1.243181000 | 2.351935000 |
| P | 1.546299000 | 1.176592000 | -1.743470000 |
| P | 1.016006000 | 1.751138000 | 1.911316000 |
| P | 0.789813000 | -1.910209000 | -1.504965000 |
| N | -1.014263000 | 0.236083000 | -0.286845000 |
| N | -2.218195000 | 0.467126000 | -0.503770000 |
| C | -1.869348000 | -1.620183000 | 2.476891000 |
| C | -0.348016000 | 2.930633000 | 2.294243000 |
| C | 3.338267000 | 1.203160000 | -2.125931000 |
| C | 1.263594000 | 0.941344000 | 3.573184000 |
| H | 2.231988000 | 0.423926000 | 3.537737000 |
| H | 1.295688000 | 1.704906000 | 4.364998000 |
| C | 0.747387000 | 0.387801000 | -3.218050000 |
| H | -0.319401000 | 0.634947000 | -3.121911000 |
| H | 1.103099000 | 0.850262000 | -4.151641000 |
| C | 1.051404000 | 2.940025000 | -1.919685000 |
| C | 0.933047000 | -1.127969000 | -3.226877000 |
| H | 1.920845000 | -1.406036000 | -3.618676000 |
| H | 0.174146000 | -1.590440000 | -3.871620000 |
| C | -2.359575000 | -2.927813000 | 2.620030000 |
| H | -1.666701000 | -3.765515000 | 2.704540000 |
| C | -0.629447000 | -3.058596000 | -1.706015000 |
| C | 1.924341000 | 3.923720000 | -2.404868000 |
| H | 2.929860000 | 3.650257000 | -2.726822000 |
| C | 0.710480000 | -2.799627000 | 2.944278000 |
| C | 0.134801000 | -0.057125000 | 3.803999000 |

| | | | |
|---|---|---|---|
| H | -0.825212000 | 0.470318000 | 3.906517000 |
| H | 0.292578000 | -0.626872000 | 4.729863000 |
| C | 4.223830000 | 1.650594000 | -1.132354000 |
| H | 3.838531000 | 1.972010000 | -0.169013000 |
| C | -1.613529000 | -3.123464000 | -0.716886000 |
| H | -1.545859000 | -2.464967000 | 0.144921000 |
| C | -1.613194000 | 2.794416000 | 1.708435000 |
| H | -1.801586000 | 2.018376000 | 0.970544000 |
| C | 2.229620000 | -3.059901000 | -1.510734000 |
| C | -2.786682000 | -0.563913000 | 2.347573000 |
| H | -2.430764000 | 0.455263000 | 2.215723000 |
| C | -0.237104000 | 3.311366000 | -1.502028000 |
| H | -0.923333000 | 2.556101000 | -1.122711000 |
| C | -4.696847000 | 0.714710000 | -1.133100000 |
| B | -3.218979000 | 0.266600000 | -1.421683000 |
| C | 1.463905000 | -3.567062000 | 2.048805000 |
| H | 1.624586000 | -3.201963000 | 1.038121000 |
| C | 2.448253000 | 2.890470000 | 1.753342000 |
| C | 3.857600000 | 0.787048000 | -3.361486000 |
| H | 3.194127000 | 0.443819000 | -4.155560000 |
| C | 2.311437000 | 4.053183000 | 0.976264000 |
| H | 1.344527000 | 4.310242000 | 0.543194000 |
| C | -5.026550000 | 2.088341000 | -1.113245000 |
| C | -3.736619000 | -3.166136000 | 2.646049000 |
| H | -4.103145000 | -4.189032000 | 2.763425000 |
| C | 3.515635000 | -2.538128000 | -1.729563000 |
| H | 3.652443000 | -1.475841000 | -1.928618000 |
| C | -2.685244000 | -4.011786000 | -0.839846000 |
| H | -3.439084000 | -4.052599000 | -0.052371000 |
| C | -0.636285000 | 4.647378000 | -1.549246000 |
| H | -1.635513000 | 4.923115000 | -1.207178000 |
| C | 1.522101000 | 5.261648000 | -2.450138000 |
| H | 2.214776000 | 6.021684000 | -2.820111000 |
| C | -6.319121000 | 2.489431000 | -0.751509000 |
| H | -6.562203000 | 3.556723000 | -0.740823000 |
| C | -7.307593000 | 1.559829000 | -0.407152000 |
| C | -0.735287000 | -3.890471000 | -2.833416000 |
| H | 0.034005000 | -3.865842000 | -3.608509000 |

| | | | |
|---|---|---|---|
| C | -2.656321000 | 3.656638000 | 2.065432000 |
| H | -3.638966000 | 3.525485000 | 1.606515000 |
| C | 0.523858000 | -3.269038000 | 4.255276000 |
| H | -0.084382000 | -2.701633000 | 4.963166000 |
| C | -0.138722000 | 3.957257000 | 3.232538000 |
| H | 0.846536000 | 4.089077000 | 3.685540000 |
| C | -3.995095000 | 3.128899000 | -1.479986000 |
| H | -4.449944000 | 4.124112000 | -1.593844000 |
| H | -3.485055000 | 2.876339000 | -2.423093000 |
| H | -3.212351000 | 3.201201000 | -0.710585000 |
| C | 5.597457000 | 1.680734000 | -1.369873000 |
| H | 6.267076000 | 2.033136000 | -0.582422000 |
| C | -5.694093000 | -0.233517000 | -0.814088000 |
| C | 2.026744000 | -4.780477000 | 2.450914000 |
| H | 2.617852000 | -5.356579000 | 1.736080000 |
| C | 2.092629000 | -4.425362000 | -1.218090000 |
| H | 1.104487000 | -4.847501000 | -1.031854000 |
| C | 5.236144000 | 0.803541000 | -3.592942000 |
| H | 5.626269000 | 0.469117000 | -4.557181000 |
| C | -6.974110000 | 0.200743000 | -0.448229000 |
| H | -7.733531000 | -0.543931000 | -0.189423000 |
| C | 3.701630000 | 2.584301000 | 2.306757000 |
| H | 3.836994000 | 1.676658000 | 2.892772000 |
| C | -1.813134000 | -4.764785000 | -2.965496000 |
| H | -1.889540000 | -5.403339000 | -3.848930000 |
| C | -1.176586000 | 4.821674000 | 3.580450000 |
| H | -0.998847000 | 5.616681000 | 4.308801000 |
| C | 6.109128000 | 1.249624000 | -2.597298000 |
| H | 7.186573000 | 1.262023000 | -2.778979000 |
| C | 0.245983000 | 5.628054000 | -2.015148000 |
| H | -0.062864000 | 6.675830000 | -2.040216000 |
| C | 1.087885000 | -4.480039000 | 4.659668000 |
| H | 0.934321000 | -4.835902000 | 5.681396000 |
| C | 3.409167000 | 4.882813000 | 0.745492000 |
| H | 3.285409000 | 5.774009000 | 0.126890000 |
| C | -4.160621000 | -0.804021000 | 2.366884000 |
| H | -4.855665000 | 0.027305000 | 2.234323000 |
| C | -4.639829000 | -2.107434000 | 2.519308000 |

| | | | |
|---|---|---|---|
| H | -5.715442000 | -2.297501000 | 2.524782000 |
| C | 1.842945000 | -5.237763000 | 3.757350000 |
| H | 2.286581000 | -6.184286000 | 4.076113000 |
| C | -2.790997000 | -4.828398000 | -1.965969000 |
| H | -3.634540000 | -5.515251000 | -2.068986000 |
| C | 4.635790000 | -3.367668000 | -1.675383000 |
| H | 5.626935000 | -2.941128000 | -1.845567000 |
| C | 4.797295000 | 3.421371000 | 2.078411000 |
| H | 5.767667000 | 3.167622000 | 2.511762000 |
| C | -5.383428000 | -1.707631000 | -0.867649000 |
| H | -6.199104000 | -2.309842000 | -0.441161000 |
| H | -4.464931000 | -1.937473000 | -0.311148000 |
| H | -5.213028000 | -2.039018000 | -1.904523000 |
| C | 4.491408000 | -4.727785000 | -1.388175000 |
| H | 5.369698000 | -5.376132000 | -1.341289000 |
| C | -2.441150000 | 4.670733000 | 2.999544000 |
| H | -3.254950000 | 5.345465000 | 3.276121000 |
| C | 4.656403000 | 4.568362000 | 1.292924000 |
| H | 5.516530000 | 5.216202000 | 1.107792000 |
| C | 3.217437000 | -5.253026000 | -1.157755000 |
| H | 3.093175000 | -6.315677000 | -0.934320000 |
| C | -8.702087000 | 2.006227000 | -0.045517000 |
| H | -9.176430000 | 1.313387000 | 0.666647000 |
| H | -9.350049000 | 2.047598000 | -0.938823000 |
| H | -8.701953000 | 3.011824000 | 0.402495000 |

**2f**

**E = –4884.929912 $E_h$**

**ΔΔG(298.15) = 0.965157 $E_h$**

**Lowest frequency = 14.80 $cm^{-1}$**

| | | | |
|---|---|---|---|
| W | 0.856475000 | 0.048740000 | 0.222760000 |
| C | -4.433196000 | -0.073843000 | -1.596865000 |
| Cl | -2.319091000 | -0.208163000 | -3.641024000 |
| B | -2.884150000 | -0.038900000 | -1.895793000 |
| N | -0.801914000 | 0.071003000 | -0.503015000 |
| N | -1.981112000 | 0.085041000 | -0.873681000 |

| | | | |
|---|---|---|---|
| Cl | 3.162843000 | -0.013009000 | 1.309770000 |
| C | -6.384425000 | -1.338671000 | -0.856178000 |
| C | -7.081462000 | -0.132615000 | -0.732944000 |
| H | -8.116283000 | -0.154387000 | -0.376909000 |
| C | -6.508782000 | 1.098910000 | -1.069334000 |
| C | -5.168689000 | 1.126159000 | -1.502997000 |
| C | -7.047331000 | -2.644524000 | -0.497530000 |
| H | -7.137688000 | -3.310669000 | -1.372722000 |
| H | -8.057752000 | -2.482843000 | -0.094386000 |
| H | -6.465346000 | -3.201242000 | 0.256276000 |
| C | -7.318547000 | 2.367129000 | -0.961972000 |
| H | -7.378387000 | 2.896892000 | -1.927510000 |
| H | -6.874699000 | 3.078073000 | -0.243305000 |
| H | -8.345840000 | 2.156720000 | -0.630339000 |
| C | -4.526836000 | 2.428965000 | -1.909925000 |
| H | -3.432507000 | 2.359584000 | -1.892177000 |
| H | -4.815721000 | 3.272324000 | -1.264384000 |
| H | -4.816674000 | 2.708853000 | -2.938376000 |
| C | -4.298059000 | -2.602927000 | -1.497544000 |
| H | -4.893402000 | -3.339862000 | -2.060315000 |
| H | -4.044822000 | -3.063852000 | -0.528722000 |
| H | -3.363897000 | -2.449561000 | -2.051362000 |
| C | -5.052369000 | -1.309395000 | -1.316286000 |
| P | 1.632161000 | -1.459195000 | -1.592197000 |
| C | 1.317630000 | -0.545580000 | -3.182839000 |
| H | 1.707535000 | -1.112610000 | -4.042321000 |
| H | 0.223102000 | -0.515010000 | -3.284933000 |
| P | 1.557204000 | 1.726936000 | -1.483724000 |
| C | 1.910791000 | 0.862151000 | -3.117978000 |
| H | 1.576840000 | 1.490026000 | -3.956617000 |
| H | 3.007621000 | 0.822242000 | -3.173217000 |
| P | 0.346997000 | 1.517232000 | 2.175594000 |
| C | 0.953950000 | 0.626920000 | 3.696579000 |
| H | 2.047806000 | 0.676571000 | 3.612451000 |
| H | 0.661262000 | 1.173641000 | 4.605611000 |
| P | 0.380807000 | -1.638883000 | 2.021381000 |
| C | 0.475917000 | -0.827233000 | 3.730946000 |
| H | -0.547503000 | -0.888737000 | 4.124685000 |

| | | | |
|---|---|---|---|
| H | 1.106743000 | -1.438802000 | 4.390755000 |
| C | 0.220226000 | 2.927709000 | -1.849072000 |
| C | -0.579830000 | 2.841545000 | -2.995399000 |
| H | -0.428107000 | 2.046446000 | -3.723969000 |
| C | -1.616172000 | 3.756389000 | -3.204279000 |
| H | -2.238941000 | 3.663840000 | -4.096494000 |
| C | -1.869445000 | 4.761979000 | -2.270005000 |
| H | -2.683187000 | 5.471936000 | -2.434980000 |
| C | -1.090295000 | 4.839668000 | -1.110817000 |
| H | -1.286898000 | 5.609595000 | -0.361115000 |
| C | -0.058749000 | 3.925714000 | -0.898180000 |
| H | 0.534689000 | 3.991004000 | 0.012971000 |
| C | 3.075757000 | 2.756325000 | -1.330068000 |
| C | 3.099805000 | 4.142913000 | -1.535970000 |
| H | 2.186827000 | 4.675302000 | -1.801726000 |
| C | 4.293602000 | 4.856465000 | -1.399056000 |
| H | 4.295976000 | 5.937491000 | -1.558766000 |
| C | 5.475553000 | 4.194181000 | -1.059657000 |
| H | 6.407111000 | 4.754702000 | -0.948688000 |
| C | 5.461613000 | 2.808659000 | -0.869092000 |
| H | 6.380409000 | 2.278906000 | -0.606809000 |
| C | 4.272002000 | 2.092064000 | -1.004656000 |
| H | 4.269397000 | 1.015377000 | -0.832656000 |
| C | 3.407751000 | -1.916375000 | -1.664547000 |
| C | 4.218767000 | -1.676658000 | -2.784171000 |
| H | 3.806643000 | -1.213504000 | -3.680985000 |
| C | 5.569669000 | -2.037459000 | -2.773178000 |
| H | 6.188894000 | -1.839977000 | -3.651558000 |
| C | 6.121995000 | -2.652411000 | -1.647143000 |
| H | 7.177609000 | -2.934928000 | -1.639558000 |
| C | 5.317653000 | -2.904083000 | -0.531687000 |
| H | 5.734647000 | -3.383531000 | 0.356503000 |
| C | 3.974364000 | -2.531404000 | -0.536254000 |
| H | 3.363380000 | -2.712827000 | 0.342943000 |
| C | 0.775857000 | -3.069583000 | -1.833282000 |
| C | 1.449318000 | -4.202498000 | -2.312899000 |
| H | 2.498141000 | -4.129690000 | -2.604741000 |
| C | 0.793547000 | -5.434301000 | -2.388299000 |

| | | | |
|---|---|---|---|
| H | 1.332131000 | -6.312842000 | -2.752263000 |
| C | -0.541248000 | -5.544021000 | -1.989901000 |
| H | -1.050642000 | -6.509565000 | -2.037231000 |
| C | -1.223011000 | -4.413959000 | -1.527195000 |
| H | -2.262118000 | -4.493107000 | -1.205748000 |
| C | -0.570084000 | -3.182707000 | -1.447715000 |
| H | -1.098101000 | -2.317408000 | -1.048792000 |
| C | 1.526544000 | -3.071494000 | 2.125211000 |
| C | 1.316958000 | -4.195706000 | 1.306354000 |
| H | 0.425127000 | -4.263036000 | 0.685764000 |
| C | 2.254032000 | -5.228571000 | 1.263887000 |
| H | 2.072137000 | -6.087391000 | 0.614447000 |
| C | 3.420946000 | -5.152729000 | 2.029895000 |
| H | 4.158521000 | -5.957744000 | 1.989960000 |
| C | 3.643111000 | -4.035132000 | 2.838146000 |
| H | 4.557984000 | -3.958514000 | 3.430506000 |
| C | 2.704627000 | -3.000553000 | 2.885279000 |
| H | 2.915477000 | -2.120177000 | 3.491650000 |
| C | -1.308116000 | -2.370850000 | 2.108985000 |
| C | -1.561822000 | -3.660189000 | 2.605475000 |
| H | -0.735988000 | -4.310625000 | 2.895444000 |
| C | -2.875054000 | -4.118239000 | 2.739419000 |
| H | -3.057851000 | -5.125110000 | 3.123112000 |
| C | -3.949873000 | -3.292002000 | 2.395618000 |
| H | -4.975775000 | -3.652869000 | 2.503954000 |
| C | -3.705818000 | -2.003388000 | 1.914521000 |
| H | -4.530194000 | -1.347087000 | 1.629004000 |
| C | -2.394198000 | -1.547899000 | 1.768049000 |
| H | -2.228021000 | -0.547878000 | 1.380809000 |
| C | -1.448416000 | 1.751914000 | 2.465960000 |
| C | -2.055133000 | 1.469850000 | 3.701044000 |
| H | -1.451306000 | 1.179571000 | 4.560835000 |
| C | -3.441774000 | 1.544921000 | 3.846099000 |
| H | -3.898517000 | 1.305916000 | 4.809422000 |
| C | -4.239947000 | 1.914490000 | 2.760771000 |
| H | -5.326711000 | 1.955694000 | 2.864358000 |
| C | -3.643208000 | 2.225464000 | 1.536840000 |
| H | -4.259970000 | 2.505492000 | 0.685380000 |

| | | | |
|---|---|---|---|
| C | -2.257846000 | 2.144532000 | 1.386548000 |
| H | -1.806089000 | 2.363222000 | 0.420372000 |
| C | 1.061960000 | 3.201697000 | 2.363628000 |
| C | 0.290596000 | 4.301055000 | 2.770628000 |
| H | -0.754211000 | 4.159516000 | 3.052582000 |
| C | 0.850066000 | 5.582138000 | 2.791109000 |
| H | 0.239267000 | 6.434325000 | 3.099715000 |
| C | 2.182410000 | 5.774089000 | 2.414188000 |
| H | 2.614226000 | 6.777917000 | 2.422102000 |
| C | 2.958985000 | 4.678800000 | 2.022674000 |
| H | 3.996950000 | 4.818613000 | 1.714254000 |
| C | 2.403284000 | 3.399154000 | 1.993120000 |
| H | 3.000904000 | 2.549865000 | 1.658552000 |

**2k**

**E = –6920.511414 $E_h$**

**ΔΔG(298.15) = 1.122083 $E_h$**

**Lowest frequency = 11.20 $cm^{-1}$**

| | | | |
|---|---|---|---|
| W | -0.856662000 | -0.422802000 | 0.281732000 |
| B | 2.849485000 | 1.423952000 | -0.395612000 |
| N | 2.011672000 | 0.612977000 | 0.332075000 |
| Br | -3.077020000 | -1.898033000 | 0.215765000 |
| N | 0.770869000 | 0.357569000 | 0.296473000 |
| N | 4.343163000 | 1.196484000 | -0.095608000 |
| C | 6.066055000 | -0.145973000 | 0.885212000 |
| H | 6.298156000 | -1.025199000 | 1.479705000 |
| N | 8.398397000 | 0.466167000 | 0.590024000 |
| C | 7.087431000 | 0.706043000 | 0.369965000 |
| C | 6.638078000 | 1.823201000 | -0.394059000 |
| H | 7.336149000 | 2.533627000 | -0.830146000 |
| C | 5.294411000 | 2.024208000 | -0.599292000 |
| H | 4.921584000 | 2.863830000 | -1.186479000 |
| C | 8.808982000 | -0.688329000 | 1.379270000 |

| | | | |
|---|---|---|---|
| H | 9.901925000 | -0.706092000 | 1.451390000 |
| H | 8.480681000 | -1.632827000 | 0.914558000 |
| H | 8.399315000 | -0.643663000 | 2.401853000 |
| C | 9.411456000 | 1.357220000 | 0.037832000 |
| H | 9.300547000 | 2.383581000 | 0.424953000 |
| H | 9.360244000 | 1.392909000 | -1.062909000 |
| H | 10.405252000 | 0.992974000 | 0.320257000 |
| C | 4.744659000 | 0.122985000 | 0.635235000 |
| H | 3.936684000 | -0.502874000 | 1.008573000 |
| C | 2.507224000 | 2.512402000 | -1.476806000 |
| C | 2.052000000 | 3.783761000 | -1.060697000 |
| C | 1.597277000 | 4.711948000 | -2.018875000 |
| C | 1.648129000 | 4.354047000 | -3.371173000 |
| H | 1.286632000 | 5.069450000 | -4.115475000 |
| C | 2.177175000 | 3.136739000 | -3.812041000 |
| C | 2.622477000 | 2.207107000 | -2.851314000 |
| C | 2.159311000 | 4.177020000 | 0.393424000 |
| H | 3.123157000 | 4.684219000 | 0.581429000 |
| H | 2.117812000 | 3.301300000 | 1.056199000 |
| H | 1.372124000 | 4.871865000 | 0.703464000 |
| C | 3.250752000 | 0.911246000 | -3.299426000 |
| H | 3.318308000 | 0.175850000 | -2.487868000 |
| H | 4.273253000 | 1.081484000 | -3.681757000 |
| H | 2.695241000 | 0.435002000 | -4.118120000 |
| C | 1.076623000 | 6.067781000 | -1.615398000 |
| H | 0.199741000 | 5.978775000 | -0.954307000 |
| H | 0.776527000 | 6.654267000 | -2.495128000 |
| H | 1.835570000 | 6.650317000 | -1.067301000 |
| C | 2.255432000 | 2.829631000 | -5.285523000 |
| H | 1.887285000 | 3.671259000 | -5.888898000 |
| H | 1.654565000 | 1.941760000 | -5.547615000 |

| | | | |
|---|---|---|---|
| H | 3.288305000 | 2.612529000 | -5.604213000 |
| P | -0.072060000 | -2.206194000 | 1.901397000 |
| C | -0.814217000 | -1.873966000 | 3.603390000 |
| H | -0.919193000 | -2.822511000 | 4.146761000 |
| H | -0.082519000 | -1.266728000 | 4.154117000 |
| P | -1.977271000 | 0.345457000 | 2.415706000 |
| P | -0.289767000 | -1.309800000 | -2.009022000 |
| C | -0.448314000 | 0.110193000 | -3.258448000 |
| H | 0.471630000 | 0.148436000 | -3.856087000 |
| H | -1.263809000 | -0.169223000 | -3.938671000 |
| P | -1.880910000 | 1.336069000 | -1.182262000 |
| C | -0.714858000 | 1.470896000 | -2.612458000 |
| H | -1.068984000 | 2.206854000 | -3.349184000 |
| H | 0.202410000 | 1.881458000 | -2.174713000 |
| C | -2.145812000 | -1.140617000 | 3.526385000 |
| H | -2.488330000 | -0.821862000 | 4.522496000 |
| H | -2.912774000 | -1.787076000 | 3.080309000 |
| C | -3.672911000 | 1.049601000 | 2.384495000 |
| C | -4.803626000 | 0.218445000 | 2.451277000 |
| H | -4.693912000 | -0.856933000 | 2.577982000 |
| C | -6.085788000 | 0.757614000 | 2.318713000 |
| H | -6.954075000 | 0.096876000 | 2.370383000 |
| C | -6.256639000 | 2.128929000 | 2.113362000 |
| H | -7.259685000 | 2.546645000 | 2.001076000 |
| C | -5.136983000 | 2.963357000 | 2.057568000 |
| H | -5.254884000 | 4.037397000 | 1.900559000 |
| C | -3.855313000 | 2.429895000 | 2.196634000 |
| H | -2.995317000 | 3.095135000 | 2.154760000 |
| C | -1.051241000 | 1.526267000 | 3.483510000 |
| C | -1.657286000 | 2.076033000 | 4.625798000 |
| H | -2.693029000 | 1.827894000 | 4.867330000 |

| | | | |
|---|---|---|---|
| C | -0.950539000 | 2.955963000 | 5.447115000 |
| H | -1.434754000 | 3.380372000 | 6.329571000 |
| C | 0.371092000 | 3.297030000 | 5.140047000 |
| H | 0.920666000 | 3.989291000 | 5.782064000 |
| C | 0.982455000 | 2.749227000 | 4.009862000 |
| H | 2.014044000 | 3.009533000 | 3.760836000 |
| C | 0.275253000 | 1.868546000 | 3.186254000 |
| H | 0.753266000 | 1.451832000 | 2.302137000 |
| C | -0.394437000 | -3.993366000 | 1.622181000 |
| C | -0.230781000 | -4.937492000 | 2.650238000 |
| H | 0.076635000 | -4.621367000 | 3.649495000 |
| C | -0.432658000 | -6.295265000 | 2.400087000 |
| H | -0.303841000 | -7.020560000 | 3.206827000 |
| C | -0.796290000 | -6.726031000 | 1.119099000 |
| H | -0.958912000 | -7.789029000 | 0.926185000 |
| C | -0.951479000 | -5.793886000 | 0.091336000 |
| H | -1.242677000 | -6.113942000 | -0.910939000 |
| C | -0.751657000 | -4.434694000 | 0.343608000 |
| H | -0.893971000 | -3.708362000 | -0.451181000 |
| C | 1.723971000 | -2.230550000 | 2.352661000 |
| C | 2.561321000 | -3.285945000 | 1.957548000 |
| H | 2.161423000 | -4.100254000 | 1.352418000 |
| C | 3.901460000 | -3.319539000 | 2.354742000 |
| H | 4.529996000 | -4.161566000 | 2.054872000 |
| C | 4.423005000 | -2.302599000 | 3.159126000 |
| H | 5.458628000 | -2.350670000 | 3.505117000 |
| C | 3.606828000 | -1.228970000 | 3.527963000 |
| H | 4.006359000 | -0.418097000 | 4.141409000 |
| C | 2.273720000 | -1.186570000 | 3.115238000 |
| H | 1.658879000 | -0.335464000 | 3.406368000 |
| C | -3.531563000 | 1.086932000 | -1.935882000 |

| | | | |
|---|---|---|---|
| C | -3.751553000 | 1.173209000 | -3.319714000 |
| H | -2.931223000 | 1.398608000 | -4.001554000 |
| C | -5.029940000 | 0.970763000 | -3.847534000 |
| H | -5.185624000 | 1.034141000 | -4.926894000 |
| C | -6.103629000 | 0.693855000 | -2.997167000 |
| H | -7.102335000 | 0.535317000 | -3.410542000 |
| C | -5.894820000 | 0.626060000 | -1.616569000 |
| H | -6.725924000 | 0.415415000 | -0.940397000 |
| C | -4.618193000 | 0.816352000 | -1.089168000 |
| H | -4.466413000 | 0.750113000 | -0.015792000 |
| C | -1.994906000 | 3.054528000 | -0.535012000 |
| C | -3.008465000 | 3.937453000 | -0.936044000 |
| H | -3.723875000 | 3.636210000 | -1.702487000 |
| C | -3.129535000 | 5.191422000 | -0.331325000 |
| H | -3.928279000 | 5.867725000 | -0.644522000 |
| C | -2.248794000 | 5.570289000 | 0.686114000 |
| H | -2.361762000 | 6.539782000 | 1.176707000 |
| C | -1.226565000 | 4.699693000 | 1.076896000 |
| H | -0.546595000 | 4.977130000 | 1.884994000 |
| C | -1.093221000 | 3.455217000 | 0.459452000 |
| H | -0.314918000 | 2.766983000 | 0.780377000 |
| C | -1.328980000 | -2.610257000 | -2.789676000 |
| C | -0.845059000 | -3.906426000 | -3.024735000 |
| H | 0.188754000 | -4.159080000 | -2.787656000 |
| C | -1.684297000 | -4.887961000 | -3.558869000 |
| H | -1.291555000 | -5.890802000 | -3.743352000 |
| C | -3.014641000 | -4.588196000 | -3.861959000 |
| H | -3.668720000 | -5.356518000 | -4.280352000 |
| C | -3.504294000 | -3.301049000 | -3.624708000 |
| H | -4.545124000 | -3.056246000 | -3.847033000 |
| C | -2.671216000 | -2.320405000 | -3.086441000 |

| | | | |
|---|---|---|---|
| H | -3.080122000 | -1.331180000 | -2.886608000 |
| C | 1.417445000 | -1.923103000 | -2.313030000 |
| C | 1.862413000 | -2.207381000 | -3.615868000 |
| H | 1.187582000 | -2.079383000 | -4.465324000 |
| C | 3.159719000 | -2.669931000 | -3.834678000 |
| H | 3.493521000 | -2.891761000 | -4.850913000 |
| C | 4.030524000 | -2.853078000 | -2.753685000 |
| H | 5.046698000 | -3.215182000 | -2.927093000 |
| C | 3.591566000 | -2.583213000 | -1.456141000 |
| H | 4.256372000 | -2.734689000 | -0.603219000 |
| C | 2.289274000 | -2.123052000 | -1.238324000 |
| H | 1.947817000 | -1.907173000 | -0.230283000 |

## References


[1] a) S. H. Vosko, L. Wilk, M. Nusair, *Can. J. Phys.* **1980**, *58*, 1200–1211; b) C. Lee, W. Yang, R. G. Parr, *Phys. Rev. B* **1988**, *37*, 785–789; c) A. D. Becke, *J. Chem. Phys.* **1993**, *98*, 5648–5652; d) P. J. Stephens, F. J. Devlin, C. F. Chabalowski, M. J. Frisch, *J. Phys. Chem.* **1994**, *98*, 11623–11627.

[2] S. Grimme, J. Antony, S. Ehrlich, H. Krieg, *J. Chem. Phys*. **2010**, *132*, 154104.

[3] a) A. D. Becke, E. R. Johnson. *J. Chem. Phys.* **2005**, *122,* 154101. b) E. R. Johnson, A. D. Becke. *J. Chem. Phys.* **2005**, *123*, 024101. c) E. R. Johnson, A. D. Becke. *J. Chem. Phys.* **2006**, *124*, 174104.

[4] S. Grimme, S. Ehrlich, L. Goerigk, *J. Comput. Chem.* **2011**, *32*, 1456–1465.

[5] F. Weigend, R. Ahlrichs, *Phys. Chem. Chem. Phys.* **2005**, *7*, 3297.

[6] D. Andrae, U. Häußermann, M. Dolg, H. Stoll, H. Preuß, H. *Theor. Chim. Acta* **1990**, *77*, 123–141.

[7] J. M. L. Martin, A. Sundermann, *J. Chem. Phys.* **2001**, *114*, 3408–3420.

[8] a) M. P. Mitoraj, A. Michalak, T. Ziegler, *J. Chem. Theory Comput.* **2009**, *5*, 962–975; b) L. Zhao, M. von Hopffgarten, D. M. Andrada, G. Frenking, *WIREs Comput. Mol. Sci.* **2018**, *8*, e1345.

[9] a) E. van Lenthe, E. J. Baerends, J. G. Snijders, *J. Chem. Phys.* **1993**, *99*, 4597–4610; b) E. van Lenthe, E. J. Baerends, J. G. Snijders, *J. Chem. Phys.* **1994**, *101*, 9783–9792; c) E. van Lenthe, A. Ehlers, E.-J. Baerends, *J. Chem. Phys.* **1999**, *110*, 8943–8953.

[10] a) I. Mayer, Chem. Phys. Lett. 1983, 97, 270–274; b) I. Mayer, *Int. J. Quant. Chem.* **1984**, *26*, 151–154.

[11] M. J. Frisch, G. W. Trucks, H. B. Schlegel, G. E. Scuseria, M. A. Robb, J. R. Cheeseman, G. Scalmani, V. Barone, B. Mennucci, G. A. Petersson, H. Nakatsuji, M. Caricato, X. Li, H. P. Hratchian, A. F. Izmaylov, J. Bloino, G. Zheng, J. L. Sonnenberg, M. Hada, M. Ehara, K. Toyota, R. Fukuda, J. Hasegawa, M. Ishida, T. Nakajima, Y. Honda, O. Kitao, H. Nakai, T. Vreven, J. A. Montgomery, Jr., J. E. Peralta, F. Ogliaro, M. Bearpark, J. J. Heyd, E. Brothers, K. N. Kudin, V. N. Staroverov, R. Kobayashi, J. Normand, K. Raghavachari, A. Rendell, J. C. Burant, S. S. Iyengar, J. Tomasi, M. Cossi, N. Rega, J. M. Millam, M. Klene, J. E. Knox, J. B. Cross, V. Bakken, C. Adamo, J. Jaramillo, R. Gomperts, R. E. Stratmann, O. Yazyev, A. J. Austin, R. Cammi, C. Pomelli, J. W. Ochterski, R. L. Martin, K. Morokuma, V. G. Zakrzewski, G. A. Voth, P. Salvador, J. J. Dannenberg, S. Dapprich, A. D. Daniels, O. Farkas, J. B. Foresman, J. V. Ortiz, J.

Cioslowski, D. J. Fox, Gaussian 16, Revision B.01; Gaussian, Inc.: Wallingford CT, 2016.

[12] G. te Velde, F. M. Bickelhaupt, E. J. Baerends, C. Fonseca Guerra, S. J. A. van Gisbergen, J. G. Snijders, T. Ziegler, *J. Comput. Chem.* **2001**, *22*, 931–967.
